# Nurse-Led Family Support Intervention for Families of Critically Ill Patients: The FICUS Cluster Randomized Clinical Trial

**DOI:** 10.1001/jamainternmed.2025.3406

**Published:** 2025-07-28

**Authors:** Rahel Naef, Marie-Madlen Jeitziner, Marco Riguzzi, Stefanie von Felten, Lotte Verweij, Michael Rufer, Judith Safford, Simone Sutter, Bettina Bergmann-Kipfer, Ursula Betschart, Sabina Boltshauser, Nadine Brülisauer, Christoph Brunner, Philipp Karl Bühler, Hanna Burkhalter, Alexander Dullenkopf, Antje Heise, Benjamin Hertler, Johanna Elisabeth Hoffmann, Carmen Karde, Yvonne Keller, Susanne Kohler, Fabienne Lussmann, Paola Massarotto, Michaela Moser, Urs Pietsch, Diana Livia Segalada, Esther Siegrist, Peter Steiger, Naira Ruch, Christoph von Dach, Monique Seraina Wenzler, Jan Wiegand, Bjoern Zante, Miodrag Filipovic

**Affiliations:** 1 Institute for Implementation Science in Health Care, Faculty of Medicine, University of Zurich, Zurich, Switzerland; 2 Centre of Clinical Nursing Science, University Hospital Zurich, Zurich, Switzerland; 3 Department of Intensive Care Medicine, Inselspital, Bern University Hospital, Bern, Switzerland; 4 Department of Biostatistics at Epidemiology, Biostatistics and Prevention Institute, University of Zurich, Zurich, Switzerland; 5 Center for Psychiatry and Psychotherapy, Clinic Zugersee, Oberwil-Zug, Switzerland; 6 Department of Psychiatry, Psychotherapy, and Psychosomatics, Psychiatric University Hospital Zurich, University of Zurich, Zurich, Switzerland; 7 Patient Research Partner, Bern, Switzerland; 8 Intensive Care Unit, Hospital of Thun, Thun, Switzerland; 9 Department of Intensive Care Medicine, HOCH Health Ostschweiz, Kantonsspital St Gallen, St Gallen, Switzerland; 10 Department of Intensive Care Medicine, Spital Thurgau Frauenfeld, Frauenfeld, Switzerland; 11 Intensive Care Unit, Department of Anesthesia, Emergency, Intensive Care and Rescue, Cantonal Hospital Graubünden, Chur, Switzerland; 12 Division of Perioperative Intensive Care Medicine, HOCH Health Ostschweiz, Kantonsspital St Gallen, St Gallen, Switzerland; 13 Center of Intensive Care Medicine, Cantonal Hospital Winterthur, Winterthur, Switzerland; 14 Department of Nursing and Specialist Support, Nursing Development, Cantonal Hospital Graubünden, Chur, Switzerland; 15 Institute for Anesthesia, Spital Thurgau Frauenfeld, Frauenfeld, Switzerland; 16 Institute of Intensive Care Medicine, University Hospital Zurich, University of Zurich, Zurich, Switzerland; 17 Nursing Development and Quality Unit, Lucerne Cantonal Hospital, Luzern, Switzerland; 18 Nurse Development Department, Cantonal Hospital Baden, Baden, Switzerland; 19 Center of Intensive Care Medicine, Lucerne Cantonal Hospital, Luzern, Switzerland; 20 Interdisciplinary Intensive Care Unit, Department of Perioperative Medicine, Solothurn Hospitals AG, Cantonal Hospital Olten, Olten, Switzerland; 21 Department of Nursing, Solothurn Hospitals AG, Solothurn, Switzerland; 22 Institute for Anesthesia and Intensive Care Medicine, Hirslanden Clinic Zurich, Zurich, Switzerland; 23 Intensive Care Unit, Lindenhofgruppe, Lindenhofspital, Bern, Switzerland; 24 Clinic for Intensive Care Medicine, Cantonal Hospital Aarau, Aarau, Switzerland

## Abstract

**Importance:**

Among families of patients treated in intensive care units (ICUs), insufficient communication and support may increase family distress that can lead to adverse health outcomes. Family-focused care is recommended, but high-quality experimental research is lacking.

**Objective:**

To determine whether a nurse-led, interprofessional, multicomponent family support intervention improves family satisfaction, communication quality, and support to families.

**Design, Setting, and Participants:**

This study was a multicenter, cluster-randomized clinical trial with 1:1 allocation of ICUs using minimization. Family members of critically ill patients who were treated for at least 48 hours at 16 adult ICUs in 12 hospitals in Switzerland were enrolled between May 2022 and January 2024. The first follow-up after patient ICU discharge was completed in February 2024. Data of all participants were analyzed using linear mixed-effects models, with the individual as the unit of inference.

**Intervention:**

Intervention ICUs implemented a novel family nurse role that engaged and liaised with families, offered relationship-focused, psychoeducational support, and ensured interprofessional communication along the ICU trajectory. Control ICUs continued with usual care.

**Main outcomes and Measures:**

The primary outcome was the family satisfaction with ICU care through the Family Satisfaction with ICU survey (range, 0-100), assessed after patient ICU discharge. Secondary outcomes included the quality of family-clinician communication, measured by the Questionnaire on Quality of Physician-Patient Interaction (range, 1-5), and cognitive and emotional support, assessed by the Family Perceived Support Questionnaire (range, 14-70).

**Results:**

Of 2057 consecutively invited family members, 412 were enrolled in the intervention and 473 in the control arm (median age, 54 [IQR, 42-65] years; 429 partners [48%]; and 568 women [64%]). The intervention improved overall family satisfaction with ICU care by a mean difference (MD) between groups of 2.39 (95% CI, 0.31-4.47; *P* = .02). Sensitivity analyses adjusting for cluster- and individual-level characteristics in combination with multiple imputation confirmed this result. Satisfaction with involvement in decision-making was more strongly increased than satisfaction with care. Furthermore, the intervention improved communication (MD, 0.37; 95% CI; 0.16-0.58; *P* = .002), and cognitive and emotional support (MD, 8.71; 95% CI, 4.71-12.71, *P* < .001).

**Conclusions and relevance:**

This randomized clinical trial found that a nurse-led, interprofessional family support intervention in ICU had a small, statistically significant, but clinically uncertain benefit on family satisfaction, and relevantly improved quality of communication and cognitive/emotional support of family.

**Trial Registration:**

ClinicalTrials.gov Identifier: NCT05280691

## Key Points

**Question:**

Does a nurse-led, interprofessional, multicomponent family support intervention in adult intensive care units (ICUs) improve family satisfaction with care, communication, and cognitive and emotional support to families of critically ill patients compared with usual care?

**Findings:**

In this cluster randomized trial in 16 ICUs with 885 family members, the family support intervention, which started at admission and followed up with families after the ICU phase, resulted in a small, but statistically significant improvement in the Family Satisfaction with ICU survey (score, 81.78 vs 79.39 out of 100).

**Meaning:**

A nurse-led family support intervention may improve quality of family care in the ICU; incorporating it as a standard component of ICU care may be of value.

## Teaser

This cluster-randomized trial assesses whether a nurse-led, interprofessional family support intervention involving engagement, support, and interpersonal communication with family members of patients being treated in intensive care units leads satisfaction with care compared with usual care.

## Introduction

Family members of critically ill adults treated in intensive care units (ICUs) experience profound uncertainty and high distress.^1,2^ At least 20% to 30% of family members are affected by postintensive care syndrome–family and report high caregiver burden.^3,4,5,6^ Critical illness presents families with substantial challenges for which they are ill-prepared.^1,7^ Families may feel left alone and lack access to timely information, communication, and support from ICU staff.^2,8^ Poor communication and insufficient support are associated with difficult care experiences that increase family distress and dissatisfaction with ICU care^7,9,10,11^ and result in adverse health outcomes.^12,13^

To improve the quality of family care and mitigate adverse mental health outcomes, family-focused interventions, such as inviting family presence, identifying and supporting needs, or offering regular communication facilitated by specialized ICU staff have long been recommended^14,15,16^ but remain poorly implemented in ICUs.^9,17,18^ Although they show promise, the strength of evidence on their effectiveness is contested.^19,20,21,22,23,24^ Previous randomized clinical trials (RCTs) on nurse-facilitated or nurse-aided interventions have not identified improvements in reducing postintensive care syndrome among family members^25,26,27^ with the exception of one trial, which showed reduced prolonged grief disorder.^28^ Nevertheless, one of these trials showed improvements in communication and person-centeredness.^26^ Reduced health care costs and hospital readmission rates were also identified.^29,30^

To extend the evidence base, this cluster-randomized trial tested a nurse-led, interprofessional family support intervention, which combines engagement and coordinating practices, nurse-therapeutic family interventions, and structured, interprofessional communication.^31,32^ The family support intervention aimed to increase capacity for and quality of family-focused ICU care, reduce families’ burden, and improve family functioning and mental health for families of a generally critically ill population. The study hypothesis was that compared with usual care, the intervention would enhance the quality of family care, measured as improved family satisfaction, family-clinician communication, and cognitive and emotional support to families.

## Methods

### Study Design

We conducted the Family Support in Intensive Care Units (FICUS) RCT at 12 hospitals across German-speaking Switzerland to compare the family support intervention with usual care provided to families. The study protocol and statistical analysis plan were published and are available in Supplement 1.^31,33^ The trial adopted a patient and public engagement strategy, was registered on ClinicalTrials.gov (April 3, 2022), and was approved by all appropriate Swiss cantonal ethics committees (No. 2021-02300). The applicable Consolidated Standards of Reporting Trials (CONSORT) reporting guidelines^34,35^ and template for intervention description and replication checklist^36^ were followed.

### Participants

All types of Swiss certified ICUs were eligible if they operated at least 8 beds and admitted at least 300 patients per year with a length of stay of 48 hours or more. Participants were family members of critically ill patients with an expected length of ICU stay of 48 hours or more and had (1) a life-threatening condition with a high risk of death or of long-lasting functional impairment and/or (2) a high risk of prolonged mechanical ventilation (≥24 hours), as appraised by the admitting clinician. Family members were eligible if they where at least 18 years old, a primary support person, and cognitively able to participate in German.

### Randomization and Masking

After the baseline assessment, ICU clusters were assigned 1:1 to an intervention arm or the control arm using minimization. The minimized cluster characteristics were certification of the ICU (major vs other teaching hospitals) and hospital (2 had >1 ICU).^33^ ICU and research staff were not masked. Participants may or may not have been aware of study assignment.

### Intervention and Control

ICUs allocated to the intervention arm introduced the family support intervention in addition to usual family care,^31^ using a tailored implementation strategy.^37^ The intervention, described in detail in the study protocol,^31^ is grounded in a family systems approach^38,39,40^ and evidence-based recommendations^14,16^ for family-focused ICU care. It consists of a family care pathway with 3 intervention components (engaging and liaising, supporting, and communicating), offered through regular interactions with families, from patient ICU admission into the post-ICU phase (eFigure 1 in Supplement 2). Intervention delivery had to start within 4 days of admission and include at least 1 follow-up after ICU discharge.^31^ A minimal dose of 5 interventions reflecting all 3 components was specified along the family care pathway, but the dose and/or frequency could be increased according to families’ needs and patients’ clinical condition.

To deliver the intervention, the novel role of family nurses within ICU teams was created. Per each ICU, 2 to 3 family nurses were responsible for intervention delivery, for which they had blocked time windows. Their role included coordinating ICU family care and ensuring continuity, meeting regularly with families to assess their needs, offering necessary support through relationship-focused and psychoeducational interventions, and facilitating interprofessional communication and shared decision-making with family members.

ICU family nurses received a 5-day training in family systems care and the protocolized intervention by the research team, which focused on preparing them for their role and the outlined intervention activities. Monthly online case conferences and 2 on-site refresher days were held to enable mutual learning. External implementation supporters met regularly with the core implementation team on each ICU to discuss progress and support implementation.^37,41^

ICUs in the control arm continued with their standard care to families, which was defined as a nonprotocolized approach that was an established part of ICU care before the trial started.

### Data Collection Procedures

Baseline ICU characteristics were collected through the routine ICU dataset. Family care processes were assessed through a structured self-report questionnaire prior to randomization by a core group of 3 to 5 ICU nurses and physicians. Cluster retention was ensured through quarterly study group meetings, newsletters, and regular site visits.

Family members were consecutively screened and invited by clinicians or local research staff within 96 hours after ICU admission. Participants signed a written informed consent form and completed a baseline questionnaire, which obtained information on their demographics and health status before starting the intervention. Patients’ demographic and medical data at ICU admission and discharge were extracted from the hospitals’ clinical records if patients had given general consent for the use of their clinical data for research or for this trial or if their family member had provided surrogate informed consent in case of incapacitation. Intervention delivery was recorded using structured logs.

For follow-up data assessment, the research staff sent a personalized link to an online questionnaire implemented in the REDCap electronic data capture tool or distributed a paper-and-pencil questionnaire, depending participants’ preferences. Up to 3 reminders were issued, including 1 by phone, and by email or letters.

### Outcomes

Outcomes were measured once at ICU patient discharge (1 day before to 90 days after discharge). The primary outcome was family satisfaction with ICU care, assessed with the 26-item Family Satisfaction with the ICU revised scale.^42^ This scale measures overall family satisfaction with ICU care and has 2 subscales (treated as secondary outcomes): satisfaction with care (16-item survey) and satisfaction with involvement in decision-making (10-item survey). The total score and subscale scores range from 0 to 100, with a score of 100 indicating high satisfaction. Cronbach α coefficients in the current sample were 0.93, 0.89, and 0.88 for the overall scale and the subscales, respectively.

The secondary outcomes were the quality of family-clinician communication and family perception of cognitive and emotional support that nurses provided to the family unit, which were assessed using the 14-item Questionnaire on Quality of Physician-Patient Interaction^43^ and the 14-item Family Perceived Support questionnaire.^44^ The Questionnaire on Quality of Physician-Patient Interaction has a mean score range of 1 to 5, with a score of 5 indicating high-quality communication. The Family Perceived Support Questionnaire score ranges from 14 to 70, with a score of 70 indicating high-perceived support. The Cronbach α coefficient was 0.95 for both scales in our sample.

### Statistical Analysis

We assumed an intervention effect of 5.5 points and a within-group SD of 16.3 based on our pilot-feasibility study,^32^ an intraclass correlation coefficient (ICC) of 0.03, and a coefficient of variation in cluster size of 0.2. To ensure 80% power at a 2-sided significance level of 5%, 8 clusters with an average of 50 evaluable participants are required. To account for a 10% dropout rate, we planned to recruit 56 participants per cluster (896 in total).^31^

The main analysis of the primary outcome was done using a linear mixed-effects model with a random intercept per cluster and the intervention (vs control) as the only explanatory variable. The Satterthwaite approximation for the denominator degrees of freedom was used. We performed several sensitivity analyses for the primary outcome, adjusting either for the baseline ICU certification, nurse staffing, or family-centered care score (cluster characteristics) and/or for several patient and family member characteristics. Two sensitivity analyses to account for the time to the return of the questionnaire were performed post hoc. All analyses were conducted using complete cases, with some of them, including the main model, also performed using multiple imputations of missing values. The ICC for the primary outcome was estimated from several models. The secondary outcomes were analyzed as described for the main analysis of the primary outcome.

Subgroup analyses of the primary outcome and one secondary outcome (quality of family-clinician communication) were performed for the cluster characteristics and several baseline patient and family member characteristics. Additional exploratory analyses were conducted for the intervention fidelity indicators. The prespecified analyses have been detailed in the statistical analysis plan (Supplement 1).^33^ Statistical analyses were performed using R version 4.5.0 and Stata version 18 (Stata Corp LLC; data preparation and descriptive analyses of cluster data).

## Results

### Participants

Of the 33 ICUs assessed, 16 agreed to participate and were randomized (Figure). Between December 5, 2022, and February 1, 2024, a total of 3181 family members were screened. Of 2075 invited, a total of 885 family members (42.7%) were included: 412 in the intervention arm and 473 in the control arm. The first follow-up was completed by 805 participants (91.0%), with a median time to questionnaire return of 5.0 days (IQR, 2.0-11.0; eFigure 2 in Supplement 2).

**Figure.**
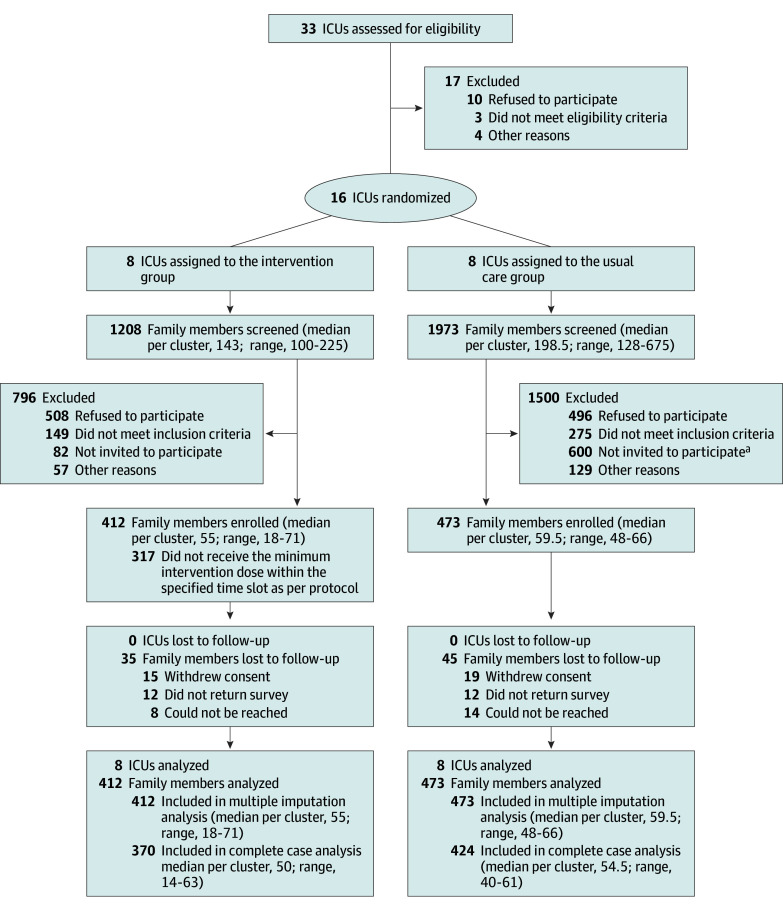
Participant Flow Through the FICUS Trial ^a^One intensive care unit (ICU) alone, which was the largest one in the trial, did not reach 484 eligible family members.

ICU characteristics were similar between study arms at baseline (Table 1),^45,46,47,48^ although the control arm included 1 more major teaching hospital. ICUs in the control arm had slightly more beds and larger numbers of patients admitted, and they had a larger proportion of patients requiring mechanical ventilation.

**Table 1.** Baseline ICU Characteristics and Family Care Processes

	Arm	
	Control (n = 8)	Intervention (n = 8)
**ICU characteristic**		
Certification, No. (%)^a^		
Major teaching hospitals	6 (75.0)	5 (62.5)
Other teaching hospitals	2 (25.0)	3 (37.5)
Operated ICU beds, median (IQR)^b^	15.4 (9.5-36.1)	11.6 (8.0-20.0)
Patients admitted per year, median (IQR)	1145.5 (533-3718)	918.5 (701-2203)
Treatment d/y, median (IQR)	4076.0 (2543-13 177)	3828.3 (2233-5531)
High-risk admissions (SAPS II score >45%), median (IQR)	24.1 (14.0-46.0)	21.0 (14.5-28.0)
Unplanned admissions, median (IQR), %	77.0 (59.0-94.2)	77.0 (43.9-91.0)
Category of SSICM classification of treatment shifts, median (range), %^c^		
1a	33.0 (23.0-58.8)	33.5 (15.0-62.1)
1b	30.0 (24.7-37.0)	31.1 (23.3-39.0)
2	29.6 (15.3-42.0)	32.4 (14.2-44.0)
3	3.0 (0.8-5.0)	4.0 (0.4-9.0)
Primary diagnosis at admission, median (IQR), %		
Cardiac	24.3 (11.7-40.0)	35.7 (1.1-88.6)
Respiratory	17.5 (11.0-26.6)	15.4 (1.8-33.1)
Gastrointestinal	14.1 (4.5-19.5)	14.4 (0.6-18.0)
Neurological	13.6 (8.7-23.2)	10.8 (1.9-90.3)
Metabolic-endocrine	4.4 (1.7-12.7)	3.8 (1.1-12.0)
Trauma	7.6 (2.4-30.3)	2.1 (0.0-11.0)
Urogenital	0.6 (0.0-2.9)	0.3 (0.0-4.0)
Other	12.2 (9.0-18.4)	7.6 (2.5-12.0)
Mechanically ventilated, median (IQR), %	44.0 (20.0-65.1)	35.5 (25.0-83.3)
>95 h of mechanical ventilation, median (IQR), %	7.8 (5.8-14.0)	7.8 (5.0-25.0)
>1000 NEMS points, median (IQR), %	5.0 (4.0-19.5)	6.6 (2.0-12.2)
Length of ICU stay, median (IQR), d	3.4 (2.3-6.5)	3.7 (1.2-4.9)
Discharge destination, median (IQR), %		
Other ICU	11.5 (3.0-36.0)	7.8 (2.0-35.1)
Intermediate care	72.5 (48.3-91.0)	76.3 (59.0-92.0)
General ward	6.5 (5.0-19.5)	6.5 (4.0-9.5)
Died	3.6 (1.0-14.0)	2.9 (0.0-13.0)
Other (rehabilitation, other institution, home)	11.5 (3.0-36.0)	7.8 (2.0-35.1)
FTE nurses per operated beds, median (IQR), %^a^	4.5 (3.8-5.4)	4.5 (2.8-7.1)
ICU certification, median (IQR), %		
Nurses	69.6 (54.5-80.5)	71.0 (48.5-82.9)
Physicians	38.0 (15.3-50.6)	34.7 (21.5-86.4)
**Family care processes**		
Family-centered care in ICU, median (IQR)^a^^,^^d^	3.1 (2.1-3.4)	2.9 (2.8-3.2)
Patient and family-centered care, median (IQR),^e^	2.1 (1.9-2.8)	2.1 (1.9-2.7)
Visitation policy		
24-h Access for family, No. (%)	2 (25.0)	1 (12.5)
Duration of daily visitation time, median (IQR), h	7.0 (6.0-9.0)	8.0 (6.0-9.0)
No. of family members with parallel access, median (IQR)	2.0 (2.0-no limit)	2.0 (2.0-2.0)
Information availability for family, No. (%)		
Brochure	8 (100)	8 (100)
Website	8 (100)	7 (87.5)
Oral information provided to family	8 (100)	8 (100)
Family support structure, No. (%)		
Proactive, daily call to family	7 (87.5)	5 (62.5)
Family diary	2 (25.0)	0
Hospital support structure, No. (%)		
Social work	8 (100)	6 (75.0)
Chaplain or spiritual care	8 (100)	8 (100)
Psychological counselling	6 (75.0)	4 (50.0)

Abbreviations: FTE, full-time equivalent; ICU, intensive care unit; NEMS, Nine Equivalents of Nursing Manpower Use Score; SAPS II score, Simplified Acute Physiology Score II; SSICM, Swiss Society for Intensive Care Medicine.

^a^ Variables used for sensitivity and subgroup analyses.

^b^ Annual average.

^c^ SSICM patient categorization system based on NEMS^45^ and the Sedation Agitation Scale–Richmond Agitation-Sedation Scale^46^ (category 1 indicates the highest care need).

^d^ Mean score (range, 1-4; 1 indicates the highest degree of family-centeredness) based on 22 items selected from the gap analysis tool provided by the SSICM.^47^

^e^ Mean score (range, 1-5) of 30 selected items from the Patient- and Family-Centered Care Organizational Self-Assessment Tool.^48^

The baseline family member characteristics were well-balanced between study arms (Table 2). Patient characteristics were similar except for unplanned admissions and trauma treatment in case of surgery, which were more frequent in the control arm, and mechanical ventilation, which was more frequent in the intervention arm (Table 2). Although illness severity (Simplified Acute Physiology Score II [SAPS II]) was similar at baseline, slightly more patients whose families were in the intervention arm had died in the ICU than patients whose families were in the control arm.

**Table 2.** Baseline Characteristics of Patients and Family Members

	Arm		Missing, %
	Control (n = 473)	Intervention (n = 412)	
**Patient characteristics**			
Age, median (IQR), y^a^	66.0 (55.0-75.0)	67.5 (56.0-75.0)	2.3
Sex, No. (%)^b^			
Female	154 (33.1)	159 (39.7)	2.1
Male	311 (66.9)	241 (60.1)	
Other	0	1 (0.2)	
Civil status, No. (%)			
Single	79 (17.0)	47 (11.7)	2.1
Married or in (registered) partnership	294 (63.2)	291 (72.6)	
Divorced or separated	54 (11.6)	37 (9.2)	
Widowed or surviving partner	38 (8.2)	26 (6.5)	
Unplanned admission to ICU, No. (%)^a^	417 (89.9)	309 (77.1)	2.3
Admitted from, No. (%)			
Emergency department	185 (39.8)	137 (34.2)	2.1
Operating room	135 (29.0)	125 (31.2)	
General ward	69 (14.8)	66 (16.5)	
Intermediate care	31 (6.7)	25 (6.2)	
Other ICU	44 (9.5)	48 (12.6)	
Other institution (rehabilitation, nursing home)	1 (0.2)	0	
Mechanical ventilation, No. (%)	230 (49.5)	242 (60.3)	2.1
Mechanical circulatory support, No. (%)	24 (5.2)	34 (8.5)	2.1
SAPS II score, median (IQR)^a^	51.0 (18.6)	50.5 (17.3)	2.8
NEMS score, median (IQR)	34.0 (27.0-39.0)	34.0 (27.0-39.0)	2.1
SOFA score, median (IQR)	8.5 (6.0-11.0)	8.0 (6.0-11.0)	2.3
Surgery, No. (%)			
Planned	31 (6.7)	68 (17.0)	2.1
Emergency	138 (29.7)	97 (24.2)	
None	296 (63.7)	236 (58.9)	
Trauma treatment in case of surgery, No. (%)^c^	44 (26.0)^c^	7 (4.2)^c^	62.3
AIS score in case of trauma treatment, median (IQR)^d^	29.0 (25.0-37.0)^d^	25.0 (25.0-37.0)^d^	94.4
Previous ICU-treatment within last 3 mo, No. (%)	83 (17.8)	66 (16.5)	2.1
Length of ICU stay, median (IQR), d^e^	8.0 (5.0-13.0)	10.0 (6.0-16.0)	3.2
Died in ICU, No. (%)^e^	61 (13.2)	79 (19.9)	3.2
**Family characteristic**			
Age, median (IQR), y	55.0 (42.0-63.8)	54.0 (43.0-66.0)	4.1
Sex (self-reported), No. (%)			
Female	314 (69.6)	254 (63.7)	4.0
Male	136 (30.2)	145 (36.3)	
Other	1 (0.2)	0	
Civil status, No. (%)			
Single	36 (8.0)	38 (9.6)	4.6
Married in registered partnership	363 (81.0)	322 (81.3)	
Divorced or separated	31 (6.9)	26 (6.6)	
Widowed or surviving partner	18 (4.0)	10 (2.5)	
Occupational status, No. (%)			
Employed full- or part-time	284 (63.3)	255 (63.9)	4.2
Retired	111 (24.7)	112 (28.1)	
Student	10 (2.2)	7 (1.8)	
Unemployed	44 (9.8)	25 (6.3)	
Type of family member, No. (%)^a^			
Partner or spouse	215 (47.7)	214 (53.6)	4.0
Parent	65 (14.4)	59 (14.8)	
Child	113 (25.1)	98 (24.6)	
Other	58 (12.9)	28 (7.0)	
Cohabiting with patient, No. (%)	252 (55.9)	255 (63.9)	4.0
Travel time to hospital, median (IQR), min	30.0 (20.0-60.0)	37.5 (20.0-60.0)	5.5
Self-perceived health by 0-100 VAS, median (IQR)	80.0 (70.0-90.0)	80.0 (70.0-90.0)	4.5
Past or current psychiatric or psychological treatment, No. (%)	175 (39.0)	148 (37.1)	4.3
Current use of prescription drugs, No. (%)	188 (42.0)	173 (43.4)	4.3
Current treatment for chronic illness, No. (%)	96 (21.5)	89 (22.5)	4.7
Prior ICU experience, No. (%)^a^			
Patient	36 (8.0)	19 (4.8)	4.3
Family member of patient	168 (37.4)	141 (35.4)	
Both	23 (5.1)	21 (5.3)	
None	222 (49.4)	217 (54.5)	
Anxiety, mean (SD)^b^^,^^f^	8.8 (4.4)	9.5 (4.4)	24.3
Depression, mean (SD)^b^^,^^f^	6.5 (4.6)	7.0 (4.7)	24.5
Family resilience, mean (SD)^b^^,^^g^	3.5 (0.7)	3.5 (0.7)	24.4
Family functioning, mean (SD)^b^^,^^h^	1.6 (0.5)	1.6 (0.4)	24.5

Abbreviations: AIS, abbreviated injury scale; ICU, intensive care unit; NEMS, Nine Equivalents of Nursing Manpower Use; SAPS II, Simplified Acute Physiology II; SOFA, Sequential Organ Failure Assessment; VAS, visual analog scale.

^a^ Variables used for sensitivity and subgroup analyses.

^b^ Variables used for subgroup analyses only.

^c^ Of those with surgery (planned or emergency; n = 169 in control; n = 165 in intervention).

^d^ Of those with trauma treatment and AIS score (n = 43 in control; n = 7 in intervention).

^e^ These are not baseline patient characteristics. They were assessed at patient discharge.

^f^ Measured by the Hospital Anxiety and Depression scale (score range, 1 to 21; high scores indicates anxiety or depression).

^g^ Measured by the Brief Resilience Scale (score range, 1 to 5; high score indicates resilience).

^h^ Measured by the Family Assessment Device-General Functioning 12 scale (score range, 1 to 4; low score indicates functioning).

### Intervention Fidelity

The intervention details and fidelity indicators are shown in Table 3 and eTables 1 and 2 in Supplement 2. A total of 22 certified ICU nurses with a median of 19 years of ICU experience (IQR, 2.5-29.0 years), already employed in the ICU (n = 18) or in another service at the hospital (n = 5), delivered the intervention, with 22.2% of intervention sessions delivered together with ICU physician and nurses. Fidelity to each intervention component per time point ranged from 43.9% to 90.8% (Table 3), with only 23.1% (n = 95) of families receiving all intervention components within the prespecified time points along the family care pathway.

**Table 3.** Intervention Delivery and Fidelity Dimensions

Intervention delivery and fidelity dimensions	Intervention arm (n = 412)	Missing, %
Intervention contacts per family, median (IQR)		
Entire care pathway	11.0 (8.0-15.0)	0
During ICU	7.0 (5.0-11.0)	0
Post-ICU	3.0 (2.0-4.5)	0
Intervention contacts per family by type of intervention (core component), median (IQR)		
Encounters (engaging and liaising)	2.0 (2.0-4.0)	0
Supporting		
Therapeutic conversations at admission^a^	1.0 (1.0-2.0)	0
Therapeutic conversations during ICU stay^b^	1.0 (1.0-3.0)	0
Post-ICU therapeutic conversations	2.0 (1.0-3.0)	0
Interprofessional family meetings (communicating)	2.0 (1.0-3.0)	0
Duration of intervention contacts per family, median (IQR), min		
Entire care pathway	250.0 (160.0-375.0)	0
During ICU	175.0 (110.0-282.5)	0
Post-ICU	60.0 (30.0-102.5)	0
Duration per single contact by type of intervention or core component, median (IQR), min		
Encounters (engaging and liaising)	10.0 (5.0-15.0)	0
Supporting		
Therapeutic conversations at admission^a^	35.0 (30.0-50.0)	0
Therapeutic conversations during ICU stay^b^	30.0 (20.0-40.0)	0
Post-ICU therapeutic conversations	25.0 (15.0-30.0)	0
Interprofessional family meetings (communicating)	30.0 (20.0-40.0)	0
Fidelity dimensions interventions		
Consistency, No. (%)^c^^,^^d^	95 (23.1)	0
Dose in min per patient ICU LOS, median (IQR)^d^^,^^e^	25.0 (16.6-35.3)	0
Contacts per patient ICU LOS, median (IQR)^d^^,^^f^	1.1 (0.8-1.5)	0
Intervention component along family care pathway, No. (%)		
Encounters (engaging and liaising) at admission to ICU ≤96 h	374 (90.8)	3.9
Supporting		
Therapeutic conversations at admission to ICU ≤96 h	363 (88.1)	4.6
Therapeutic conversations at discharge from ICU ≥48 h	290 (70.4)	14.1
Post-ICU phase within 4 wk	181 (43.9)	11.7
Interprofessional family meetings (communicating) at admission to ICU ≤96 h	301 (73.1)	12.1
Any type of intervention post-ICU	379 (92.0)	1.9

Abbreviations: ICU, intensive care unit; LOS, length of stay.

^a^ Nurse-family conversations no later than 4 days after ICU admission and 1 day prior to ICU discharge (n = 399 because for 13 cases, the date of ICU discharge or death was unavailable).

^b^ All later nurse-family conversations during ICU stay (n = 399).

^c^ Fidelity consistency indicates fidelity to intervention content, defined as minimal intervention contact dose according to the protocol was provided within the specified timeframe (ie, 5 contact doses, representing all 3 intervention components within specified timeframe, see eFigure 1 in Supplement 2).

^d^ Variables used in additional analyses.

^e^ Fidelity dose indicates the total duration of interventions (conversations) in minutes divided by patient length of ICU stay (n = 399).

^f^ Fidelity frequency represents the total number of interventions (conversations) divided by patient length of ICU stay (n = 399).

### Primary Outcome

Overall, the mean (SD) family satisfaction score with ICU care was higher in the intervention arm (81.78 [14.64]) than in the control arm (79.39 [15.12]). In the main analysis using complete cases, the mean difference (MD) was estimated as 2.39 (95% CI, 0.31-4.47; *P* = .02) and using multiple imputations as 2.44 (95% CI, 0.31-4.57; *P* = .02), with details given in Table 4. Compared with the sample size calculation, the estimated intervention effect (MD, 2.39 vs 5.5) and SD (14.9 vs 16.3) were smaller, whereas the ICC was estimated at nearly 0 instead of 0.03 (Table 4 and eTables 19 and 20 in Supplement 2). However, the intervention effect remained fairly stable in all sensitivity analyses, which estimated similar intervention effects (MD, 2.34-2.79; eTables 3 through 11 in Supplement 2). Sensitivity analyses using multiple imputations and adjusting only for cluster characteristics provided moderate evidence against the null hypothesis (between *P*< .01 and *P* < .05; eTables 3-5 and 10-11 in Supplement 2).^49^ When adjusting for patient and family member characteristics without multiple imputation, we found only weak evidence against the null hypothesis (between *P* < .05 and *P*< .01; eTables 6-9 in Supplement 2) despite larger estimated intervention effects, likely due to reduced sample sizes because of missing covariate data. The results were robust regarding timely return of questionnaires (≤14 days; eTables 12-13 in Supplement 2). Higher SAPS II scores were associated with higher family satisfaction, which was the only covariate that was significantly associated with the primary outcome (eTables 6-9 and 11 in Supplement 2).

**Table 4.** Primary and Secondary Outcomes^a^

Outcomes	Mean (SD)		Coefficient estimate (95% CI)	*P* value	Missing, %
	Control (n = 473)	Intervention (n = 412)			
**Primary outcome**					
Family satisfaction with ICU care^b^	79.39 (15.12)	81.78 (14.64)	2.39 (0.31 to 4.47)	.02	10.3
Applied to multiply imputed dataset^c^			2.44 (0.31 to 4.57)	.03	0
**Secondary outcomes**					
Satisfaction with care^d^	80.94 (16.62)	83.20 (16.34)	2.26 (−0.26 to 4.79)	.08	11.0
Satisfaction with decision-making involvement^e^	77.07 (15.90)	79.91 (14.51)	2.84 (0.71 to 4.98)	.009	10.4
Family-clinician communication^f^	3.45 (0.91)	3.82 (0.80)	0.37 (0.16 to 0.58)	.002	12.1
Nurse cognitive and emotional family support^g^	39.33 (15.84)	48.36 (13.77)	8.71 (4.71 to 12.71)	<.001	12.5

^a^ Intraclass correlation coefficients (ICCs) for the primary and secondary outcomes are reported in the eTable 17 in Supplement 2. Distribution of the primary outcome and diagnostic plots of the residuals are reported in eFigures 5-7 in Supplement 2.

^b^ Derived from the 26-item Family Satisfaction with ICU–revised version questionnaire (German version; score range, 0-100; high scores indicate high satisfaction).

^c^ Same model as for main analysis, but with multiple imputations of missing outcome data, see eTable 10 in Supplement 2 (control, n = 473; intervention, n = 412; fraction of missing information,  = 0.12).

^d^ Derived from the 16-item subscale of the Family Satisfaction with ICU-revised version questionnaire (German version; range, 0-100; high scores indicate high satisfaction).

^e^ Derived from the 10-item subscale of the Family Satisfaction with ICU-revised version questionnaire (German version; subscale score range, 0-100; high scores indicate high satisfaction).

^f^ Derived from the 14-item Questionnaire on the Quality of Physician-Patient Interaction German version; score range, 1-5; high scores indicate better quality).

^g^ Derived from the 14-item Family Perceived Support Questionnaire (German version; score range, 14-70; high scores represent better support.

Prespecified subgroup analyses revealed no significant differences in the intervention effect, with one exception (eFigures 3-4 in Supplement 2): The type of ICU admission was shown to modify the intervention effect, which was larger in case of a planned ICU admission (MD, 8.67; 95% CI, 3.13 to 14.20; *P* = .002) and decreased by 6.82 points (95% CI, −12.80 to −0.83; *P* = .03) with an unplanned admission (eTable 14 in Supplement 2). It should be noted that most patients had an unplanned ICU admission (control, 417 [89.9%]; intervention, 309 [77.1%]; Table 2), with an intervention effect slightly smaller than overall.

### Secondary Outcomes

A strong intervention effect was noted on satisfaction with involvement in decision-making (MD, 2.84; 95% CI, 0.71 to 4.98; *P* = .009), and a weaker effect on satisfaction with care (MD, 2.26; 95% CI, −0.26 to 4.79; *P* = .08; Table 4). The intervention clearly improved the quality of family-clinician communication (MD, 0.37; 95% CI, 0.16 to 0.58; *P* = .002) and the perceived cognitive and emotional nurse support (MD, 8.71; 95% CI, 4.71 to 12.71; *P* < .001) compared with the control arm. A subgroup analysis of the family-clinician communication is reported in eTable 15 in Supplement 2.

### Additional Analyses

Fidelity consistency, but not the dose or frequency, was associated with improvements regarding satisfaction with ICU care, family-clinician communication, and nurse support. For satisfaction, receiving the intervention consistent with the protocolized family care pathway, thereby combining all components within the prespecified timeframes, resulted in a stronger intervention effect (MD, 3.87; 95% CI, 0.29 to 7.45; *P* = .03) than receiving an inconsistently delivered intervention (MD, 2.30; 95% CI, −0.21 to 4.82; *P* = .07; eTable 16 in Supplement 2). For communication and support, both consistent and inconsistent delivery showed a significant intervention effect (eTables 17-18 in Supplement 2).

## Discussion

The nurse-led, multicomponent family support intervention for families of critically ill adults slightly improved family satisfaction with ICU care with a clinically uncertain benefit. Satisfaction was high in ICUs of both study arms, as previously observed,^4,44,50^ and the magnitude of the intervention effect was small. A strong increase was found for quality of communication and cognitive and emotional support provided to families. Here, the intervention effect exceeded the minimal clinically important difference according to the distribution-based standard error of the measurement method (eTable 19 in Supplement 2).

Similar to FICUS, the Pairing Re-engineered ICU Teams with Nurse-Driven Emotional Support and Relationship-Building (PARTNER)^26^ trial, carried out in 4 US ICUs, had identified improved quality of communication and person-centeredness in surrogates.^26^ The family support intervention differs from the PARTNER intervention because it also uses relationship-focused and psychoeducational interventions, which are grounded in a family systems approach, and follows up families into the post-ICU phase. Nevertheless, both trials suggest that nurse-led family care pathways combining interprofessional family communication structures with regular nurse check-ins with families may be a promising approach to increase quality of family care in the ICU.

The family support intervention introduces staff capacity by creating a designated role of an ICU family nurse who is part of the interprofessional ICU team and enables care to families as recommended by professional societies.^15,16^ Over one-fifth of interventions were offered by family nurses together with ICU physicians and/or nurses, which underscores the interprofessional nature of this interventional approach and the added value of a dedicated ICU family nurse. Fidelity to the minimal dose with its prespecified timeline and intervention component configuration was low, but fidelity to each intervention component per time point can be considered moderate to high,^51^ indicating that the family support intervention is a feasible intervention. Lack of fidelity occurred mainly due to deviations from the prespecified timeline, particularly after discharge from the ICU. Hence, in addition to standardization, some flexibility may be required to tailor the intervention to specific family situations and ICU treatment processes.^51^

For family satisfaction, but not for secondary outcomes, absence of consistent delivery reduced the magnitude of the intervention effect, as previously noted.^52^ Our findings suggest that the combination of all 3 intervention components may be more important for achieving family satisfaction than the actual intervention dose or frequency. However, further research is needed to identify the active ingredients or core functions of the intervention to which adherence is required.^51^

The need for family interventions to target modifiable risk factors for postintensive care syndrome–family, such as communication quality and satisfaction, has been increasingly claimed.^13,27,53^ Satisfaction and communication quality are potential mediators for developing postintensive care syndrome–family^12,13,53^ and were therefore used as the proximal outcomes in our trial. The ability of the family support intervention to achieve impact on family functioning, well-being, and psychological sequelae, either directly or mediated by our quality of care indicators in a cost-effective manner, is still under investigation.^31^ To date, nurse-facilitated or nurse-aided interventions have failed to identify marked improvements on post-ICU family health^25,26,27^ with the exception of reduced depression at 6 months^25^ and on prolonged and severity of grief.^28^ Systematic reviews and meta-analyses identified beneficial impact of family-focused interventions in the ICU,^19,20,21,22,23^ but the evidence base remains contradictory, which may be due to heterogeneity in family interventions and populations analyzed in these reviews.

In this trial, mortality risk, as assessed with the SAPS II score, was associated with higher family satisfaction, but the implication of these findings remains unclear. Satisfaction ratings in family members of nonsurvivors have been reported to be higher than in survivors,^50^ whereas another study did not find an association between patient disease severity and family satisfaction.^54^ This intervention targeted family members of critically ill patients with sudden or unexpected critical illness, which was the case for the majority of participants. The stronger intervention effect in case of a planned admission is difficult to interpret. It is possible that a planned admission with subsequent critical illness may be particularly distressing for families and may result in high support needs.

### Limitations

This trial has several limitations. First, family members were recruited after cluster randomization, with nonblinded recruiters.^55,56^ Second, it may be possible that those most in need of support and communication could not be recruited due to high burden faced by the unexpected and acute patient situation, which is a common challenge.^57^ Enrollment of almost half of invited family members is therefore notable. Retention at the first follow-up was more than 90% and comparable between study arm, which is satisfactory given the high burden family members bear during critical illness.^58^ Third, some ICU family care processes and actual patient characteristics differed slightly between study arms at baseline, namely type of admission, trauma treatment, and proportion of patients receiving mechanical ventilation but not illness severity (SAPS II). Some of these differences may be because the control arm had a higher number of larger ICUs than the intervention arm and need to be considered when interpreting the findings. Fourth, within intervention ICUs, adherence to interventions along the care pathway was high, but only one quarter of participants received the intervention exactly according to the timeline specified in the protocol. This was most likely due to study-related requirements, like consent process and baseline data collection, which needed to be completed before starting intervention delivery. In addition, a spillover effect may have occurred to control ICUs, thereby potentially reducing the magnitude of the intervention effect.^59^ Fifth, the high level of family satisfaction in both study arms was higher than assumed in our sample size calculation,^31^ which led to a ceiling effect in the measurement of the main outcome and to a violation of the normality assumption for the linear mixed-effects model. Because the ceiling effect was stronger in the intervention arm than in the control arm, it may have led to an underestimation of the intervention effect of the primary outcome, which did not achieve a minimal clinically important difference. Despite these limitations, this pragmatic, rigorous trial was grounded in the real-world contexts of Swiss adult ICUs of different specialty, size, and geographical location. Research on the reproducibility and scalability of nurse-facilitated, multicomponent family support interventions to other cultural contexts and health care systems is needed and should investigate care outcomes, health benefit, and implementation success.

## Conclusions

In conclusion, this nurse-led, intense, interprofessional family support intervention in the ICU had a small, statistically significant, but clinically uncertain benefit on family satisfaction, while improving quality of communication and emotional and cognitive nurse support. Families, clinicians, and policymakers may use these findings to build capacity and promote family-focused ICU care. Validation of these results and further study of the clinical benefit of the intervention are needed across populations and language regions. Nevertheless, incorporating this intervention as a standard component of ICU care may be of value.

## Notes

Supplement 1.
Trial protocol

Supplement 2. **eTable 1.** Characteristics of intervention contacts **eTable 2.** Characteristics of intervention conacts with referrals **eTable 3.** Coefficient estimates from the sensitivity analysis (model 2) **eTable 4.** Coefficient estimates from the sensitivity analysis (model 3) **eTable 5.** Coefficient estimates from the sensitivity analysis (model 4) **eTable 6.** Coefficient estimates from the sensitivity analysis (model 5) **eTable 7.** Coefficient estimates from the sensitivity analysis (model 6) **eTable 8.** Coefficient estimates from the sensitivity analysis (model 7) **eTable 9.** Coefficient estimates from the sensitivity analysis (model 8) **eTable 10.** Coefficient estimates from the sensitivity analysis (model 9) **eTable 11.** Coefficient estimates from the sensitivity analysis (model 10) **eTable 12.** Coefficient estimates from the post hoc sensitivity analysis on excluding participants QoC questionnaire return later than 14 days after T1 **eTable 13.** Effect size estimate for the effect of the Intervention vs. Control on the primary outcome **eTable 14.** Coefficient estimates for the subgroup analysis on the primary outcome **eTable 15.** Coefficient estimates for the subgroup analysis on the secondary outcome *QQPPI* with the family-centered care **eTable 16.** Coefficient estimates from the additional analysis of the primary outcome **eTable 17.** Coefficient estimates from the additional analysis of the secondary outcome *quality of communication* **eTable 18.** Coefficient estimates from the additional analysis of the secondary outcome *nurse support* **eTable 19.** Minimal Clinically Important Difference (MCID) for all outcomes **eTable 20.** Intraclass correlation coefficients (ICC) for the primary outcome **eFigure 1.** Family support intervention **eFigure 2.** Distribution of the time to return of the QoC survey questionnaires **eFigure 3.** Forest plot of treatment effect estimates on the primary outcome **eFigure 4.** Forest plot of treatment effect estimates on the primary outcome **eFigure 5.** Diagnostic plots for the residuals from model 1 applied to the primary outcome, **eFigure 6.** Diagnostic plots for the residuals from model 5 applied to the primary outcome **eFigure 7.** Violin plots showing the distribution of the primary outcome by treatment
eReferences

Supplement 3.
Ficus Study Group

Supplement 4.
Data Sharing Statement
