# Supplementary material for: Nurse-Led Family Support Intervention for Families of Critically Ill Patients: The FICUS Cluster Randomized Clinical Trial
Source: JAMA Intern Med. 2025 Jul 28;185(9):1138–49. doi: 10.1001/jamainternmed.2025.3406 (PMC12558129; doi:10.1001/jamainternmed.2025.3406)
Supplement: Supplement 1. — Trial protocol [file jamainternmed-e253406-s001.pdf]

Naef et al. Trials (2022) 23:533
https://doi.org/10.1186/s13063-022-06454-y

Trials

STUDY PROTOCOL

Open Access

A multicomponent family support intervention in intensive care units: study protocol for a multicenter cluster-randomized trial (FICUS Trial)

Rahel Naef[1,2*], Miodrag Filipovic[3], Marie-Madlen Jeitziner[4], Stefanie von Felten[5], Judith Safford[6], Marco Riguzzi[1,2] and Michael Rufer[7,8]

Abstract

Background: Family members of critically ill patients face considerable uncertainty and distress during their close others' intensive care unit (ICU) stay. About 20–60% of family members experience adverse mental health outcomes post-ICU, such as symptoms of anxiety, depression, and posttraumatic stress. Guidelines recommend structured family inclusion, communication, and support, but the existing evidence base around protocolized family support interventions is modest and requires substantiation.

Methods: To test the clinical effectiveness and explore the implementation of a multicomponent, nurse-led family support intervention in ICUs, we will undertake a parallel, cluster-randomized, controlled, multicenter superiority hybrid-type 1 trial. It will include eight clusters (ICUs) per study arm, with a projected total sample size of 896 family members of adult, critically ill patients treated in the German-speaking part of Switzerland. The trial targets family members of critically ill patients with an expected ICU stay of 48 h or longer. Families in the intervention arm will receive a family support intervention in addition to usual care. The intervention consists of specialist nurse support that is mapped to the patient pathway with follow-up care and includes psycho-educational and relationship-focused family interventions, and structured, interprofessional communication, and shared decision-making with families. Families in the control arm will receive usual care. The primary study endpoint is quality of family care, operationalized as family members' satisfaction with ICU care at discharge. Secondary endpoints include quality of communication and nurse support, family management of critical illness (functioning, resilience), and family members' mental health (well-being, psychological distress) measured at admission, discharge, and after 3, 6, and 12 months. Data of all participants, regardless of protocol adherence, will be analyzed using linear mixed-effects models, with the individual participant as the unit of inference.

Discussion: This trial will examine the effectiveness of the family support intervention and generate knowledge of its implementability. Both types of evidence are necessary to determine whether the intervention works as intended in clinical practice and could be scaled up to other ICUs. The study findings will make a significant contribution to the current body of knowledge on effective ICU care that promotes family participation and well-being.

*Correspondence: rahel.naef@uzh.ch

[1] Institute for Implementation Science in Health Care, Faculty of Medicine, University of Zurich, Universitätsstrasse 84, 8006 Zurich, Switzerland
Full list of author information is available at the end of the article

© The Author(s) 2022. **Open Access** This article is licensed under a Creative Commons Attribution 4.0 International License, which permits use, sharing, adaptation, distribution and reproduction in any medium or format, as long as you give appropriate credit to the original author(s) and the source, provide a link to the Creative Commons licence, and indicate if changes were made. The images or other third party material in this article are included in the article's Creative Commons licence, unless indicated otherwise in a credit line to the material. If material is not included in the article's Creative Commons licence and your intended use is not permitted by statutory regulation or exceeds the permitted use, you will need to obtain permission directly from the copyright holder. To view a copy of this licence, visit http://creativecommons.org/licenses/by/4.0/. The Creative Commons Public Domain Dedication waiver (http://creativecommons.org/publicdomain/zero/1.0/) applies to the data made available in this article, unless otherwise stated in a credit line to the data.

Trial registration: ClinicalTrials.gov NCT05280691. Prospectively registered on 20 February 2022.

Keywords: Intensive care (MeSH), Family (MeSH), Family nursing (MeSH), Anxiety (MeSH), Depression (MeSH), Post-traumatic stress disorder (MeSH), Postintensive care syndrome – family (non-MeSH), Satisfaction with care (non-MeSH), Cluster-randomized controlled trial (non-MeSH)

Administrative information

Title (1)	A multicomponent family support intervention in intensive care units: study protocol for a multicenter cluster-randomized trial (FICUS Trial)
Trial registration (2a and 2b).	ClinicalTrials.gov, NCT05280691, and the Swiss National Clinical Trials Portal (SNCTP), SNCTP000004842
Protocol version (3)	Version 1.0, 25. October 2021
Funding (4)	The study is funded by the Swiss National Science Fund (SNSF, grant no. 33IC30_198778/1).
Author details (5a)	**Rahel Naef:** Institute for Implementation Science in Health Care, Faculty of Medicine, University of Zurich and Center of Clinical Nursing Science, University Hospital Zurich, Universitätsstrasse 84, CH-8006 Zurich, Switzerland.<br>**Miodrag Filipovic:** Surgical Intensive Care Unit, Rescue and Pain Medicine, Intensive Care, Division of Anesthesiology, Cantonal Hospital of St. Gallen, Rorschacher Strasse 95, CH-9007 St. Gallen, Switzerland.<br>**Marie-Madlen Jeitziner:** Department of Intensive Care Medicine, University Hospital Bern, Inselspital, University of Bern, Freiburgstrasse 10, 3010 Bern, Switzerland.<br>**Stefanie von Felten:** Department of Biostatistics, Epidemiology, Biostatistics, and Prevention Institute, Faculty of Medicine, University of Zurich, Hirschengraben 84, 8001 Zurich, Switzerland.<br>**Judith Safford:** Patient representative, no affiliation<br>**Marco Riguzzi:** Institute for Implementation Science in Health Care, Faculty of Medicine, University of Zurich and Center of Clinical Nursing Science, University Hospital Zurich, Universitätsstrasse 84, 8006 Zurich, Switzerland.<br>**Michael Rufer:** Department of Psychiatry, Psychotherapy, and Psychosomatics, Psychiatric University Hospital Zurich, University of Zurich and Center for Psychiatry and Psychotherapy, Psychiatric Hospital Zugersee, Triaplus AG, Widenstrasse 55, 6317 Oberwil-Zug, Switzerland.
Name and contact information of the trial sponsor (5b)	Rahel Naef, PhD, RN<br>Institute for Implementation Science in Health Care, Faculty of Medicine, University of Zurich and Center of Clinical Nursing Science, University Hospital Zurich, Universitätsstrasse 84, CH-8006 Zurich, Switzerland.<br>E-Mail: rahel.naef@uzh.ch<br>Phone: +41 44 634 37 49
Role of sponsor (5c)	The sponsor-investigator acts as the coordinating investigator of the trial and has a primary role regarding study design; collection, management, analysis, and interpretation of the data; and writing of the report. She has ultimate authority over these activities.<br>The funder (Swiss National Science Fund) has no role in the development of the study design; collection, analysis, or interpretation of the data; writing of the manuscript; or decision to submit the manuscript for publication.

Introduction

Family members are important to the well-being and recovery of critically ill persons, yet they are themselves profoundly affected by the critical illness [1, 2]. During a close other's treatment in an intensive care unit (ICU), families experience high levels of stress and uncertainty [3], which often negatively affects their coping ability and mental health [4–6]. Research reports that 20–60% of family members experience subsequent adverse mental health outcomes, such as symptoms of anxiety, depression, posttraumatic stress, and complicated grief [7, 8], also known as post-intensive care syndrome – family (PICS-F) [9–11]. With the COVID-19 pandemic, incidences of PICS-F are likely to be even higher [12, 13].

The impact of critical illness on family members' health and well-being and the need to increase family access, inclusion, and support are gaining recognition [10, 14–16], even more so since the COVID-19 pandemic [13, 17–19]. A move towards structured inclusion, communication, and support of family is recommended [20]. While an abundance of research exists about how to better address families' needs during critical illness, empirical knowledge about the effectiveness and successful implementation of family interventions and models of care is only modest.

State of evidence on family interventions in ICU

Prior research has identified a promising effect of interventions seeking to increase family inclusion, communication, and support in ICU, such as participation in patient care or rounds, information/education, structured communication, or ICU diary, on quality of family care and family health outcomes [21–26]. While there is a general trend towards improved communication, decision-making, and family satisfaction and reports of reduced symptoms of anxiety, depression, and post-traumatic stress following such family interventions, effects were mostly found to be statistically non-significant with unclear clinical significance. The effects on length of ICU stay remain controversial [21, 22, 27], and most studies have used non-controlled designs [24, 25].

Studies that investigated specific, multicomponent, bundled family interventions, which included specific family support roles combined with facilitated communication, have demonstrated an increase in satisfaction with care and quality of communication [28–32], and have

established feasibility and acceptability of family liaison and navigator roles [28, 31, 33, 34], most recently in the context of the COVID-19 pandemic [35–37]. However, there is little evidence of the effectiveness of such interventions to reduce symptoms of anxiety, depression, or posttraumatic stress [30, 32, 34, 38, 39]. Only three randomized trials on family support interventions in ICU have been conducted to date. Curtis and colleagues [38], testing a communication facilitator intervention for family members of incapacitated patients involved in surrogate decision-making, found a decrease in depressive symptoms but not in anxiety 6 months post-ICU, and there was no statistically significant difference after 3 months. White and colleagues [30], who investigated a multicomponent, nurse-delivered family support intervention (FOUR SUPPORT) for the same target group, did not identify a clear trend. Just recently, Kentish-Barnes and colleagues [39], who implemented a physician-driven, nurse-aided support strategy for family members of patients dying in ICU following a decision to withdraw or withhold life support, found a significant reduction in the proportion of family members with prolonged grief symptoms and significantly lower grief scores in the intervention group.

Trial rationale

There is a clear clinical need for increased family communication and support during critical illness to improve evidence-based ICU care delivery [40, 41]. However, there is a lack of evidence-based family support programs. Our group has developed and pilot-feasibility tested a nurse-delivered family systems intervention program in a general ICU population using a mixed-method design. We were unable to demonstrate a favorable impact on post-ICU psychological distress, possibly due to the uncontrolled before-and-after comparison [32], but found statistically significant improved satisfaction levels. The qualitative evaluation showed self-perceived benefits for family management of critical illness and its aftermath when the intervention is initiated shortly after ICU admission, responsive to families' unique needs, and perceived to be delivered with high proficiency [32]. The pre-existing evidence base around multicomponent family support interventions is modest and requires further substantiation, particularly using randomized controlled designs [24–26]. Hence, the here proposed trial seeks to generate high-quality evidence on the intervention effectiveness of such multicomponent, bundled family support interventions in ICU while also generating knowledge around implementation processes and outcomes.

Objectives

The primary aim of the Family in Intensive Care UnitS (FICUS) trial is to test the effectiveness of a multicomponent, nurse-led family support intervention (FSI), on the quality of family care, family management of critical illness, and mental health of individual index family members of critically ill patients compared with usual ICU care (Fig. 1). We hypothesize that the FSI will (1) increase the quality of family care, i.e., satisfaction with ICU care (primary endpoint); (2) increase the quality of communication and nurse support as reported by family members after patient discharge/death; (3) improve family management of critical illness and individual well-being; and (4) decrease symptoms of psychological distress post-ICU. The secondary aim is to examine the implementation processes, that is, identify implementation barriers/enablers in the real-world context in which the study intervention is implemented to discern determinants and strategies of implementation success.

Design and methods

The FICUS trial is designed as a parallel, cluster-randomized, controlled, multicenter superiority hybrid type 1 trial with an equal number of clusters per study arm and a primary endpoint of quality of family care in ICU assessed after patient ICU discharge or death. We employ an effectiveness-implementation hybrid design type 1 [42], in which, alongside clinical effectiveness, contextual determinants (barriers/facilitators), together with

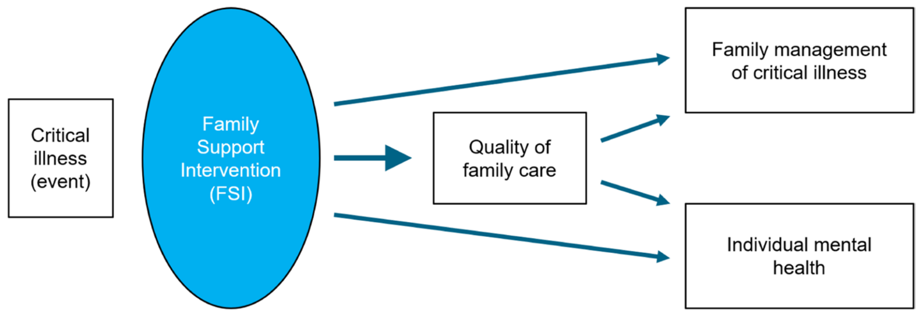

Fig. 1 Intervention mechanism

implementation processes and outcomes are explored. This protocol publication follows the SPIRIT guidance [43] (see the SPIRIT Checklist as an additional file to this manuscript).

Patient and public involvement

A patient and family advisory group (PFAG) with five members (one patient, three family members, one patient representative) ensures that the trial is relevant and meaningful to critically ill patients and their family members, contributes to the design of the study processes, and guarantees that participation is feasible and acceptable to family members [44–46]. An ongoing partnership has been formed between the research team and the PFAG, with one member acting as a Patient and Public Involvement (PPI) liaison. This person co-designed the involvement strategy, co-leads consultation and communication, and acts as a liaison between the research team and the PFAG. Furthermore, PFAG members give advice, contribute their expertise to the trial design, and are actively involved in trial implementation.

To date, users have advised on the trial conception, namely the recruitment strategy, study outcomes, data collection processes, and the study intervention. They have provided feedback on the study protocol before submission to the funding body, and co-written user involvement strategy and lay abstracts. Future involvement during the preparatory phase includes the preparation of study information sheets, training of data assessors and interventionists, and communication recommendations for both the general public and for the recruitment of trial participants. The PFAG will continue to be engaged in the recruitment, data collection (i.e., retention procedures, ongoing reflection on study course), data analysis (i.e., interpretation of results, critical review), and dissemination (i.e., planning, dissemination activities, lay summaries) phases.

Participants and setting

Study setting

The FICUS trial will take place in ICUs in the German-speaking part of Switzerland.

Cluster-level eligibility criteria

Study ICUs need to be able to offer the highest level of patient care and treat patients who are hemodynamically unstable, require ventilation with multiple-organ failure, and need multidisciplinary intervention [47]. They may offer different or combined specialty care, including surgical, trauma, medical, cardiac, or neurological care. ICUs certified by the Swiss Society for Intensive Medicine (SGI) to run at least eight beds are eligible. ICUs with less than 300 admissions per year of patients with an ICU stay of 48 h or longer are excluded, as are ICUs with a protocolized, interprofessional family support program.

Participant-level eligibility criteria

Participants are adult family members (≥ 18 years of age) of critically ill persons who receive treatment in an eligible ICU for at least 48 h. Critically ill persons are defined as those with an expected length of stay in ICU of ≥ 48 h, combined with a life-threatening condition with a high risk of death or long-lasting functional impairment, or a high risk of prolonged mechanical ventilation (> 24 h) as appraised by the intaking clinician. Family members are defined as close others from the patient's perspective, as noted in clinical records or advanced directives, or as legally defined surrogate decision-makers. Legal or blood kinship is not a requirement. They have to be a primary support person of the critically ill person, able to complete the baseline data collection and family-reported questionnaires in German within the required time frame and sign a written informed consent form. Family members of patients with refused general consent will not be eligible to take part. Family members with prior inclusion in the FICUS trial in another ICU, with cognitive inability to understand the study or inability to complete the questionnaire as appraised by clinicians or study recruitment staff, will be excluded. Inability to complete the baseline data collection within the required time frame after admission/study enrollment will lead to exclusion [48].

Informed consent processes

The investigator or his/her delegates will be responsible for obtaining written informed consent. They will explain to each potential family member participant the nature of the study, its purpose, the procedures involved, the expected duration, the potential risks and benefits, and any discomfort it may entail. Each participant will be informed that the participation in the study is voluntary, that s/he may withdraw from the study at any time, and that withdrawal of consent will not affect his/her or their close other's care. Family members will also be asked to state in an additional consent form whether their data obtained in the context of this trial may be used for secondary data analyses.

To extract clinical patient data, consent will be obtained from patients. If the patient has pre-signed a general consent form for the use of routine clinical data for research purposes, no additional consent form is required. If not, and in case the patient lacks cognitive ability to do so upon ICU admission, the participating family member will provide his/her surrogate written informed consent

based on the presumed will of the critically ill patient. As soon as the critically ill patient regains his/her ability of judgment, his/her written informed consent will be obtained.

Study intervention

The family support intervention (FSI), which has been developed and pilot-tested by the coordinating investigator [32, 33], has been slightly adapted in consultation with five users, seven ICU nurses, and three physicians to increase the implementability across the different study ICUs while maintaining its core components [49]. The aims of the FSI are (1) to increase the quality of interaction and communication between families and the ICU team and (2) to improve the ICU team's capacity to provide the necessary care and support to families. The intervention also seeks (1) to strengthen family illness management capacity and well-being and (2) to alleviate the impact of the critical illness and/or loss on family members' mental health.

Explanation of choice of comparator

The FSI will be introduced to the study ICUs of the intervention arm in addition to usual care, which is the control condition.

Usual care

Usual care is defined as a non-protocolized approach to family care and services offered by nurses, physicians, teams, or other health professionals that are an established part of the ICU's routine care delivery before the trial start. These include granting access (visitation), interacting with family members and informing about the patient's condition and treatment (written and oral information), communicating with family members as surrogate decision-makers (communication structure), supporting families (support structures), and making referrals (auxiliary services). ICU staff in control units will be allowed to act upon patient/family needs. However, the introduction of a new protocolized family support intervention, a family nursing or other family support role, or a structured family support pathway is not permitted.

Intervention description

The FSI is grounded in a family-systems nursing approach [50–53]. It proposes that critical illness affects families' affective, cognitive, and behavioral functioning, requiring therapeutic relational nurse-family engagement including relationship-focused and psycho-educational interventions to support family management of illness and to alleviate their suffering. The development was based on evidence around systemic family interventions for chronic illness [54–57], which suggests that the use of a combination of psycho-educational and relationship-focused interventions is most effective in strengthening family and individual health and well-being. The FSI also builds on guideline-based recommendations around ICU care to families that include the use of a specific family consultation role and of structured, interprofessional communication with families to ensure high quality of family care [15] (Fig. 2).

The FSI consists of a new family nursing role that delivers three interacting, closely intertwined intervention components that are mapped to a family care pathway (Fig. 3):

- *Engaging and liaising (family encounters)*: This intervention component involves relationship-building with families, connecting and coordinating family care activities as well as transition and follow-up care for family members and surviving patients.
- *Supporting (therapeutic family conversations)*: This component is based on a relational family systems nursing approach and includes assessing family structure, processes, and resources and supporting families through relationship-focused and psycho-educational interventions at the family systems level.
- *Communicating (interprofessional family meetings)*: This component focuses on structured, interprofessional communication, and shared decision-making with families.

The FSI will be delivered by two to three designated family nurses per ICU in close collaboration with the ICU team. Interventionists will be registered nurses with a certification in ICU nursing or equivalent and training in family systems nursing. They hold a Master of Science in Nursing degree or work under the supervision of an advanced practice nurse. Advanced competencies in family nursing [58] are necessary for the following three reasons: First, families are in a vulnerable situation and/or crisis, which requires specific knowledge and expertise around family processes and illness management. Second, the relational, systemic family interventions require specific knowledge and skills beyond the general competencies of ICU nurses. Third, interventionists will assume a combined role encompassing clinical practice with families, consultation, collaboration, and leadership.

Criteria for discontinuing/modifying interventions

As this is a cluster-randomized trial (RCT), there will be no individual-level assignment that could be discontinued. However, the intervention will be discontinued if a family member withdraws the consent.

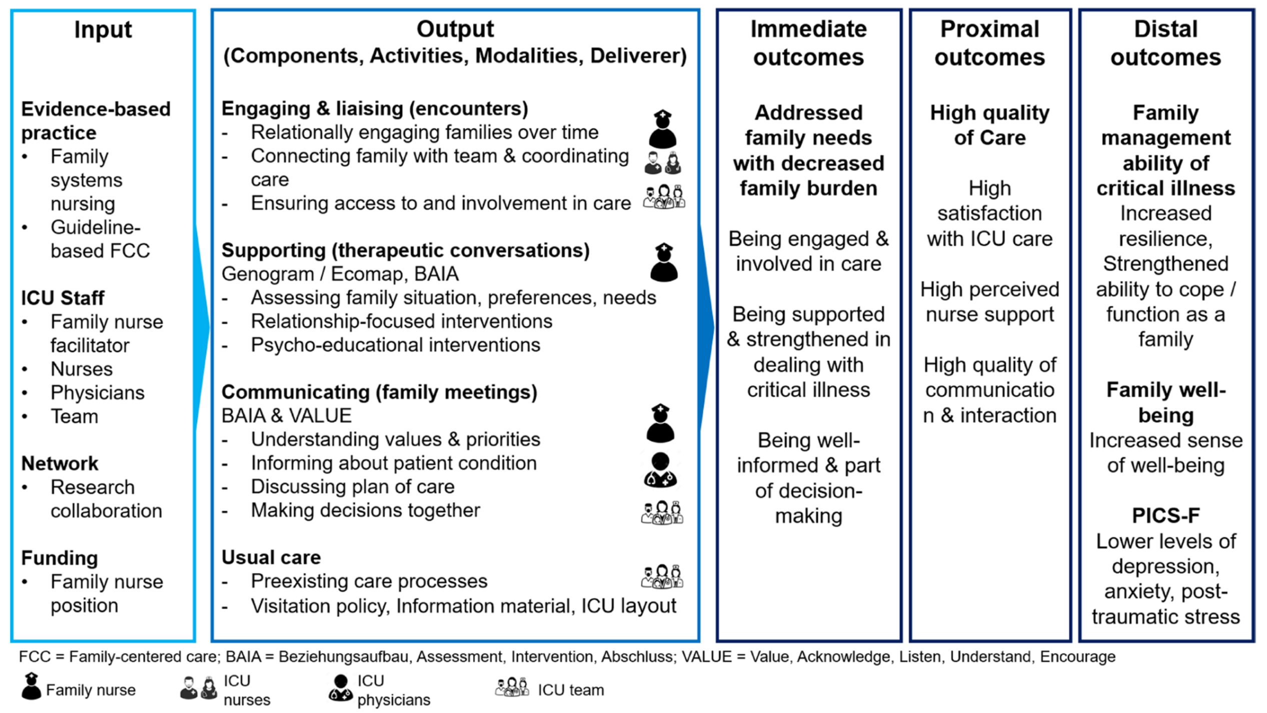

Fig. 2 FSI program logic model

Strategies to improve adherence to interventions

The intervention is standardized in terms of components, intervention content, and a minimum dose along the clinical patient pathway (see Fig. 3) [59]. The frequency of intervention contacts and the dose of each intervention component can be increased and tailored according to the patients' and/or families' preferences and needs. Individual patient or family adherence to a particular health behavior is not required. Fidelity to intervention protocol is ensured at the individual and cluster levels. Adherence will be promoted by a 5-day interventionist training, monthly supervision and case conferences with all study interventionists, and site visits for quality assurance purposes.

Relevant concomitant care permitted/prohibited

The intervention components will be delivered within predefined time windows along the patient pathway, that is, within 96 h after admission, 48 h before or after discharge, and within the first 4 weeks after ICU discharge. A higher intervention dose and frequency of each intervention component are permitted to tailor to patient

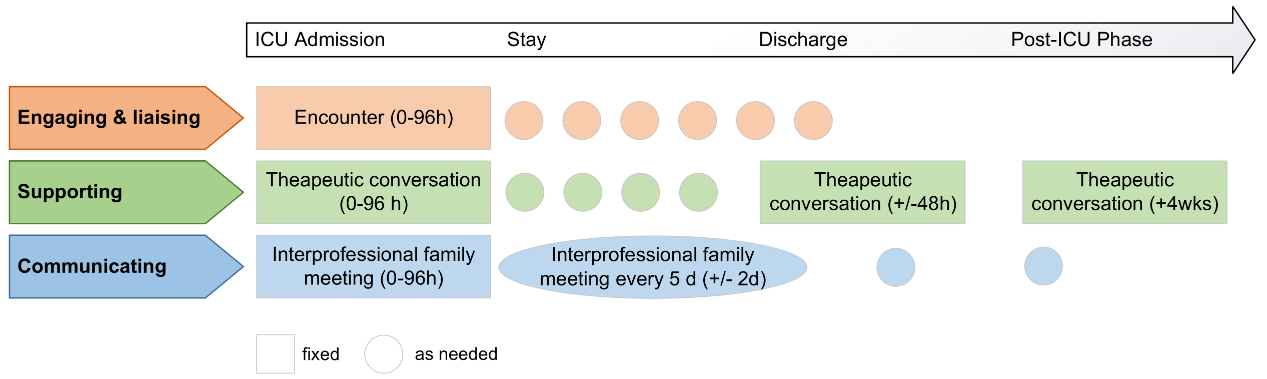

Fig. 3 Family care pathway

illness course, length of ICU stay, and family needs and preferences. Any interaction between ICU shift or primary nurses, ICU, or other treating physicians and other health or social care professionals is explicitly permitted. There are no medical interventions that are prohibited during the study.

Implementation strategy

The FSI will be implemented using a combination of strategies that showed promise in the pilot study [33]. These implementation strategies include leadership endorsement, nurse and physician champions, team education, and external/internal implementation support practitioners [60]. They will be adapted and tailored to the local context to address potential barriers, which will be identified prior to the enrollment start [61, 62].

Outcomes

Primary outcome

The primary outcome is the quality of family care in the ICU, operationalized as family satisfaction with ICU care, which is an established core indicator of the quality of family care [63–65]. It will be assessed at discharge from ICU by the Family Satisfaction in ICU Questionnaire (FS-ICU-24R) [66, 67].

Secondary outcomes

Quality of family care is further operationalized as the quality of communication and nurse support (see Table 1 for measures). As the intervention also targets families' ability to cope with critical illness, family management of critical illness was chosen as a further secondary outcome. Individual family members' mental health will be operationalized to cover the spectrum from well-being to psychological distress. Family management and mental health indicators are more distal than the quality of family care but highly relevant outcomes of interventions that address families' needs for support and promote family capacity and health [68, 69] (Fig. 1). They will be obtained at baseline, discharge, and 3, 6, and 12 months thereafter.

Cluster-level data

Data will also be obtained at the cluster level, that is, from participating ICUs, and include data on ICU characteristics and family care processes and policies.

Process data

Process data will be obtained in the intervention arm, namely information on implementation activities and costs (cluster level) and on intervention delivery (individual level).

Table 1 Individual-level primary and secondary outcomes

Domain/construct	Measure[a]	Range	Cronbach's α[b]	T0	T1	T2	T3	T4
**Quality of family care**								
Satisfaction with care (primary outcome)	Family satisfaction with ICU care (FS-ICU-24R)	0–100	> .85		X			
Quality of communication	Questionnaire on Quality of Physician-Patient Interaction (QQPPI-14)	1–5	.95		X			
Support from nurses	Family Perceived Support Questionnaire (ICE-FPSQ-14)	14–70	> 0.87		X			
**Family management**								
Family functioning	Family Assessment Device - General Functioning Scale (FAD-GF-12)	1–4	.87	X	X	X	X	X
Family resilience	Brief Resilience Scale (BRS-6)[c]	1–5	.85	X	X	X	X	X
**Mental health**								
Subjective well-being	Satisfaction with Life Scale (SWLS-5)	5–35	.89–.92	X	X	X	X	X
	WHO-5 Well-Being Index (WHO-5)	0–100	.92	X	X	X	X	X
	Adapted VAS on Quality of Life (QoL-VAS)	0–100	n/a	X	X	X	X	X
Psychological distress	Distress Thermometer (DT)	0–10	n/a	X	X	X	X	X
	Impact of Events Scale-6 (IES-6)	0–4	.80	X	X	X	X	X
	Hospital Anxiety and Depression Scale (HADS-14)	0–21	> .80	X	X	X	X	X

[a] Use of German versions of measures

[b] References for Cronbach's alpha are listed in the text

[c] Adapted for family

Accompanying studies

To address the second study aim, a three-phased implementation study will be undertaken among the clusters of the intervention arm. First, a context assessment will be conducted to identify barriers to the implementation of FSI in the specific ICU. Next, a tailored implementation plan will be developed, and an adaptive process put in place. Then, the implementation process and outcomes will be evaluated using a mixed-method approach with a case-study format.

An economic evaluation will be undertaken to assess the cost associated with intervention delivery and implementation of the FSI. Working hours per intervention (FSI) will be analyzed in relation to family members' mental health and their absenteeism/presentism at work.

Cluster and participant timelines

Eligible clusters, that is, ICUs that have signed a study agreement, will be enrolled and randomly assigned to the intervention or control arm after cluster-level baseline data collection has been completed. Cluster data will be obtained at baseline, then yearly, and finally after the conclusion of the discharge data collection (first follow-up) of the last participant.

Participant eligibility screening and enrollment will be conducted at the individual level using a consecutive sampling strategy. The participant timeline is displayed in Table 2.

Sample size

Assuming a difference in FS-ICU-24 total score between the intervention and control groups of 5.5 and a within-group standard deviation of the FS-ICU-24 total score of 16.3, as observed in Naef and colleagues [32], we would need a total of 278 subjects in a standard RCT (with $n_{\mathrm{RCT}} = 139$ per arm) with 1:1 randomization to have a power of 0.8 at a significance level of 0.05. However, due to the *design effect* [70], the number of participants required for our cluster-randomized trial is bigger than the sample size of a standard RCT. We assumed an intra-cluster correlation coefficient (ICC) of 0.03 and a coefficient of variation in cluster size of 0.2.

Since the number of clusters is more limiting for this study than cluster size, we used the approach described by Hemming and colleagues [71] to derive the minimum number of clusters required for each condition in our trial. Assuming an unlimited cluster size, the minimum number is simply $n_{\mathrm{RCT}} \times \mathrm{ICC} = 5$ clusters. Increasing the number of clusters to eight (three more than the minimum), the cluster size could be reduced to $\frac{n_{\mathrm{RCT}}}{3}$=47.

We then conducted a series of precise power calculations, using the R packages *clusterPower* [72] and *sse* [73], considering a range of clusters per condition (3,...,20), mean cluster sizes (10–300), and ICCs (0.01–0.1). With an average cluster size of 50 evaluable patients and an ICC of 0.03, 8 clusters need to be randomized to each condition to achieve a power of 0.8. To account for a drop-out rate of 10% of patients within clusters (but no drop-out of whole clusters), an average number of 56 patients should be recruited per cluster.

Recruitment

Eligible ICUs in German-speaking Switzerland were identified based on the list of Swiss Society for Intensive Medicine-certified ICUs. The coordinating investigator contacted eligible ICUs to invite participation and enroll them in the study.

Eligible family members will be recruited within 96 h after patient admission to ICU. Designated ICU nurses or physicians will perform daily eligibility screening of newly admitted patients and potential family participants. They will obtain contact details of at least one family member and provide initial information to family members and hand out the study flyer and information. Study flyers will also be displayed in the waiting area and included in ICU information packages routinely handed to family members. Written study information will also be available on websites.

Next, the clinician or study coordinator will contact eligible family members in person when they visit their critically ill close other or via phone, explain the study, invite participation, and enroll participants. Family members will receive time to decide for or against participation in accordance with ethical guidelines. Participants will not receive payment or any other form of compensation. A thank you letter will be written to them upon study completion.

Assignment of interventions

Clusters will be assigned 1:1 to the intervention and the control arms. To reduce a potential imbalance of study groups at baseline, restricted randomization will be used [74, 75].

Assignment

The assignment of the participating ICUs will be generated after their recruitment and baseline cluster data collection using minimization at a central location. Variables used in the minimization procedure are the degree of specialization (specialized vs. general ICU) and the hospital. The first cluster will be assigned completely at random. To avoid subsequent assignments being deterministic, a random component of 10% will be introduced.

Concealment mechanism

The assignment will be generated by the trial statistician and only be revealed to the investigators after cluster-level baseline data collection has been completed.

Table 2 Participant timeline

Task/study period	Admission to ICU	Screening	Informaiton	ICF	Baseline assessment (T0)	FU 1 to discharge from ICU (T1)	EOT and FU 2–3 months (T2)	FU 3–6 months (T3)	FU 4–12 months (T4) = EOS
Time point	0–24 h	0–48 h	0–72 h	0–96 h	0–96 h	Y (− 24 h/+ 14 days)	Y + 90 days (± 14 days)	Y + 180 days (± 14 days)	Y + 365 days (± 14 days)
**Enrollment**									
Eligibility screen of patient	X								
Identification of eligible family member		X							
Information of family member			X						
Informed consent				X					
**Interventions**									
Intervention arm: family support intervention including intervention log					X	X	X		
Usual care	X	X	X	X	X	X			
**Assessments**									
Patient demographics, health and functional status, care utilization[a]				X	X	X	X	X	X
Family member demographics, self-perceived health, care utilization					X	X	X	X	X
Satisfaction with care (FS-ICU-24R) (primary endpoint)						X			
Quality of communication (QQPPI-14)						X			
Nurse support (ICE-FPSQ-14)						X			
Family functioning (FAD-GF-12)					X	X	X	X	X
Family resilience (BRS-6)					X	X	X	X	X
Distress Thermometer (DT)					X	X	X	X	X
Depression and anxiety (HADS-14)					X	X	X	X	X
Posttraumatic stress (IES-6)					X	X	X	X	X
Life satisfaction (SWLS-5)					X	X	X	X	X
Well-being (WHO-5, QoL-VAS)					X	X	X	X	X

ICF informed consent form, *FU* follow-up, *EOT* end of treatment, *EOS* end of study, *Tx* assessment time point

[a] T0–T1 from clinical record, T2–T4 family member reported

Implementation

Clusters will be identified and enrolled by the coordinating investigator. The cluster assignment will be generated by the trial statistician, using the R package *Minirand* [76]. The coordinating investigator will then communicate the assignment to the local investigators.

Blinding

This is a non-blinded study. Blinding of study participants and clinical and research staff will not be possible due to the nature of the study intervention and cluster design.

Data collection and management

Assessment and collection of cluster-level data

Data at the cluster level will be collected by a paper case report form (pCRF) at baseline before cluster assignment to a study arm, every 12 months, and after the last participant in each cluster has completed the first follow-up (discharge).

ICU characteristics ICU characteristics, such as the number of beds, admissions per year, percentage of high risk and mechanically ventilated patients, length of stay, mortality rate, will be obtained from the most recent Minimal Data Set (MDSi), which is routinely collected using a standardized data capture system provided by the Swiss Society of Intensive Medicine.

Quality of family care To describe usual care processes offered to family members in study ICUs prior to and during the study, a brief form and two self-assessment instruments will be completed by a team of ICU clinicians. These instruments capture usual care processes and family care policies pre-intervention and allow to distinguish between usual care and the FSI post-intervention at the cluster level.

Self-Assessment Tool for Family-Centered Care in ICU [77, 78], which is based on the Society of Critical Care Medicine's guideline for family-centered care [15]. The tool assesses on a 4-point Likert scale the degree to which recommended family engagement practices are currently delivered, such as family presence, support, communication, consultation, and family-friendly layout.

Patient- and Family-Centered Care Organizational Self-Assessment Tool [79] assesses the degree of family engagement at the hospital level, using eleven domains, such as leadership, quality improvement, personnel, mission & visions, and charting and documentation.

Implementation log Implementation strategies used to introduce and maintain the fidelity of the intervention will be recorded by an internal implementation facilitator or local investigator in a structured format. Cost indicators include (but are not necessarily limited to) implementation of structures and processes, acquisition of staff to deliver the intervention, and intervention-specific training.

Assessment and collection of individual-level data

All participants who were assessed for study eligibility, irrespective of whether they were enrolled, considered non-eligible, or were eligible but not enrolled in the study, have to be recorded in a screening log. Investigators will document each study participant in an enrollment log and generate an individual participant schedule. Individual participant-level data will be collected by local study coordinators with an electronic case report form (eCRF), after participant enrollment at admission (baseline), discharge, and 3, 6, and 12 months thereafter via online or paper/pencil survey from family members and via direct data entry from clinical records. To access the online survey, a personalized link will be generated for each participant and assessment time point, which will be sent to participants via email, a text message, or other predetermined means. Participants can opt to answer the survey on a printed paper and pencil version of the eCRF, which will be sent to them with a stamped return envelope. Data collection at ICU admission and discharge will involve either phone or face-to-face interactions to facilitate the timely completion of the survey during this particularly vulnerable phase of a critical illness. During the post-ICU phase, emails or letters will be sent to family members to inform them about the upcoming follow-up data collection. If the survey is not completed within 2 weeks, study coordinators will be followed up by phone every week for 2 weeks. Follow-up calls for data collection reasons will be recorded in the participant list.

Outcome measures obtained via survey The following family-reported outcome measures will be used in their German versions as part of the family member survey (see Table 1):

Family satisfaction in ICU (FS-ICU-24R): The FS-ICU-24-R yields a transformed mean score for overall satisfaction, satisfaction with care (16 items), and satisfaction with involvement in decision-making (ten items). The test-retest reliability of this assessment was high in the original pilot $r = .85$ [65], and the instrument is sensitive to change in several intervention studies. The German version of the FS-ICU-24R has demonstrated no floor or ceiling effects [66, 67].

Questionnaire on the Quality of Physician-Patient Interaction (QQPPI): The QQPPI assesses relationship-building, information exchange, and shared decision-making (14 items) and has been appraised as one of the most psychometrically sound measures of health professional-patient interaction [80] with a test-retest reliability of .59 [81].

Family Perceived Support Questionnaire (ICE-FPSQ): The ICE-FPSQ-14 measures families' perception of support provided by nurses on two subscales: emotional support (nine items) and cognitive support (five items) [82, 83]. It has been translated into German employing a systematic procedure [84] and is currently under psychometric validation by the research group.

Family Assessment Device - General Functioning Scale (FAD-GF): The FAD-GF-12 is used to assess the overall functioning of the family system [85–87] and has demonstrated good test-retest reliability ($r = .60$ after 12 weeks) among patients with PTSD as well as responsiveness in intervention studies [88].

Brief Resilience Scale (BRS): The BRS-6 is conceived to measure the essence of resilience as the ability to bounce back from stress [89], and evidence from intervention studies suggests that the scale is sensitive to change [90, 91]. Its items will be reformulated from "I" to "we" statements to assess family rather than individual ability.

Satisfaction with Life Scale (SWLS-5): Developed to measure the global dimension of subjective well-being [92], the SWLS-5 can detect changes over time as well as responses to treatment [93]. The sum of the scores (range 5–35) can be transformed to an ordinal scale with seven levels ranging from *extremely dissatisfied* to *extremely satisfied* (with 20 indicating neutrality).

WHO-5 Well-being Index (WHO-5): The WHO-5 was developed to measure subjective psychological well-being [94]. It is widely used in European surveys [95, 96] and can detect clinically relevant changes while a score $\leq$ 50 (on a 0–100 scale) is indicative of depression.

Adapted VAS on Quality of Life (QoL-VAS): An adapted version of a visual analog scale (VAS) similar to that used in the EuroQol EQ-5D Questionnaire [97] will measure the self-perceived general quality of life (QoL) rather than health-related QoL.

Distress Thermometer (DT): The Distress Thermometer is originally and still primarily used among cancer patients [98–102], yet it is a generic visual analog scale (VAS) [103]. A common cutoff of 4 indicates potential distress [98, 104], and the thermometer can detect changes over time [105–107].

Impact of Events Scale-6 (IES-6): The IES-R is a widely used instrument to assess psychological distress in ICU family members [3, 108], which measures the presence and severity of symptoms associated with a traumatic event during the past week. The brief version (IES-6) includes two items from each of the three subscales of the IES-R-22 version (intrusion, avoidance, hyperarousal) [109, 110], is highly correlated to the IES-R [111], and detects changes over time [110].

Hospital Anxiety and Depression Scale (HADS): HADS-14 [9, 108, 112] is scored on two subscales (anxiety and depression) and has thresholds for mild/caseness for depression and anxiety, respectively [9, 108, 112]. The HADS has been shown to be an effective measure of psychological distress [113] and can detect changes over time [114, 115] as well as varying degrees of severity [115].

Further family member- and patient-related data At baseline and first follow-up, information on the patients' health condition and ICU treatment will be extracted from the clinical record. After that, family members will be asked to provide proxy information on patients' care utilization and functional status. Family members will complete a demographic form together with information on their work situation, health status, and care utilization as part of the survey at each assessment time point (Table 3).

Intervention log For each intervention contact, the following data will be recorded by interventionists in the eCRF: date, ICU or post-ICU day, duration in minutes, delivery mode (face-to-face, phone, online), number/type of family members and ICU staff present, type of intervention component (encounter, therapeutic conversation, interprofessional family meeting), nurse intervention activity (relational engaging, family assessment, psychoeducational intervention, relationship-focused interventions, liaison and coordination, interprofessional communication, shared decision-making, making referrals), and referrals to auxiliary services. Hence, all concomitant interventions or interventions exceeding usual care have to be recorded. Usual care itself will not be documented.

Retention and complete follow-up

To promote retention at the cluster level, the trial steering committee will hold quarterly study group meetings

Table 3 Patient- and family member-related data

Time point of assessment	Patient					Family member				
	T0	T1	T2	T3	T4	T0	T1	T2	T3	T4
**Demographics**										
Age, gender, civil status (T2–T4 change), Education	X					X		X	X	X
Type of family member (T0), primary carer (yes/no)						X		X	X	X
Co-habiting with patient (yes/no), frequency of contact						X				
Travel time to hospital						X				
**Health status**										
Medical diagnosis (MDSi)	X	X								
Trauma (yes/no); if yes, AIS[a]	X									
Mechanical ventilation and circulary support (T0: yes/no, T1: # of days)	X	X								
SAPS-2[b]	X									
NEMS[c], SOFA[d]	X	X								
Death (yes/no); if yes, date, cause, place[e]	X	X	X	X	X					
Organ donation (yes/no); if yes, organ, surrogate dm)	X									
Functional status (Katz ADL/Lawton IADL)[e]			X	X	X					
Prior/present psychiatric diagnosis (yes/no)						X				
Self-perceived health (VAS)						X	X	X	X	X
**Care utilization**										
Cause of admission	X									
Type of admission (expected, unexpected), admitted from	X									
Surgery (emergency, planned, none)	X									
Previous ICU stays (yes/no)	X									
Prior need for informal/ formal care (yes/no)[f]	X									
Length of ICU stay		X								
Discharge/transfer destination, planned vs. unexpected		X								
Need for informal/formal care (yes / no); if yes, change, hours/frequency, more/less than before ICU treatment)[e]			X	X	X					
Prior/current/new psychological/psychiatric treatment (yes / no)						X	X	X	X	X
Use of prescription drugs						X	X	X	X	X
Current treatment for a chronic condition (yes/no)						X				
Number of family physician visits/hospitalizations (if yes: related to critical illness yes/no/length of stay)							X	X	X	X
Previous ICU experience (as patient, as family of the patient)						X				
Family satisfaction with prior care experience (VAS)						X				
Family satisfaction with care on hospital wards (VAS)								X		

Table 3 (continued)

Time point of assessment	Patient					Family member				
	T0	T1	T2	T3	T4	T0	T1	T2	T3	T4
**Work situation**										
Level of employment, income						X				
Change in level of employment (yes / no); if yes: related to critical illness yes/no), new income							X	X	X	X
Sick leave/absence from work (if yes: # of (half-)days)						X	X	X	X	X
Presenteeism							X	X	X	X

[a] Abbreviated Injury Scale
[b] Sequential Organ Failure Assessment Score
[c] Nine equivalents of nursing manpower use score
[d] Simplified Acute Physiology Score
[e] Patient-related information at T2, T3, T4 obtained from family member
[f] Patient-related information at T0 obtained from family member

to discuss study progress, and provide quarterly communication via newsletters to study sites and staff. The trial manager and further members of the research team will interact regularly with the local study team and site.

To ensure retention of individual study participants, local study teams will make the utmost effort to obtain completed questionnaires at admission, discharge, and at the three post-ICU follow-ups at months 3, 6, and 12 [48]. As the study endpoints rely mainly on the completion of questionnaires by the participants, system-supported automatic notifications on the eCRF completion status will be implemented to ensure data completeness. Study coordinators will check the completeness of the paper-reported outcome measures and follow-up with participants if they identify missing values.

If participants withdraw consent, the reason for withdrawal will be assessed at the time point of withdrawal, and—if the participant agrees—data on the clinical status of the patient will be collected (change in health status/ or death, time point of death). In this case, the intervention and the data collection will be discontinued, and no follow-up is planned. Participants who withdraw or are discontinued before the first follow-up (discharge) will be replaced to achieve the required sample size. The data of the last completed assessment time point will be included in the analysis (as per intention-to-treat analysis).

Data management

For data processing and management, the electronic data capture (EDC) system REDCap [116] will be used. A data monitoring plan specific to the study assessment schedule has been prepared by the coordinating investigator site's clinical trial unit. Data monitoring will be initiated after the inclusion of ten study participants at each study center. Regular monitoring will be undertaken by the trial manager and/or external monitor. Observations and findings will be documented and made available to the coordinating investigator and local study site.

Confidentiality

Role- and user-based access control with personal login regulate permission to access the EDC system, which includes individual user rights for data entry, review, export, and reports. Appropriate coded identification is used to enter participant data into the database; no patient identifying information will be entered into the EDC system.

Data storage

The servers hosting the EDC system and study database are kept in an off-site restricted access locked server room. A copy of the study database will be stored securely by the Clinical Trial Unit at the coordinating investigator's site for at least 10 years. Investigators maintain the essential documents and source data in the Trial Master File and Investigator Site Files and archive interim and final reports in electronic and hard copy format for at least 10 years.

Statistical methods

A more detailed statistical analysis plan will be finalized before database closure. All analyses will be performed in R [117].

Statistical methods for primary and secondary outcomes

The FS-ICU-24-R total score at patient discharge from ICU will be analyzed by a linear mixed-effects model (LMM) with a random intercept per cluster to account for the non-independence of family members from the same cluster. Due to the small number of clusters, the main model will include the treatment (intervention vs. control) as the only explanatory variable in the main analysis, and the Satterthwaite approximation for the denominator degrees of freedom will be used, as recommended by Leyrat and colleagues [118]. The ICC will be estimated from this model based on the residual variance and between-cluster variance.

The following covariate-adjusted sensitivity analyses will be conducted to adjust the treatment effect estimate for potential confounding: At the cluster level, the specialization of the ICU (as used in the cluster randomization), overall ICU staffing, and a quality-of-care indicator will each be added separately to the main model described above. At the individual participant's level, patient age, cause of admission, the SAPS-2 score of the patient (mortality risk), and the family member's previous ICU experience will be added to the main model together (and potentially to the cluster-level adjusted models).

The secondary outcomes regarding the quality of care, which are only measured once at discharge from ICU, will be analyzed with an LMM as described above for the primary outcome. All other secondary outcomes, which are measured at baseline, at discharge from ICU, and at three other follow-up points in time, will be analyzed by an LMM with a random intercept per cluster and a random intercept per family member (nested within the cluster) to additionally account for the non-independence of repeated measurements from the same study participant. The serial autocorrelation of residuals will be modeled using a first-order autoregressive correlation structure. The models will include the treatment (intervention vs. control), the corresponding baseline measurement, the visit, and the visit-treatment interaction.

Methods for additional analyses

Subgroup analyses are planned regarding the primary outcome for the following baseline characteristics: specialization (specialized vs. general ICU), overall ICU staffing, and quality of care indicator (sum score) at the cluster level and patient age, gender, cause of admission (expected vs. unexpected), mortality risk (SPAS-2 score upon admission), type of relationship between patient and family member, family member prior ICU experience, family resilience (BRS-6), family functioning (FAD-FG-12), and anxiety and depression (HADS) at the participant level. A separate LMM will be fitted for each subgroup variable, adding the subgroup variable and the interaction between the subgroup variable and the treatment as explanatory variables to the main model. A significant interaction between a subgroup variable and the treatment would indicate a different treatment effect in the corresponding subgroups (or along a gradient for the continuous subgroup variables). Furthermore, patient survival status and ICU length of stay are two participant-level covariates that are of interest but are measured during or after the intervention and therefore do not qualify as baseline characteristics to adjust for in these subgroup models. However, for exploratory purposes, a model including these two variables together with the treatment and the three two-way interaction terms will be fitted, which will require cautious interpretation. Similarly, a model that includes the intervention dose will be fitted (with a dose of zero for the control group). No interim analyses are planned.

Methods in analysis to handle protocol non-adherence and missing data

The analysis will follow the intention-to-treat principle. Clusters and their participants will be analyzed given the condition to which they were assigned. Should a patient be transferred to another cluster, the family will be analyzed in the original cluster. This will be possible due to individual informed consent. The main analysis will be complete cases, but multilevel multiple imputation of missing data at the participant level [119] will be performed (potentially also to the covariate-adjusted models) to assess the sensitivity of the results with regard to missing outcomes. We do not expect any clusters to be missing as a whole but would exclude them from the analysis if present.

Plans to give access to the full protocol, participant-level data, and statistical code

The full protocol has been made available at ClinicalTrials.gov, NCT05280691. Participant-level data (after anonymization) and statistical code for data analysis will be made available upon reasonable request.

Oversight and monitoring

A trial steering committee; that is, coordinating investigator, co-investigators, trial manager, data manager, statistician, implementation support practitioner, and patient representative, will oversee the trial implementation and conduct at the study sites. Data management and monitoring are provided by the coordinating investigator site's clinical trial unit. The trial will follow national and international standards for good clinical practice and comply with regulatory and ethical requirements. Related or unrelated serious adverse events (SAEs) affecting the participants (family members) are collected and documented in source documents.

Dissemination plans

The scientific output will be published and made available as widely as possible to support knowledge transfer, meta-analyses, and general reproduction efforts. All study centers and study participants will be provided with a lay summary of the study outcome (upon interest), and preliminary findings will be shared through poster presentations, scientific talks, and scientific publications on a national and international level at appropriate platform events typically attended by ICU staff, health care professionals, or the public.

Discussion

The FICUS trial has the dual aim to establish the clinical effectiveness of the FSI, a nurse-delivered, interprofessional multicomponent intervention that is introduced into routine care delivery and to explore its implementation. In line with the MRC framework for developing and evaluating complex interventions [120], the current testing of intervention effectiveness and exploration of implementation builds on previous phases of the FSI development and feasibility-pilot testing [32, 33].

Given the state of research in the field of family intervention in the context of critical illness [22, 24–26, 121, 122], rigorous real-world evidence generated by a randomized controlled design is now necessary. There is a clear need to clarify the clinical effectiveness of specific, yet multicomponent or bundled, nurse-led family interventions [24] that build on guideline-based recommendations for family care in the ICU [15]. Two groups have already tested similar multicomponent interventions combining communication and support with a family navigator role [30, 38], which have shown promising effects on the quality of family care but less clear effects on post-ICU family member health.

The FSI adds, in addition to engagement/liaison and communication, a family systems intervention component. This component, delivered through therapeutic family conversations, is based on a relational family systems nursing approach and includes assessing family

structure, processes, and resources, and supporting families through relationship-focused and psycho-educational interventions at the family systems level [52, 53, 55]. Such a systemic family intervention has been found to be effective in chronic illness [54, 123, 124]. In the context of critical illness, one group has pilot-tested a single component family health conversation intervention, delivered in the early post-ICU phase [125]. They found that families who received the intervention were able to improve their family and social functioning from baseline to month three, and demonstrated better functioning and mental health as well as mental health after twelve months. The FSI, while building on existing knowledge, denotes a novel, feasible and acceptable nurse-led family intervention in ICU, adding a relationship-focused, systemic component to the engagement/liaison and communication/shared-decision-making, spanning from ICU admission into the early post-ICU phase [33].

In addition to the well-established service and clinical outcomes that such family interventions seek to improve, we added family management as a third relevant clinical outcome [125]. We also chose to target a more general critically ill patient group to account for those family members whose close others survive critical illness. Critical illness survivors exhibit high levels of post-ICU physical, cognitive, and mental impairments, requiring considerable and often new-onset family caregiving [8, 68, 126–128]. Given the increased prevalence of critically ill patients due to the COVID-19 pandemic, who have a high prevalence of post-intensive care syndrome [129, 130], more families are affected by the health impact of critical illness. In addition, pandemic-related access restrictions increase family suffering and risk for negative health outcomes [12, 13, 19].

Patient and family member representatives are involved in the trial design and implementation [46, 131]. They actively collaborate with the research team in ensuring that participation in the FICUS trial is meaningful and feasible. In addition, they participate in activities of the FICUS study group and meet regularly to advise the lead researchers on study processes. Patient and public involvement (PPI) has been increasingly called for to ensure the relevance, feasibility, and impact of clinical and critical care research [45, 46, 132]. It has been reported that many challenges often inhibit meaningful engagement and effective collaboration [133, 134]. Hence, the early installment and ongoing partnership with the patient and family representatives, together with the integration of the patient liaison in the research team, build a solid foundation for the implementation of effective PPI in the FICUS trial.

In conclusion, the FICUS trial will evaluate a nurse-led, multicomponent, bundled family support intervention that is embedded in interprofessional care delivery in the ICU and aims to generate high-quality evidence on intervention effectiveness and knowledge of its implementability. Both types of evidence are necessary to determine whether the intervention works as intended in clinical practice and could be scaled up to other ICUs. The study findings will make a significant contribution to the current body of knowledge on effective ICU care that promotes family participation and well-being.

Trial status

At the time of manuscript submission, the FICUS trial has been approved by the responsible Swiss cantonal ethics committees (Nr. 2021-2300). Fifteen of the sixteen required clusters have been recruited. Enrollment of the first participant is expected in spring 2022. Recruitment is expected to be completed by early 2024.

Protocol version: 1.0, 25 October 2021.

Abbreviations

BRS: Brief Resilience Scale; DT: Distress Thermometer; EDC: Electronic Data Capture; FAD-GF: Family Assessment Device - General Functioning Scale; FICUS: Family in Intensive Care UnitS; FSI: Family support intervention; FS-ICU-24R: Family Satisfaction in ICU Questionnaire Revised; eCRF: Electronic case report form; HADS: Hospital Anxiety and Depression Scale; ICC: Intra-cluster correlation coefficient; ICE-FPSQ: Icelandic Family Perceived Support Questionnaire; ICU: Intensive care unit; IES-6: Impact of Events Scale-6; MDSi: Minimal Data Set; MRC: Medical Research Council; QoL-VAS: Adapted VAS on Quality of Life; QQPPI: Questionnaire on the Quality of Physician-Patient Interaction; pCRF: Paper case report form; PFAG: Patient and Family Advisory Group; PICS-F: Post-intensive care syndrome-family; PPI: Patient and Public Involvement; RCT: Randomized controlled trial; SAPS-2: Simplified Acute Physiology Score; SGI: Swiss Society for Intensive Medicine; SWLS-5: Satisfaction with Life Scale; WHO-5: WHO-5 Well-being Index.

Supplementary Information

The online version contains supplementary material available at https://doi.org/10.1186/s13063-022-06454-y.

Additional file 1. SPIRIT Checklist.

Acknowledgements

We are very grateful to the ICU nurse and physician leadership teams who decided to participate in the trial. We would also like to acknowledge the support of our research staff Andrea Thesenvitz, Lotte Verweij, and Saskia Oesch, as well as the four persons acting as patient and family member representatives.

Collaborators: FICUS Study Group

Ursula Betschart, RN, Clinic of Intensive Medicine, Cantonal Hospital St. Gallen

Philipp Buehler, MD, Center of Intensive Care Medicine, Cantonal Hospital Winterthur

Hanna Burkhalter, PhD, RN, Graubünden Cantonal Hospital Chur

Alexander Dullenkopf, MD, Clinic of Anesthesiology and Intensive Care, Cantonal Hospital Frauenfeld, Spital Thurgau AG

Antje Heise, MD, Interdisciplinary Intensive Care Unit, Hospital Thun

Benjamin Hertler, MD, Institute of Intensive Medicine, University Hospital Zurich

Yvonne Liebert, MScN, RN, Department of Nursing, Cantonal Hospital Baden

Fabienne Lussmann, BScN, RN, Center of Intensive Care Medicine, Lucerne Cantonal Hospital

Paola Massarotto, MScN, RN, Institute of Intensive Medicine, University Hospital Zurich

Urs Pietsch, MD, Surgical Intensive Care Unit, Division of Anesthesiology, Intensice Care, Rescue and Pain Medicine, Cantonal Hospital of St. Gallen
Esther Siegrist, MScN, RN, Institute of Intensive Medicine, University Hospital Zurich
Peter Steiger, MD, Institute of Intensive Medicine, University Hospital Zurich
Christoph von Dach, DNP, RN, Cantonal Hospital Olten, Solothurn Hospitals
Monique Wenzler, RN, Institute for Anesthesiology and Intensive Medicine, Hirslanden Clinic Zurich, Hirslanden AG
Jan Wiegand, MD, Interdisciplinary Intensive Care, Lindenhof Hospital Bern
Björn Zante, MD, University Department of Intensive Care Medicine, Inselspital, University Hospital Bern

Authors' contributions
RN initiated the study conception and design of the FICUS trial, was responsible for the funding application, and acts as the sponsor and coordinating investigator. MF, MMJ, and MRu made a substantial contribution to the conception and trial design and co-wrote the funding application. MF and MMJ will be responsible for the implementation of the trial. SvF designed the statistical analysis plan. MRi and RN co-wrote the manuscript. MF, MMJ, SvF, JS, and MRu critically reviewed the manuscript for important intellectual content. All authors have reviewed the drafts and approved the final version.

Funding
The study is funded by the Swiss National Science Fund (SNSF, No. 33IC30_198778/1). The funder has no role in the development of the study design; collection, analysis, or interpretation of the data; writing of the manuscript; or decision to submit the manuscript for publication.

Availability of data and materials
Data sharing is not applicable to this article as no datasets were generated or analyzed during the current study (study protocol).

Declarations

Ethics approval and consent to participate
Cantonal Ethics Committee of Zurich BASEC ID 2021-02300 approval on 28 January 2022. Written, informed consent will be obtained from all participants.

Consent for publication
Not applicable.

Competing interests
The authors declare that they have no competing interests.

Author details
[1]Institute for Implementation Science in Health Care, Faculty of Medicine, University of Zurich, Universitätsstrasse 84, 8006 Zurich, Switzerland. [2]Centre of Clinical Nursing Science, University Hospital Zurich, Rämistrasse 100, 8091 Zurich, Switzerland. [3]Surgical Intensive Care Unit, Division of Anesthesiology, Intensive Care, Rescue and Pain Medicine, Cantonal Hospital of St. Gallen, Rorschacher Strasse 95, 9007 St. Gallen, Switzerland. [4]Department of Intensive Care Medicine, University Hospital Bern, Inselspital, University of Bern, Freiburgstrasse 16, CH10, Bern, Switzerland. [5]Department of Biostatistics, Epidemiology, Biostatistics, and Prevention Institute, Faculty of Medicine, University of Zurich, Hirschengraben 84, 8001 Zurich, Switzerland. [6]Bern, Switzerland. [7]Department of Psychiatry, Psychotherapy, and Psychosomatics, Psychiatric University Hospital Zurich, University of Zurich, Zurich, Switzerland. [8]Center for Psychiatry and Psychotherapy, Clinic Zugersee, Triaplus AG, Widenstrasse 55, 6317 Oberwil-Zug, Switzerland.

Received: 15 March 2022 Accepted: 8 June 2022
Published online: 27 June 2022

References
1. Eggenberger SK, Nelms TP. Being family: the family experience when an adult member is hospitalized with a critical illness. J Clin Nurs. 2007;16(9):1618–28.
2. Hopkins RO. Emotional processing/psychological morbidity in the ICU. In: Netzer G, editor. Families in the Intensive care unit: a guide to understanding, engaging, and supporting at the bedside. Cham: Springer Nature; 2018. p. 31–48.
3. Alfheim HB, Hofso K, Smastuen MC, Toien K, Rosseland LA, Rustoen T. Post-traumatic stress symptoms in family caregivers of intensive care unit patients: a longitudinal study. Intensive Crit Care Nurs. 2019;50:5–10.
4. Minton C, Batten L, Huntington A. A multicase study of prolonged critical illness in the intensive care unit: families' experiences. Intensive Crit Care Nurs. 2019;50:21–7.
5. Turner-Cobb JM, Smith PC, Ramchandani P, Begen FM, Padkin A. The acute psychobiological impact of the intensive care experience on relatives. Psychol Health Med. 2016;21(1):20–6.
6. Alfheim HB, Rosseland LA, Hofsø K, Småstuen MC, Rustøen T. Multiple symptoms in family caregivers of intensive care unit patients. J Pain Symptom Manage. 2018;55(2):387–94.
7. Kentish-Barnes N, Chaize M, Seegers V, Legriel S, Cariou A, Jaber S, et al. Complicated grief after death of a relative in the intensive care unit. Eur Respir J. 2015;45(5):1341–52.
8. Inoue S, Hatakeyama J, Kondo Y, Hifumi T, Sakuramoto H, Kawasaki T, et al. Post-intensive care syndrome: its pathophysiology, prevention, and future directions. Acute Med Surg. 2019;6(3):233–46.
9. Davidson JE, Jones C, Bienvenu OJ. Family responses to critical illness: postintensive care syndrome - family. Crit Care Med. 2012;40(2):618–24.
10. Harvey MA, Davidson J. Long-term consequences of critical illness: a new opportunity for high-impact critical care nurses. Crit Care Nurse. 2011;31(5):12–5.
11. van Beusekom I, Bakhshi-Raiez F, de Keizer NF, Dongelmans DA, van der Schaaf M. Reported burden on informal caregivers of ICU survivors: a literature review. Crit Care. 2016;20:16.
12. Cattelan J, Castellano S, Merdji H, Audusseau J, Claude B, Feuillassier L, et al. Psychological effects of remote-only communication among reference persons of ICU patients during COVID-19 pandemic. J Intensive Care. 2021;9(1):5.
13. Kentish-Barnes N, Cohen-Solal Z, Morin L, Souppart V, Pochard F, Azoulay E. Lived experiences of family members of patients with severe COVID-19 who died in intensive care units in France. JAMA Netw Open. 2021;4(6):e2113355.
14. Netzer G, editor. Families in the intensive care unit: a guide to understanding, engaging, and supporting at the bedside. Springer Nature: Cham; 2018.
15. Davidson JE, Aslakson RA, Long AC, Puntillo KA, Kross EK, Hart J, et al. Guidelines for family-centered care in the neonatal, pediatric, and adult ICU. Crit Care Med. 2017;45(1):103–28.
16. Rawal G, Yadav S, Kumar R. Post-intensive care syndrome: an overview. J Translat Internal Med. 2017;5(2):90–2.
17. Azoulay É, Curtis JR, Kentish-Barnes N. Ten reasons for focusing on the care we provide for family members of critically ill patients with COVID-19. Intensive Care Med. 2021;47(2):230–3.
18. Montauk TR, Kuhl EA. COVID-related family separation and trauma in the intensive care unit. Psychol Trauma. 2020;12(S1):S96–S7.
19. Zante B, Erne K, Grossenbacher J, Camenisch SA, Schefold JC, Jeitziner MM. Symptoms of post-traumatic stress disorder (PTSD) in next of kin during suspension of ICU visits during the COVID-19 pandemic: a prospective observational study. BMC Psychiatry. 2021;21(1):477.
20. Gerritsen RT, Hartog CS, Curtis JR. New developments in the provision of family-centered care in the intensive care unit. Intensive Care Med. 2017;43(4):550–3.
21. Lee HW, Park Y, Jang EJ, Lee YJ. Intensive care unit length of stay is reduced by protocolized family support intervention: a systematic review and meta-analysis. Intensive Care Med. 2019;45(8):1072–81.
22. Goldfarb MJ, Bibas L, Bartlett V, Jones H, Khan N. Outcomes of patient- and family-centered care interventions in the ICU: a systematic review and meta-analysis. Crit Care Med. 2017;45(10):1751–61.
23. Scheunemann LP, McDevitt M, Carson SS, Hanson LC. Randomized, controlled trials of interventions to improve communication in intensive care: a systematic review. Chest. 2011;139(3):543–54.

24. Kiwanuka F, Sak-Dankosky N, Alemayehu YH, Nanyonga RC, Kvist T. The evidence base of nurse-led family interventions for improving family outcomes in adult critical care settings: a mixed method systematic review. Int J Nurs Stud. 2022;125:104100.
25. Xyrichis A, Fletcher S, Philippou J, Brearley S, Terblanche M, Rafferty AM. Interventions to promote family member involvement in adult critical care settings: a systematic review. BMJ Open. 2021;11(4):e042556.
26. Zante B, Camenisch SA, Schefold JC. Interventions in post-intensive care syndrome-family: a systematic literature review. Crit Care Med. 2020;48(9):e835–e40.
27. Iftikhar IH, Goldfarb M. Effect of patient- and family-centered care interventions on ICU length of stay. Crit Care Med. 2018;46:e186–7.
28. Shelton W, Moore CD, Socaris S, Gao J, Dowling J. The effect of a family support intervention on family satisfaction, length-of-stay, and cost of care in the intensive care unit. Crit Care Med. 2010;38(5):1315–20.
29. White DB, Cua SM, Walk R, Pollice L, Weissfeld L, Hong S, et al. Nurse-led intervention to improve surrogate decision making for patients with advanced critical illness. Am J Crit Care. 2012;21(6):396–409.
30. White DB, Angus DC, Shields AM, Buddadhumaruk P, Pidro C, Paner C, et al. A randomized trial of a family-support intervention in intensive care units. N Engl J Med. 2018;378(25):2365–75.
31. Moore CD, Bernardini GL, Hinerman R, Sigond K, Dowling J, Wang DB, et al. The effect of a family support intervention on physician, nurse, and family perceptions of care in the surgical, neurological, and medical intensive care units. Crit Care Nurs Q. 2012;35(4):378–87.
32. Naef R, von Felten S, Petry H, Ernst J, Massarotto P. Impact of a nurse-led family support intervention on family members' satisfaction with intensive care and psychological wellbeing: a mixed-methods evaluation. Aust Crit Care. 2021;34(6):594–603.
33. Naef R, Massarotto P, Petry H. Families' and health professionals' experience with a nurse-led family support intervention in ICU: a qualitative evaluation study. Intensive Crit Care Nurs. 2020;61:102916.
34. Torke AM, Wocial LD, Johns SA, Sachs GA, Callahan CM, Bosslet GT, et al. The family navigator: a pilot intervention to support intensive care unit family surrogates. Am J Crit Care. 2016;25(6):498–507.
35. Taylor SP, Short RT, Asher AM, Muthukkumar R, Sanka P. Family engagement navigators: a novel program to facilitate family-centered care in the intensive care unit during COVID-19. NEJM Catalyst. 2020. https://doi.org/10.1056/CAT.20.0396.
36. Lopez-Soto C, Bates E, Anderson C, Saha S, Adams L, Aulakh A, et al. The role of a liaison team in ICU family communication during the COVID 19 pandemic. J Pain Symptom Manage. 2021;62(3):e112–9.
37. Keen A, George A, Stuck BT, Snyder C, Fleck K, Azar J, et al. Nurse perceptions of a nurse family liaison implemented during the COVID-19 pandemic: a qualitative thematic analysis. Intensive Crit Care Nurs. 2021;70:103185.
38. Curtis JR, Treece PD, Nielsen EL, Gold J, Ciechanowski PS, Shannon SE, et al. Randomized trial of communication facilitators to reduce family distress and intensity of end-of-life care. Am J Respir Crit Care Med. 2016;193(2):154–62.
39. Kentish-Barnes N, Chevret S, Valade S, Jaber S, Kerhuel L, Guisset O, et al. A three-step support strategy for relatives of patients dying in the intensive care unit: a cluster randomised trial. Lancet. 2022;399:656–64.
40. Liu K, Nakamura K, Katsukawa H, Nydahl P, Ely EW, Kudchadkar SR, et al. Implementation of the ABCDEF bundle for critically Ill ICU patients during the COVID-19 pandemic: a multi-national 1-day point prevalence study. Front Med. 2021;8:735860.
41. Kleinpell R, Heyland DK, Lipman J, Sprung CL, Levy M, Mer M, et al. Patient and family engagement in the ICU: report from the task force of the World Federation of Societies of Intensive and Critical Care Medicine. J Crit Care. 2018;48:251–6.
42. Landes SJ, McBain SA, Curran GM. An introduction to effectiveness-implementation hybrid designs. Psychiatry Res. 2019;280:112513.
43. Chan A-W, Tetzlaff JM, Gøtzsche PC, Altman DG, Mann H, Berlin JA, et al. SPIRIT 2013 explanation and elaboration: guidance for protocols of clinical trials. BMJ. 2013;346:e7586.
44. Pandya-Wood R, Barron DS, Elliott J. A framework for public involvement at the design stage of NHS health and social care research: time to develop ethically conscious standards. Res Involve Engage. 2017;3(1):6.
45. Burns KEA, Devlin JW, Hill NS. Patient and family engagement in designing and implementing a weaning trial: a novel research paradigm in critical care. Chest. 2017;152(4):707–11.
46. Greenhalgh T, Hinton L, Finlay T, Macfarlane A, Fahy N, Clyde B, et al. Frameworks for supporting patient and public involvement in research: systematic review and co-design pilot. Health Expect. 2019;22(4):785–801.
47. Marshall JC, Bosco L, Adhikari NK, Connolly B, Diaz JV, Dorman T, et al. What is an intensive care unit? A report of the task force of the World Federation of Societies of Intensive and Critical Care Medicine. J Crit Care. 2017;37:270–6.
48. Calvert M, Kyte D, Mercieca-Bebber R, Slade A, Chan AW, King MT, et al. Guidelines for inclusion of patient-reported outcomes in clinical trial protocols: the SPIRIT-PRO Extension. Jama. 2018;319(5):483–94.
49. Movsisyan A, Arnold L, Evans R, Hallingberg B, Moore G, O'Cathain A, et al. Adapting evidence-informed complex population health interventions for new contexts: a systematic review of guidance. Implement Sci. 2019;14(1):105.
50. Doane GH, Varcoe C. Family nursing as relational inquiry: developing health-promoting practice. Philadelphia: Lippincott Williams & Wilkins; 2005.
51. Gottlieb LN. Strengths-based nursing care: health and healing for person and family: Springer Publishing Company; 2012.
52. Rolland JS, Emanuel LL, Torke AM. Applying a family systems lens to proxy decision making in clinical practice and research. Fam Syst Health. 2017;35(1):7–17.
53. Shajani Z, Snell D. Wright & Leahey's nurses and families: a guide to family assessment and intervention. 7th ed. Davis: F. A; 2019.
54. Hartmann M, Bazner E, Wild B, Eisler I, Herzog W. Effects of interventions involving the family in treatment of adult patients with chronic diseases: a meta-analysis. Psychother Psychosom. 2010;79(3):136–48.
55. Östlund U, Persson C. Examining family responses to family systems nursing interventions: an integrative review. J Fam Nurs. 2014;20(3):259–86.
56. Mahrer-Imhof R, Bruylands M. Is it beneficial to involve family members? A literature review to psychosocial interventions in family-centred nursing [German]. Pflege. 2014;27(5):285–96.
57. Chesla CA. Do family interventions improve health? J Fam Nurs. 2010;16(4):355–77.
58. International Family Nursing Association. Position statement on advanced competencies for family nursing. 2017. https://internationalfamilynursing.org/2017/05/19/advanced-practice-competencies/. Accessed 22 Feb 2022.
59. Sidani S, Braden CJ. Design, evaluation, and translation of nursing interventions. Chichester: Wiley-Blackwell; 2011.
60. Albers B, Metz A, Burke K. Implementation support practitioners – a proposal for consolidating a diverse evidence base. BMC Health Serv Res. 2020;20(1):368.
61. Baker R, Camosso-Stefinovic J, Gillies C, Shaw EJ, Cheater F, Flottorp SA, et al. Tailored interventions to address determinants of practice. Cochrane Database Syst Rev. 2015;4:CD005470.
62. Powell BJ, Beidas RS, Lewis CC, Aarons GA, McMillen JC, Proctor EK, et al. Methods to improve the selection and tailoring of implementation strategies. J Behav Health Serv Res. 2017;44(2):177–94.
63. Padilla Fortunatti C, Munro CL. Factors associated with family satisfaction in the adult intensive care unit: a literature review. Aust Crit Care. 2021;31(5):318–24.
64. Scott P, Thomson P, Shepherd A. Families of patients in ICU: a scoping review of their needs and satisfaction with care. Nursing Open. 2019;6(3):698–712.
65. van den Broek JM, Brunsveld-Reinders AH, Zedlitz AM, Girbes AR, de Jonge E, Arbous MS. Questionnaires on family satisfaction in the adult ICU: a systematic review including psychometric properties. Crit Care Med. 2015;43(8):1731–44.
66. Harrison DA, Ferrando-Vivas P, Wright SE, McColl E, Rowan KM, Investigators FS. Psychometric assessment of the family satisfaction in the intensive care unit (FS-ICU-24) questionnaire among family members of patients admitted to adult general ICUs in the United Kingdom. Intensive Care Med Exp. 2015;3(Suppl 1):A152.
67. Stricker KH, Niemann S, Bugnon S, Wurz J, Rohrer O, Rothen HU. Family satisfaction in the intensive care unit: cross-cultural adaptation of a questionnaire. J Crit Care. 2007;22(3):204–11.

68. Cameron JI, Chu LM, Matte A, Tomlinson G, Chan L, Thomas C, et al. One-year outcomes in caregivers of critically ill patients. N Engl J Med. 2016;374(19):1831–41.
69. Stepanovic K, Van J, Jackson JJ. Family psychological morbidity after the intensive care unit. In: Netzer G, editor. Families in the intensive care unit: a guide to understanding, engaging, and supporting at the bedside. Cham: Springer Nature; 2018. p. 49–60.
70. Kerry SM, Bland JM. Sample size in cluster randomisation. Bmj. 1998;316(7130):549.
71. Hemming K, Eldridge S, Forbes G, Weijer C, Taljaard M. How to design efficient cluster randomised trials. BMJ. 2017;358:j3064.
72. Kleinman K, Moyer J, Reich N, Obeng D. clusterPower: power calculations for cluster-randomized and cluster-randomized crossover trials. R package version 0.6.111; 2017.
73. Fabbro T. sse: sample size estimation. R package version 0.7-13; 2019.
74. Esserman D, Allore HG, Travison TG. The method of randomization for cluster-randomized trials: challenges of including patients with multiple chronic conditions. Int J Stat Med Res. 2016;5(1):2–7.
75. Ivers NM, Halperin IJ, Barnsley J, Grimshaw JM, Shah BR, Tu K, et al. Allocation techniques for balance at baseline in cluster randomized trials: a methodological review. Trials. 2012;13:120.
76. Jin M, Polis A, Hartzel J. R package 'Minirand'. 0.1.3 ed: CRAN; 2020. https://cran.r-project.org/web/packages/Minirand/Minirand.pdf
77. Hwang DY, El-Kareh R, Davidson JE. Implementing intensive care unit family-centered care: resources to identify and address gaps. AACN Adv Crit Care. 2017;28(2):148–54.
78. Hwang DY, Yagoda D, Perrey HM, Tehan TM, Guanci M, Ananian L, et al. Assessment of satisfaction with care among family members of survivors in a neuroscience intensive care unit. J Neurosci Nurs. 2014;46(2):106–16.
79. Institute for Healthcare Improvement. Patient- and family-centered care organizational self-assessment tool. 2013. https://www.nichq.org/resource/family-engagement-guide-role-family-health-partners-quality-improvement-within-pediatric. Accessed 22 Feb 2022.
80. Zill J, Christalle E, Müller E, Härter M, Dirmaier J, Scholl I. Measurement of physician-patient communication—a systematic review. PLoS One. 2014;9(12):e112637.
81. Bieber C, Müller KG, Nicolai J, Hartmann M, Eich W. How does your doctor talk with you? Preliminary validation of a brief patient self-report questionnaire on the quality of physician-patient interaction. J Clin Psychol Med Settings. 2010;17(2):125–36.
82. Bruce E, Dorell A, Lindh V, Erlingsson C, Lindkvist M, Sundin K. Translation and testing of the Swedish version of Iceland-Family Perceived Support Questionnaire with parents of children with congenital heart defects. J Fam Nurs. 2016;22(3):298–320.
83. Sveinbjarnardottir EK, Svavarsdottir EK, Hrafnkelsson B. Psychometric development of the Iceland-Family Perceived Support Questionnaire (ICE-FPSQ). J Fam Nurs. 2012;18(3):328–52.
84. Sousa VD, Rojjanasrirat W. Translation, adaptation and validation of instruments or scales for use in cross-cultural health care research: a clear and user-friendly guideline. J Eval Clin Pract. 2011;17(2):268–74.
85. Mansfield AK, Keitner GI, Dealy J. The family assessment device: an update. Fam Process. 2015;54(1):82–93.
86. Epstein N, Baldwin L, Bishop D. The McMaster Family Assessment Device. J Marital Fam Ther. 1983;9(2):171–80.
87. Beierlein V, Bultmann JC, Möller B, von Klitzing K, Flechtner H-H, Resch F, et al. Measuring family functioning in families with parental cancer: reliability and validity of the German adaptation of the Family Assessment Device (FAD). J Psychosom Res. 2016;93:110–7
88. Staccini L, Tomba E, Grandi S, Keitner GI. The evaluation of family functioning by the family assessment device: a systematic review of studies in adult clinical populations. Fam Process. 2015;54(1):94–115.
89. Smith BW, Dalen J, Wiggins K, Tooley E, Christopher P, Bernard J. The brief resilience scale: assessing the ability to bounce back. Int J Behav Med. 2008;15(3):194–200.
90. Bluth K, Eisenlohr-Moul TA. Response to a mindful self-compassion intervention in teens: a within-person association of mindfulness, self-compassion, and emotional well-being outcomes. J Adolesc. 2017;57:108–18.
91. Romcevich LE, Reed S, Flowers SR, Kemper KJ, Mahan JD. Mind-body skills training for resident wellness: a pilot study of a brief mindfulness intervention. J Med Educ Curric Dev. 2018;5:1–10.
92. Diener E, Emmons RA, Larsen RJ, Griffin S. The Satisfaction With Life Scale. J Pers Assess. 1985;49(1):71–5.
93. Pavot W, Diener E. The Satisfaction With Life Scale and the emerging construct of life satisfaction. J Positive Psychol. 2008;3(2):137–52.
94. Bech P, Olsen LR, Kjoller M, Rasmussen NK. Measuring well-being rather than the absence of distress symptoms: a comparison of the SF-36 Mental Health subscale and the WHO-Five Well-Being Scale. Int J Methods Psychiatr Res. 2003;12(2):85–91.
95. Eurofund. European Quality of Life Survey 2016. Luxembourg: Publications Office of the European Union; 2017. [1-114]. https://www.eurofound.europa.eu/surveys/european-quality-of-life-surveys/european-quality-of-life-survey-2016. Accessed 22 Feb 2022
96. Topp CW, Østergaard SD, Søndergaard S, Bech P. The WHO-5 Well-Being Index: a systematic review of the literature. Psychother Psychosom. 2015;84(3):167–76.
97. Rabin R, de Charro F. EQ-5D: a measure of health status from the EuroQol Group. Ann Med. 2001;33(5):337–43.
98. Donovan KA, Grassi L, McGinty HL, Jacobsen PB. Validation of the Distress Thermometer worldwide: state of the science. Psychooncology. 2014;23(3):241–50.
99. Garrubba M. Use of Distress Thermometers in settings outside of oncology Centre for Clinical Effectiveness: Monash Health; 2019. https://monashhealth.org/health-professionals/cce/cce-publications/. Accessed 22 Feb 2022.
100. Mehnert A, Müller D, Lehmann C, Koch U. Die deutsche Version des NCCN Distress-Thermometers. Zeitschrift für Psychiatrie, Psychol und Psychother. 2006;54(3):213–23.
101. Zwahlen D, Hagenbuch N, Carley MI, Recklitis CJ, Buchi S. Screening cancer patients' families with the Distress Thermometer (DT): a validation study. Psychooncology. 2008;17:959–66.
102. Zwahlen D, Hagenbuch N, Jenewein J, Carley MI, Buchi S. Adopting a family approach to theory and practice: measuring distress in cancer patient-partner dyads with the Distress Thermometer. Psychooncology. 2011;20(4):394–403.
103. McCormack HM, Horne DJ, Sheather S. Clinical applications of visual analogue scales: a critical review. Psychol Med. 1988;18(4):1007–19.
104. Ma X, Zhang J, Zhong W, Shu C, Wang F, Wen J, et al. The diagnostic role of a short screening tool--the Distress Thermometer: a meta-analysis. Support Care Cancer. 2014;22(7):1741–55.
105. Gessler S, Low J, Daniells E, Williams R, Brough V, Tookman A, et al. Screening for distress in cancer patients: is the Distress Thermometer a valid measure in the UK and does it measure change over time? A prospective validation study. Psychooncology. 2008;17(6):538–47.
106. Ohnhäuser S, Wüller J, Foldenauer AC, Pastrana T. Changes in distress measured by the Distress Thermometer as reported by patients in home palliative care in Germany. J Palliat Care. 2018;33(1):39–46.
107. Thalen-Lindstrom A, Larsson G, Hellbom M, Glimelius B, Johansson B. Validation of the Distress Thermometer in a Swedish population of oncology patients; accuracy of changes during six months. Eur J Oncol Nurs. 2013;17(5):625–63.
108. Kentish-Barnes N, Lemiale V, Chaize M, Pochard F, Azoulay E. Assessing burden in families of critical care patients. Crit Care Med. 2009;37(10 Suppl):S448–56.
109. Maercker A, Schützwohl M. Erfassung von psychischen Belastungssfolgen: Die Impact of Event Skala-revidierte Version. Diagnostica. 1998;44:130–41.
110. Weiss DS, Marmar CR. Assessing psychological trauma and PTSD. New York: Guilford; 1996.
111. Thoresen S, Tambs K, Hussain A, Heir T, Johansen VA, Bisson JI. Brief measure of posttraumatic stress reactions: impact of Event Scale-6. Soc Psychiatry Psychiatr Epidemiol. 2010;45(3):405–12.
112. McAdam JL, Puntillo K. Symptoms experienced by family members of patients in intensive care units. Am J Crit Care. 2009;18(3):200–9 quiz 10.
113. Norton S, Cosco T, Doyle F, Done J, Sacker A. The Hospital Anxiety and Depression Scale: a meta confirmatory factor analysis. J Psychosom Res. 2013;74(1):74–81.

114. Angst F, Verra ML, Lehmann S, Aeschlimann A. Responsiveness of five condition-specific and generic outcome assessment instruments for chronic pain. BMC Med Res Methodol. 2008;8:26.
115. Herrmann C. International experiences with the Hospital Anxiety and Depression Scale--a review of validation data and clinical results. J Psychosom Res. 1997;42(1):17–41.
116. Research Electronic Data Capture software: REDCap Consortium; 2022. https://www.project-redcap.org. Accessed 22 Feb 2022.
117. R Core Team. R: a language and environment for statistical computing Vienna, Austria: R Foundation for Statistical Computing; 2021. http://www.R-project.org. Accessed 22 Feb 2022.
118. Leyrat C, Morgan KE, Leurent B, Kahan BC. Cluster randomized trials with a small number of clusters: which analyses should be used? Int J Epidemiol. 2018;47(3):1012.
119. van Buuren S. Flexible imputation of missing data. 2nd ed: Chapman and Hall/CRC; 2018.
120. Skivington K, Matthews L, Simpson SA, Craig P, Baird J, Blazeby JM, et al. A new framework for developing and evaluating complex interventions: update of Medical Research Council guidance. BMJ. 2021;374:n2061.
121. Kynoch K, Chang A, Coyer F, McArdle A. The effectiveness of interventions to meet family needs of critically ill patients in an adult intensive care unit: a systematic review update. JBI Database System Rev Implement Rep. 2016;14(3):181–234.
122. Mackie BR, Mitchell M, Marshall PA. The impact of interventions that promote family involvement in care on adult acute-care wards: an integrative review. Collegian. 2018;25(1):131–40.
123. Deek H, Hamilton S, Brown N, Inglis SC, Digiacomo M, Newton PJ, et al. Family-centred approaches to healthcare interventions in chronic diseases in adults: a quantitative systematic review. J Adv Nurs. 2016;72(5):968–79.
124. Hopkinson JB, Brown JC, Okamoto I, Addington-Hall JM. The effectiveness of patient-family carer (couple) intervention for the management of symptoms and other health-related problems in people affected by cancer: a systematic literature search and narrative review. J Pain Symptom Manage. 2012;43(1):111–42.
125. Ågren S, Eriksson A, Fredrikson M, Hollman-Frisman G, Orwelius L. The health promoting conversations intervention for families with a critically ill relative: a pilot study. Intensive Crit Care Nurs. 2019;50:103–10.
126. Steenbergen S, Rijkenberg S, Adonis T, Kroeze G, van Stijn I, Endeman H. Long-term treated intensive care patients outcomes: the one-year mortality rate, quality of life, health care use and long-term complications as reported by general practitioners. BMC Anesthesiol. 2015;15:142.
127. Wintermann GB, Petrowski K, Weidner K, Strauß B, Rosendahl J. Impact of post-traumatic stress symptoms on the health-related quality of life in a cohort study with chronically critically ill patients and their partners: age matters. Crit Care. 2019;23(1):39.
128. Torres J, Veiga C, Pinto F, Ferreira A, Sousa F, Jacinto R, et al. Caregiving burden: the impact of post intensive care syndrome. Intensive Care Med Exp. 2015;3(1):A967.
129. Banno A, Hifumi T, Takahashi Y, Soh M, Sakaguchi A, Shimano S, et al. One-year outcomes of postintensive care syndrome in critically ill coronavirus disease 2019 patients: a single institutional study. Crit Care Explor. 2021;3(12):e0595.
130. Weidman K, LaFond E, Hoffman KL, Goyal P, Parkhurst CN, Derry-Vick H, et al. Post-ICU syndrome in a cohort of COVID-19 survivors in New York City. Ann Am Thorac Soc. 2021. https://doi.org/10.1513/AnnalsATS.202104-520OC.
131. Concannon TW, Grant S, Welch V, Petkovic J, Selby J, Crowe S, et al. Practical guidance for involving stakeholders in health research. J Gen Intern Med. 2019;34(3):458–63.
132. Swiss Clinical Trial Organisation. Guide for researchers to address patient and public involvement (PPI) in clinical trials Bern: Swiss Clinical Trial Organisation & Swiss National Science Fund; 2021. https://www.scto.ch/de/publications/fact-sheets.html. Accessed 22 Feb 2022.
133. Mathie E, Smeeton N, Munday D, Rhodes G, Wythe H, Jones J. The role of patient and public involvement leads in facilitating feedback: "invisible work". Res Involve Engage. 2020;6(1):40.
134. Selman LE, Clement C, Douglas M, Douglas K, Taylor J, Metcalfe C, et al. Patient and public involvement in randomised clinical trials: a mixed-methods study of a clinical trials unit to identify good practice, barriers and facilitators. Trials. 2021;22(1):735.

Publisher's Note

Springer Nature remains neutral with regard to jurisdictional claims in published maps and institutional affiliations.

Ready to submit your research? Choose BMC and benefit from:

- fast, convenient online submission
- thorough peer review by experienced researchers in your field
- rapid publication on acceptance
- support for research data, including large and complex data types
- gold Open Access which fosters wider collaboration and increased citations
- maximum visibility for your research: over 100M website views per year

At BMC, research is always in progress.

Learn more biomedcentral.com/submissions

BMC

von Felten *et al. Trials* (2024) 25:568
https://doi.org/10.1186/s13063-024-08351-y

Trials

UPDATE

Open Access

Multicomponent family support intervention in intensive care units: statistical analysis plan for the cluster-randomized controlled FICUS trial

Stefanie von Felten[1*], Miodrag Filipovic[2], Marie-Madlen Jeitziner[3], Lotte Verweij[4,5], Marco Riguzzi[4,5†] and Rahel Naef[4,5†]

Abstract

The FICUS trial is a cluster-randomized superiority trial to determine the effectiveness of a nurse-led, interprofessional family support intervention (FSI) on the quality of care, family management and individual mental health of family members of critically ill patients, compared to usual care. This paper describes the statistical analysis plan of the FICUS trial. The primary outcome is quality of family care, assessed by the Family Satisfaction in ICU Questionnaire (FS-ICU-24R) at patient discharge from the ICU. Several secondary outcomes are additionally assessed 3, 6, and 12 months thereafter. Sixteen clusters (ICUs) were randomly assigned 1:1 to FSI or usual care using minimization (8 per treatment). The target sample size is 56 patients per cluster (896 in total). Recruitment has been completed in January 2024. The follow-up of the last participant will be completed in early 2025. The primary and secondary outcomes will be analyzed by linear mixed-effects models (LMM). The main model for the primary outcome will include a random intercept per cluster with treatment (FSI vs. usual care) as the only explanatory variable due to the relatively small number of clusters. In addition, covariate-adjusted analyses will be conducted, including two cluster-level characteristics used in the minimization as well as participant-level characteristics. Moreover, a number of subgroup analyses by cluster- and participant-level characteristics are pre-specified.

Trial registration ClinicalTrials.gov NCT05280691. Registered on February 20, 2022.

Keywords Statistical analysis plan, Cluster-randomized trial, Critical care, Complex intervention, Family care

Protocol version
This document is based on the study protocol of the FICUS trial version Version 1.1 (dated June 21, 2022). The study protocol was published in Trials [1].

†Marco Riguzzi and Rahel Naef are joint last authors.

*Correspondence:
Stefanie von Felten
stefanie.vonfelten@uzh.ch
[1] Department of Biostatistics at Epidemiology, Biostatistics and Prevention Institute, University of Zurich, Hirschengraben 84, Zurich CH-8001, Switzerland
[2] Division of Perioperative Intensive Care Medicine, Kantonsspital St. Gallen, Rorschacher Strasse 95, St. Gallen CH-9007, Switzerland
[3] Department of Intensive Care Medicine, Inselspital, University Hospital Bern, Freiburgstrasse 10, Bern CH-3010, Switzerland
[4] Institute for Implementation Science in Health Care, Faculty of Medicine, University of Zurich, Universitätstrasse 84, Zurich CH-8006, Switzerland
[5] Centre of Clinical Nursing Science, University Hospital Zurich, Rämistrasse 100, Zurich CH-8091, Switzerland

Introduction

Background and rationale for trial

The admission of a patient to an intensive care unit (ICU) creates enormous uncertainty and stress among family members [1–3]. Critical illness is life-altering and often life-threatening, which exposes families to extraordinary challenges for which they are often unprepared [4, 5]. A close other's critical illness is associated with emotional distress and negative mental health outcomes for families [6–9]. When affected by critical illness, families have specific needs, such as (1) being with their critically ill close other to provide meaningful support, (2) developing trustful partnerships with ICU staff to receive ongoing information on the patient's condition and prognosis

© The Author(s) 2024. **Open Access** This article is licensed under a Creative Commons Attribution 4.0 International License, which permits use, sharing, adaptation, distribution and reproduction in any medium or format, as long as you give appropriate credit to the original author(s) and the source, provide a link to the Creative Commons licence, and indicate if changes were made. The images or other third party material in this article are included in the article's Creative Commons licence, unless indicated otherwise in a credit line to the material. If material is not included in the article's Creative Commons licence and your intended use is not permitted by statutory regulation or exceeds the permitted use, you will need to obtain permission directly from the copyright holder. To view a copy of this licence, visit http://creativecommons.org/licenses/by/4.0/.

and ensure communication among involved parties, and (3) receiving guidance and support in dealing with the enormous challenges they are facing during and after the critical illness phase [4, 10–15]. Structured support for families is therefore called for [16–19].

Guidelines for family-centered care in the ICU recommend proactive engagement, communication and support to families, and the use of consultations and specific family navigator roles [20, 21], which has only been partially implemented to date [19, 20]. Moreover, there is insufficient empirical evidence on the clinical effectiveness of specific family support interventions that combine these different family care practices into a program of family care. Therefore, the Family support intervention in Intensive Care UnitS (FICUS) trial investigates the clinical effectiveness of a multi-component, nurse-led, interprofessional family support intervention (FSI) in addition to usual care [22].

Objectives

The primary objective is to show that the FSI, in addition to usual care, improves the quality of family care in ICU, assessed as family members' satisfaction with care at patient ICU discharge, compared to usual care alone. The secondary objective is to test the effectiveness of the FSI on further indicators of quality of care assessed by family members at ICU discharge, as well as on family management of critical illness and family members' post-ICU mental health, assessed at patient ICU admission (baseline), patient ICU discharge, and 3, 6, and 12 months following ICU discharge.

Study methods

Trial design

The FICUS trial is a parallel, cluster-randomized, controlled, multicenter, superiority trial with an equal number of clusters per study arm (8 ICUs each). The effectiveness-implementation hybrid type 1 design [23] examines, in addition to clinical effectiveness, the implementation of the FSI in the dynamic and complex "real-world" context of ICU care. However, this statistical analysis plan is focused on analyses of effectiveness. The intervention (FSI) consists of multiple components of family engagement, support, and communication provided by designated family nurses and members of the interprofessional team along the patient pathway, including follow-up care. The FSI is manual-based and includes (1) early engagement and liaison with families over time, (2) psycho-educational and relationship-focused family interventions in the form of therapeutic conversations, and (3) structured, interprofessional communication, and shared decision-making with families [22]. Families in the control arm receive usual care. While several members of a family receive the intervention, only one family member per patient is included in the study.

After the collection of baseline data (at patient admission to ICU, T0), outcomes are assessed at patient discharge from ICU (T1, primary outcome) as well as 3, 6, and 12 months thereafter (T2, T3, and T4, respectively), using established psychometric family-reported outcome measures. In addition, patient-related data are retrieved from clinical records at T0 and T1.

Randomization

Clusters (ICUs) were assigned 1:1 to the intervention or the control arm using minimization. The variables used in the minimization procedure are the certification of the ICU (2 groups) and hospital (one hospital included 2 ICUs and one hospital included 4 ICUs). We originally planned to define degree of specialization as specialized vs. general ICU [22] but finally used the ICU certification according to the registry of the certified training centers of the Swiss Institute for Continuing Medical Education (https://www.siwf-register.ch). The classification depends on the size (total number of ICU treatment days/mechanical ventilation hours), case mix, hospital infrastructure, and possibility for scientific activity and approximately divides hospitals into major teaching hospitals (A/Au, larger cantonal and university hospitals) vs. other teaching hospitals (B, smaller cantonal and regional hospitals). The minimization was prepared by SvF using the R package `Minirand` [24, 25]. A random seed was used to make the minimization procedure reproducible, which was generated by MR rolling a 20-sided die three times resulting in a six-digit number. To avoid the minimization process to be fully deterministic, we used a random component of 10%. Due to the relatively small number of clusters, we determined a priori that the whole randomization process including setting the seed would be repeated if (and only if) ICUs from the same hospital were not assigned 1:1 or the clusters overall were not assigned 1:1 (8 each to the intervention and control arm). SvF and MR performed the minimization on February 24, 2022.

Sample size

The sample size calculation is described in more detail in the published study protocol [22]. Using the R package `clusterPower` [26], we determined that with an average cluster size of 50 evaluable participants, a coefficient of variation in cluster size of 0.2, a difference in the primary outcome (FS-ICU-24 total score) between groups of 5.5, a within-group standard deviation of 16.3, and an ICC of 0.03, 8 clusters need to be assigned to each study arm (16 in total) to achieve a power of 80% at a significance level of 5%. To account for a drop-out rate of 10%

of participants within clusters (but no drop-out of entire clusters), an average number of 56 participants per cluster should be recruited.

Framework

The FICUS trial aims to demonstrate the superiority of the FSI in comparison to usual care in the ICUs for the primary outcome (assessed at T1 only). Secondary outcomes will also be compared using a superiority framework.

Statistical interim analyses and stopping guidance

No interim analyses are planned.

Timing of final analysis

The effectiveness of the FSI on the primary outcome and secondary outcomes only assessed once at patient discharge from the ICU (T1) will be analyzed after all study centers have completed recruitment (last participant-in) and all participants have completed the first follow-up data collection (T1). All other analyses of FSI effectiveness will be performed after completion of the trial, i.e., after all participants have reached 1 year of follow-up after patient discharge from ICU (T4).

Timing of outcome assessments

The primary outcome, family satisfaction with ICU care (FS-ICU-24R), as well as the secondary outcomes of quality of communication (QQPPI-14) and nurse support (ICE-FPSQ-14) are assessed at patient discharge from ICU only (T1). All other secondary outcomes concern family and mental health measures and are assessed at T1 as well as 3, 6, and 12 months thereafter (T2–T4). For the secondary outcomes assessed at multiple time points, an assessment at baseline is also performed within 4 days after admission to ICU (T0). The originally planned time windows for the outcome assessments were a maximum of 1 day before and up to 14 days after ICU discharge (T1), and 90, 180, and 365 days after T1 ± 14 days for T2–T4, respectively. However, to reduce the amount of missing data due to later return of questionnaires, these time windows were adapted as follows:

- T1—discharge from ICU (−1 day/+90 days, completed before T2 for quality of family care indicators; −1 day/+4 weeks for family and mental health indicators)
- T2—3 months following T1 (90 days; −14 days/+4 weeks)
- T3—6 months following T1 (180 days; −14 days/+6 weeks)
- T4—12 months following T1 (365 days; −14 days/+6 weeks)

The relatively large time window of +90 days for the quality of family care indicators was extended compared to the time window specified in the published study protocol [22] because of high burden in family members, which is likely to lead to a return of questionnaires later than the originally planned 2 weeks, but will hardly lead to a recall bias. It is important to note that study endpoints in the family and mental health indicators, which are measured at several time points and are likely to fluctuate over time, have a narrower time-window at T1 and also at T2–T4.

Statistical principles

Level of statistical significance

The significance level for the primary outcome and the secondary outcomes will be set at 0.05 (two-sided).

Adjustment for multiplicity

No adjustments for multiplicity are planned. Tests for the primary outcome will be considered confirmatory, whereas tests for the secondary outcomes and subgroup analyses will be considered exploratory. All planned analyses will be reported. All p-values will be reported with two significant digits if ≥ 0.0001, and as < 0.0001 otherwise.

Confidence intervals to be reported

We will report 95% confidence intervals for all point estimates.

Adherence and protocol deviations

The intervention is standardized in terms of components, intervention content, and a minimum dose along the clinical patient pathway (i.e., at least five intervention contacts/doses, representing all three intervention components of “engaging & liaising,” “supporting,” and “communicating”; see Fig. 3 in the published study protocol [22]). Interventionists tailor the frequency of intervention contacts and the dose of each intervention component according to a patient’s course of illness and/or a family’s preferences and needs, thereby potentially increasing dose and frequency.

Adherence is defined as fidelity to the manual-based study intervention (=fidelity consistency). Consistent intervention fidelity has been reached when the participant has received the protocolized minimum dose of the five intervention contacts, representing all three intervention components, within the specified time frame along the patient pathway [22]. Adherence is not defined under usual care (control). We will report the number and percentage of participants who did or did not receive the minimum dose of the FSI in the intervention arm.

Relevant deviations from the study protocol were defined as follows:

- If an enrolled participant is unable to complete the baseline questionnaire within the first 4 days after patient admission
- If an enrolled participant is unable to complete the quality of care questionnaires within the first 90 days after the patients' ICU discharge (end of time window for T1, see the "Timing of outcome assessments" section).
- If a declined patient general consent form for the use of clinical routine data for research is discovered after the family member has been enrolled into the trial, and the patient subsequently does not agree to the specific use of their routine clinical data for this trial, and
- If a patient's actual ICU stay lasted less than the expected 48 h

We will report the number and percentage of participants or patients with these protocol deviations by treatment (intervention and control). Regarding the return of quality of care questionnaires, we will additionally report the number and percentage of participants who returned the questionnaires later than 14 days but before 90 days, since 14 days were the originally planned end of the time window for T1 in the study protocol (see the "Timing of outcome assessments" section).

None of the violations will lead to the exclusion of the family member participant from the trial. However, some of the protocol deviations have consequences for the data collection and lead to missing data: (1) if participants return the baseline questionnaire after 4 days, only the baseline demographic data (that are not subject to change over time) will be recorded; (2) if participants return the quality of care questionnaires after 90 days, their answers will not be considered; (3) if patients decline the use of their routine clinical data, no patient data will be extracted and used in the analysis.

Analysis populations

We will adhere to the intention-to-treat principle at the cluster-level and participant-level as much as possible. Clusters and their participants will be analyzed by the treatment to which they were assigned. Should a patient be transferred to another cluster (other ICU), the family member participant will be analyzed in the original cluster. This will be possible due to individual informed consent. Our analysis population will thus consist of all participants who were included in the trial and who met the eligibility criteria. See the "Missing data" section for handling of missing data.

Trial population

Screening data

All family members who either (1) entered the study (2) were considered non-eligible, or (3) were eligible but not enrolled into the study, are documented in a screening log. Furthermore, the participation of each family member is documented in the enrollment log. At screening, sex and year of birth are collected for the patients and their family members as well as their reason for non-participation (eligibility criteria not met, not invited to participate, refused to participate, other reasons). We plan to report the number of screened patients and family members, with reasons for non-participation, because sex and year of birth do not provide sufficient information to evaluate the representativeness of the trial sample.

Eligibility

Cluster-level eligibility criteria

Study ICUs need to be able to provide the highest level of patient care and treat patients who are hemodynamically unstable, require ventilation with multiple-organ failure, and need multidisciplinary intervention. They may offer different or combined specialty care, including surgical, trauma, medical, cardiac, or neurological care. ICUs certified by the Swiss Society for Intensive Medicine (SGI) to operate at least eight beds are eligible. ICUs with fewer than 300 admissions per year of patients with a length of stay of 48 h or more in the ICU are excluded, as are ICUs with a preexisting, protocolized, interprofessional family support program.

Participant-level eligibility criteria

Participants are adult family members ($\geq$ 18 years of age) of critically ill persons who are admitted to an eligible ICU with no preexisting declined general informed consent for use of their clinical data for research purposes. Patient-level inclusion criteria are having an expected length of stay in ICU of $\geq$ 48 h, as appraised by the intaking ICU clinician (physician or nurse), and (1) a life-threatening condition with a high risk of death or long-lasting functional impairment or (2) a high risk of prolonged mechanical ventilation (> 24 h) as appraised by the intaking ICU clinician. Patient-level exclusion criteria are a pre-existing declined general consent or an actual ICU stay < 48 h. Family members are defined as close others from the patient's perspective, as noted in clinical records or advanced directives, or as legally defined surrogate decision-makers. Legal or blood kinship is not a requirement. They have to be a primary support person for the critically ill person, sign a written informed consent form, and be able to complete the baseline data collection and family-reported questionnaires in German within the required time frame. Family members of

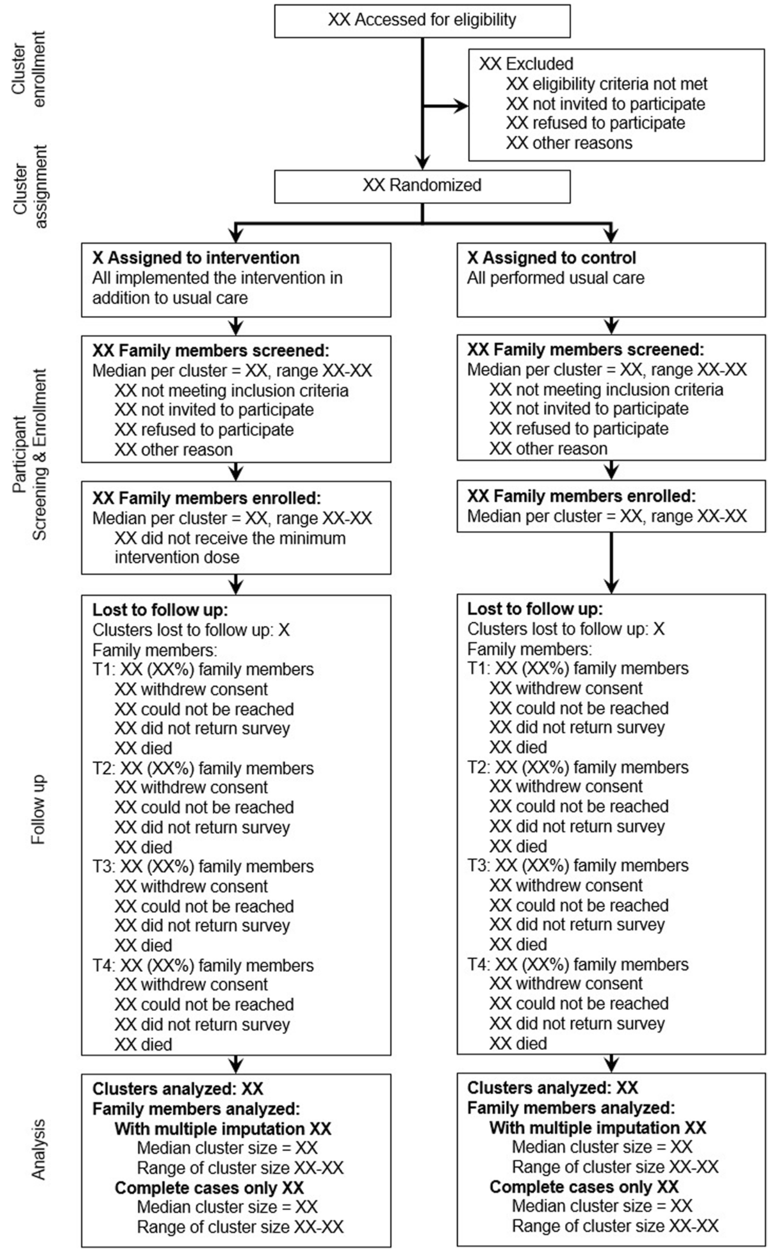

Fig. 1 CONSORT flow diagram for the FICUS trial

patients with refused general consent will not be invited to take part. Family members with prior inclusion in the FICUS trial in another ICU, with cognitive inability to understand the study or inability to complete the questionnaire as appraised by clinicians or study recruitment staff, will be excluded.

It should be noted that we do no longer plan to exclude family members of patients with an actual stay < 48 h on the study ICU after meeting the inclusion criteria of expected length of ICU stay of $\geq$ 48 h, even though this was originally defined as exclusion criterion. This is a protocol deviation defined in the "Adherence and protocol deviations" section.

CONSORT flow diagram

Figure 1 shows the CONSORT flow diagram we plan to report for the FICUS trial.

Table 1 Baseline characteristics of clusters before randomization

Characteristic	Intervention arm $n = 8$	Control arm $n = 8$
Certification[a] , *n* (%)		
Major teaching hospitals (A/Au)	XX (XX %)	XX (XX %)
Other teaching hospitals (B)	XX (XX %)	XX (XX %)
Operated ICU beds (annual average), median (min, max)	XX (XX, XX)	XX (XX, XX)
Patients admitted, median (min, max)	XX (XX, XX)	XX (XX, XX)
Treatment days, median (min, max)	XX (XX, XX)	XX (XX, XX)
High-risk admissions (SAPS-2 score[b] > 45), %, median (min, max)	XX (XX, XX)	XX (XX, XX)
Unplanned admissions, %, median (min, max)	XX (XX, XX)	XX (XX, XX)
SGI[c] classification of treatment shifts, %, median (min, max)		
Category 1a	XX (XX, XX)	XX (XX, XX)
Category 1b	XX (XX, XX)	XX (XX, XX)
Category 2	XX (XX, XX)	XX (XX, XX)
Category 3	XX (XX, XX)	XX (XX, XX)
Primary diagnosis/treatment at admission, %, median (min, max)		
Cardiac	XX (XX, XX)	XX (XX, XX)
Respiratory	XX (XX, XX)	XX (XX, XX)
Gastrointestinal	XX (XX, XX)	XX (XX, XX)
Neurological	XX (XX, XX)	XX (XX, XX)
Metabolic-endocrine	XX (XX, XX)	XX (XX, XX)
Trauma	XX (XX, XX)	XX (XX, XX)
Urogenital	XX (XX, XX)	XX (XX, XX)
Other	XX (XX, XX)	XX (XX, XX)
Mechanically ventilated patients, %, median (min, max)	XX (XX, XX)	XX (XX, XX)
Mechanically ventilated patients > 95 h, %, median (min, max)	XX (XX, XX)	XX (XX, XX)
Patients with > 1000 NEMS[d] points, %, median (min, max)	XX (XX, XX)	XX (XX, XX)
Length of stay (days), median (min, max)	XX (XX, XX)	XX (XX, XX)
Discharge destination, %, median (min, max)		
Other ICU	XX (XX, XX)	XX (XX, XX)
Intermediate care	XX (XX, XX)	XX (XX, XX)
General ward	XX (XX, XX)	XX (XX, XX)
Died	XX (XX, XX)	XX (XX, XX)
Other (rehabilitation or other care institution, home)	XX (XX, XX)	XX (XX, XX)
Nurse staffing (FTE/operated beds), %, median (min, max)	XX (XX, XX)	XX (XX, XX)
Staff with ICU certification, %, median (min, max)		
Nurses	XX (XX, XX)	XX (XX, XX)
Physicians	XX (XX, XX)	XX (XX, XX)
Family-centered care in ICU score[e] , median (min, max)	XX (XX, XX)	XX (XX, XX)
Patient & family-centered care score[f], median (min, max)	XX (XX, XX)	XX (XX, XX)

[a] Swiss Institute for Continuing Medical Education, classification see https://www.sgi-ssmi.ch/files/Dateiverwaltung/de/ressorts/quali/KDS%20Kommission%20Datensatz/SGI-Kat_20060309_d_2012.pdf

[b] Simplified Acute Physiology Score 2

[c] Swiss Society for Intensive Medicine

[d] Nine Equivalents of Nursing Manpower use Score

[e] Mean score (range 1–4, where 1 indicates the highest degree of family-centeredness) of 22 selected items from the gap analysis tool provided by the Society of Critical Care Medicine

[f] Mean score (range 1–5) of 30 selected items from the Patient- and Family-Centered Care Organizational Self-Assessment Tool

Withdrawal/follow-up

Withdrawal of consent by the study participants leads to the termination of their data collection. The data collected up to that point in time will be used in the analysis. If a patient withdraws consent for the use of clinical data for the study, no new clinical patient data will

Table 2 Baseline characteristics of patients upon admission to ICU

Patient characteristic	Intervention arm *n* = XX	Control arm *n* = XX
Age (years), median (q1, q3)	XX (XX, XX)	XX (XX, XX)
Sex, n (%)		
Female	XX (XX %)	XX (XX %)
Male	XX (XX %)	XX (XX %)
Other	XX (XX %)	XX (XX %)
Civil status, *n* (%)		
Single	XX (XX %)	XX (XX %)
Married/in (registered) partnership	XX (XX %)	XX (XX %)
Divorced/separated	XX (XX %)	XX (XX %)
Widowed/surviving partner	XX (XX %)	XX (XX %)
Unplanned admission to ICU, *n* (%)	XX (XX %)	XX (XX %)
Admitted from, *n* (%)		
Emergency room	XX (XX %)	XX (XX %)
Operating room	XX (XX %)	XX (XX %)
General ward	XX (XX %)	XX (XX %)
Intermediate care	XX (XX %)	XX (XX %)
Other ICU	XX (XX %)	XX (XX %)
Other institution (rehabilitation, nursing home)	XX (XX %)	XX (XX %)
Mechanical ventilation, *n* (%)	XX (XX %)	XX (XX %)
Mechanical circulatory support, *n* (%)	XX (XX %)	XX (XX %)
SAPS-2[a] score, median (q1, q3)	XX (XX, XX)	XX (XX, XX)
NEMS[b] score, median (q1, q3)	XX (XX, XX)	XX (XX, XX)
SOFA[c] score, median (q1, q3)	XX (XX, XX)	XX (XX, XX)
Trauma treatment, *n* (%)	XX (XX %)	XX (XX %)
AIS[d] score in case of trauma treatment, median (q1, q3)	XX (XX, XX)	XX (XX, XX)
Surgery, *n* (%)		
Planned	XX (XX %)	XX (XX %)
Emergency	XX (XX %)	XX (XX %)
No surgery	XX (XX %)	XX (XX %)
Previous ICU-treatment within last 3 months, *n* (%)	XX (XX %)	XX (XX %)

[a] Simplified Acute Physiology Score 2

[b] Nine Equivalents of Nursing Manpower use Score

[c] Sequential Organ Failure Assessment Score

[d] Abbreviated Injury Scale Score

be collected. We will report the number and percentage of participants who withdraw informed consent and of patients who withdraw consent for their use of clinical data by study arm. In addition, we will report the number and percentage of participants who are lost to follow-up. We will further split these numbers by the timing of withdrawal/follow-up, e.g., before visit T1, T2, T3, or T4.

Baseline characteristics

Baseline characteristics of the clusters (ICUs) before randomization will be summarized as outlined in Table 1 based on the most recent Minimal Data Set (MDSi) which includes data from the last calendar year before the start of the trial. Baseline characteristics of the patients and family member participants will be summarized as shown in Tables 2 and 3. Due to the relatively small number of clusters per arm, we will report medians together with minima and maxima for the cluster characteristics where appropriate (Table 1) but medians together with first and the third quartiles (q1 and q3) for the patient and family member characteristics (Tables 2 and 3).

Analysis

Outcome definitions

The primary outcome is the quality of family care in the ICU, operationalized as family satisfaction with ICU care, which is an established core indicator of the quality of family care. It will be assessed at discharge from ICU by

Table 3 Baseline characteristics of family members participating in the study

Family member characteristic	Intervention arm *n* = XX	Control arm *n* = XX
Age (years), median (q1, q3)	XX (XX, XX)	XX (XX, XX)
Sex, *n* (%)		
Female	XX (XX %)	XX (XX %)
Male	XX (XX %)	XX (XX %)
Other	XX (XX %)	XX (XX %)
Civil status, *n* (%)		
Single	XX (XX %)	XX (XX %)
Married/in (registered) partnership	XX (XX %)	XX (XX %)
Divorced/separated	XX (XX %)	XX (XX %)
Widowed/surviving partner	XX (XX %)	XX (XX %)
Occupational status, *n* (%)		
Employed (full- or part-time)	XX (XX %)	XX (XX %)
Retired	XX (XX %)	XX (XX %)
Student	XX (XX %)	XX (XX %)
Unemployed	XX (XX %)	XX (XX %)
Type of family member, *n* (%)		
Partner/spouse	XX (XX %)	XX (XX %)
Parent	XX (XX %)	XX (XX %)
Child	XX (XX %)	XX (XX %)
Other	XX (XX %)	XX (XX %)
Co-habiting with patient, *n* (%)	XX (XX %)	XX (XX %)
Travel time to hospital (minutes), median (q1, q3)	XX (XX, XX)	XX (XX, XX)
Self-perceived health (VAS[a], 0-100), median (q1, q3)	XX (XX, XX)	XX (XX, XX)
Past/current psychiatric or psychological treatment, *n* (%)	XX (XX %)	XX (XX %)
Current use of prescription drugs, *n* (%)	XX (XX %)	XX (XX %)
Current treatment for chronic illness, *n* (%)	XX (XX %)	XX (XX %)
Prior ICU experience, *n* (%)		
As patient	XX (XX %)	XX (XX %)
As family member of patient	XX (XX %)	XX (XX %)
Both	XX (XX %)	XX (XX %)
None	XX (XX %)	XX (XX %)

[a] Visual analog scale

the Family Satisfaction in ICU Questionnaire (FS-ICU-24R) [27, 28].

Secondary outcomes are shown together with the primary outcome in Table 4 (adapted from [22], where more detail is given), which also provides the range of each outcome, Cronbach's α (where applicable), and the timing of each assessment.

Analysis methods

Primary outcome—main analysis

The FS-ICU-24-R total score at patient discharge from ICU will be analyzed by a linear mixed-effects model (LMM) with a random intercept per cluster to account for the non-independence of family members from the same cluster. Due to the small number of clusters, the main model (model 1) will include the treatment (intervention vs. control) as the only explanatory variable, and the Satterthwaite approximation for the denominator degrees of freedom will be used, as was recommended [30] for cluster-randomized trials with 10–20 clusters randomized. The ICC will be estimated from this model based on the residual variance and between-cluster variance.

Primary outcome—sensitivity analyses

The following covariate-adjusted sensitivity analyses will be conducted to adjust the treatment effect estimate for potential confounding: At the cluster level, the ICU certification (as used in the cluster randomization), overall ICU nurse staffing (ratio of nurses full-time equivalents

Table 4 Primary and secondary outcomes measures

Domain/construct	Measure[a]	Range	α[b]	T0	T1	T2–T4
Quality of family care						
Satisfaction with care (primary outcome)	Family satisfaction with ICU Care (FS-ICU-24R)	0–100	0.96[c]		X	
Subscales of FS-ICU-24R	FS-ICU-24R Care (subscale) score	0–100	0.95[d]		X	
	FS-ICU-24R Decision-Making (subscale) score	0–100	0.87[d]		X	
Quality of communication	Questionnaire on Quality of Physician–Patient Interaction (QQPPI-14)	1–5	0.95		X	
Support from nurses	Family Perceived Support Questionnaire (ICE-FPSQ-14)	14–70	0.92[e]		X	
Family management						
Family functioning	Family Assessment Device - General Functioning Scale (FAD-GF-12)	1–4	0.87	X	X	X
Family resilience	Brief Resilience Scale (BRS-6)[f]	1–5	0.85	X	X	X
Mental health						
Subjective well-being	Satisfaction with Life Scale (SWLS-5)	5–35	0.89–0.92	X	X	X
	WHO-5 Well-Being Index (WHO-5)	0–100	0.92	X	X	X
	Adapted VAS on Quality of Life (QoL-VAS)	0–100	n/a	X	X	X
Psychological distress	Distress Thermometer (DT)	0–10	n/a	X	X	X
	Impact of Events Scale-6 (IES-6)	0–4	0.80	X	X	X
	Hospital Anxiety and Depression Scale (HADS-14)	0–21	> 0.80	X	X	X

[a] German versions of measures

[b] Cronbach's α, most references are listed in the study protocol [22], except for some updates

[c] Cronbach's α according to [27], for the English version

[d] Cronbach's α according to [28]

[e] Cronbach's α according to [29]

to the number of certified ICU beds), and the family-centered care in ICU score (mean score of 22 items from the Standardized score sheet provided by the Society of Critical Care Medicine [31], ranging from 1 to 4) will each be added separately to the main model described above (models 2–4). The hospital, also used in the cluster randomization, will not be accounted for, neither as a covariate nor as a random term, because most ICUs are in a different hospital (see the "Randomization" section).

At the individual participant level, patient age, cause of admission (unplanned or planned), the SAPS-2 score of the patient (mortality risk), type of relationship between patient and family member (partner, child or other), and the family member's previous ICU experience will be added together to the main model described above (model 5) and to the cluster-level adjusted models (models 6–8).

To assess the sensitivity of the results with regard to missing data (see also the "Missing data" section), we will apply model 1 and model 5 as described above to a multiply imputed data set (models 9 and 10). We may further apply models 6–8 to the multiply imputed data set.

Primary outcome—subgroup analyses

Subgroup analyses are planned regarding the primary outcome for the following baseline characteristics:

Cluster characteristics

- Certification (as defined in the "Randomization" section)
- Overall ICU nurse staffing
- Family-centered care in ICU score (mean score, range 1–4)

Patient or family member characteristics

- Patient age
- Patient sex
- Patient's cause of admission (unplanned vs. planned)
- Mortality risk of patient, as assessed by the SAPS-2 score
- Type of relationship between patient and family member (partner, child, other)
- Family member's prior ICU experience (yes/no)
- Family resilience, as assessed by the Brief Resilience Scale (BRS-6)
- Family functioning, as assessed by the Family Assessment Device - General Functioning Scale (FAD-GF-12)
- Family member's anxiety score assessed by the Hospital Anxiety and Depression Scale (HADS)
- Family member's depression score assessed by HADS

Subgroup effects will be tested by a separate LMM fitted to the primary outcome for each subgroup variable, adding the subgroup variable and its interaction with the treatment as explanatory variables to the main model described above. A significant interaction would indicate a different treatment effect depending on the subgroup (or along a gradient for the continuous subgroup variables).

Primary outcome—additional analyses

To investigate the effect of intervention fidelity, three models that each include a certain type of intervention fidelity will be fitted to the primary outcome. The explanatory variable treatment (intervention vs. control) in model 5 will be replaced by one of the following explanatory variables:

- Consistency of intervention delivery with three levels: consistent delivery in the intervention arm, inconsistent delivery in the intervention arm and usual care in the control arm. Intervention delivery is defined as consistent if the minimal intervention contact dose according to protocol was provided (see the "Adherence and protocol deviations" section)
- Volume (total duration of interventions (conversations) divided by patient length of stay at ICU), which is zero in the control arm
- Frequency (total number of interventions (conversations) divided by patient length of stay at ICU), which is zero in the control arm

These models will also be applied to the multiply imputed data set.

Secondary outcomes

The secondary outcomes regarding the quality of care, which are only measured once at discharge from ICU (T1), will be analyzed with an LMM as described above for the primary outcome. All other secondary outcomes, which are measured at baseline (T0) and four times after the start of the intervention (T1–T4), will be analyzed by an LMM with a random intercept per cluster and a random intercept per family member (nested within clusters) to additionally account for the non-independence of repeated measurements from the same study participant. The serial autocorrelation of residuals will be modeled using a first-order autoregressive correlation structure. The models will include the treatment (intervention vs. control), the corresponding baseline measurement, the visit, and the visit × treatment interaction to assess whether the treatment effect changes over time.

Depending on the results of the sensitivity analyses for the primary outcome, we will use the same covariate-adjusted sensitivity analyses (or some of them) also for the secondary outcomes.

Presentation of outcome data and effect size estimates

Outcome data will be presented in a table containing descriptive statistics of each outcome per trial arm as well as point estimates and 95% confidence intervals for treatment effect estimates and corresponding p-values.

Missing data

We will analyze complete cases in the main analysis and use multilevel multiple imputation of missing data (outcomes and covariates) at the participant level ([32], Chapter7) in sensitivity analyses (the "Analysis methods" section). We do not expect any missing cluster level covariates. We will separately impute missing values in the control and intervention arm, as recommended by [33]. The dataset used for multiple imputation will be a subset of the full data set, containing (1) the primary outcome, (2) all cluster and participant level covariates that are used in the planned analyses of the primary outcome (see the "Analysis methods" section), (3) secondary quality of care outcomes (only measured at T1) due to an expected correlation with primary outcome, and (4) once known which covariates contain missing data, additional variables that are correlated with the missing covariate data. The number of imputations per missing value will be determined based on the fraction of missing information [34]. We do not expect any clusters to be missing as a whole, but would exclude them from the analysis if present.

Additional analyses

Subgroup analyses as defined for the primary outcome (see the "Analysis methods" section) will be performed for each of these secondary outcomes:

- Quality of communication Questionnaire on Quality of Physician-Patient Interaction (QQPPI-14), T1
- Impact of Events Scale-6 (IES-6), T1-T4
- Hospital Anxiety and Depression Scale (HADS-14): Anxiety Subscale, T1-T4
- Hospital Anxiety and Depression Scale (HADS-14): Depression Subscale, T1-T4

Additional analyses, similar to those defined for the primary outcome (see the "Analysis methods" section), will be performed for all secondary outcomes.

Due to the repeated measurements for some of these secondary outcomes, the corresponding models will

additionally include a random intercept per family member and the corresponding baseline measurement per variable as covariate (as described in the "Analysis methods" section) but will not include the visit and the visit × treatment interaction. Should the analyses of secondary outcomes (the "Analysis methods" section) reveal that an intervention effect only manifests at certain follow-up times, we would focus the described additional analyses of secondary outcomes on these follow-up times.

In addition, we will compute Cronbach's alpha for total scores and subscales of all family-reported outcome measures used in our study.

Harms

The intervention applied in this trial cannot cause any harm to the patient, as it is applied to the family members of the patient and is a family care intervention, not a medical intervention. However, we will report all SAEs, for example if a study participant (family member) dies during the follow-up period and is thus lost to follow-up.

Statistical software

All statistical analyses will be performed using the R system for statistical computing and graphics [24] (current version at the time of analysis). The linear mixed-effects models will be fitted using the `lmer` function from the R package `lme4` [35] in combination with the package `lmertest` which implements the Satterthwaite method for the degrees of freedom [36]. Multiple imputation will be performed using the R package `mice` [37] in combination with the `2l.pmm` method from the `miceadds` package for the multilevel imputation [38]. All software used will be reported together with the results of the analyses.

Acknowledgements
None.

Authors' contributions
SvF designed the statistical analysis plan (SAP) for the funding application and study protocol. The detailed SAP presented here was refined with contributions from all other co-authors. The roles and responsibility of all co-authors are as follows. *Principal investigator*: Prof. Dr. phil. Rahel Naef, Institute for Implementation Science in Health Care, Faculty of Medicine, University of Zurich & Center of Clinical Nursing Science, University Hospital Zurich, Switzerland. *Co-investigators*: Prof. Dr. med. Miodrag Filipovic, Division of Perioperative Intensive Care Medicine, Kantonsspital St. Gallen, Switzerland. Dr. phil. Marie-Madlen Jeitziner, Department of Intensive Care Medicine, Inselspital, University Hospital Bern, Switzerland. *Trial statistician*: Dr. Stefanie von Felten, Department of Biostatistics at Epidemiology, Biostatistics and Prevention Institute, University of Zurich, Switzerland. *Senior researchers*: Dr. Marco Riguzzi, Institute for Implementation Science in Health Care, Faculty of Medicine, University of Zurich & Center of Clinical Nursing Science, University Hospital Zurich, Switzerland. Dr. Lotte Verweij, Institute for Implementation Science in Health Care, Faculty of Medicine, University of Zurich & Center of Clinical Nursing Science, University Hospital Zurich, Switzerland.

Funding
The FICUS trial is funded by the Swiss National Science Foundation's Investigator Initiated Clinical Trials (IICT) program, grant no. 198778, to RN, MF, and MJ.

Availability of data and materials
Data sharing is not applicable to this article as no datasets were generated or analyzed during the current study (statistical analysis plan).

Declarations

Ethics approval and consent to participate
Not applicable.

Consent for publication
Not applicable.

Competing interests
The authors declare that they have no competing interests.

Received: 7 February 2024 Accepted: 22 July 2024
Published online: 28 August 2024

References

1. Alfheim HB, Rosseland LA, Hofsø K, Småstuen MC, Rustøen T. Multiple symptoms in family caregivers of intensive care unit patients. J Pain Symptom Manag. 2018;55(2):387–94. https://doi.org/10.1016/j.jpainsymman.2017.08.018.
2. Minton C, Batten L, Huntington A. A multicase study of prolonged critical illness in the intensive care unit: families' experiences. Intens Crit Care Nur. 2019;50:21–7. https://doi.org/10.1016/j.iccn.2018.08.010.
3. Turner-Cobb JM, Smith PC, Ramchandani P, Begen FM, Padkin A. The acute psychobiological impact of the intensive care experience on relatives. Psychol Health Med. 2016;21(1):20–6. https://doi.org/10.1080/13548506.2014.997763.
4. Hayes K, Harding S, Buckley K, Blackwood B, Latour JM. Exploring the experiences of family members when a patient is admitted to the ICU with a severe traumatic brain injury: a scoping review. J Clin Med. 2023;12(13):4197. https://doi.org/10.3390/jcm12134197.
5. Saeid Y, Moradian ST, Ebadi A, Salaree MM. The family intensive care unit syndrome: a qualitative content analysis. Nurs Crit Care. 2021;27(3):401–9. https://doi.org/10.1111/nicc.12683.
6. Metzger K, Gamp M, Tondorf T, Hochstrasser S, Becker C, Luescher T, et al. Depression and anxiety in relatives of out-of-hospital cardiac arrest patients: results of a prospective observational study. J Crit Care. 2019;51:57–63. https://doi.org/10.1016/j.jcrc.2019.01.026.
7. Białek K, Sadowski M. Stress, anxiety, depression and basic hope in family members of patients hospitalised in intensive care units - preliminary report [Journal Article]. Anaesthesiol Intensive Ther. 2021;53(2):134–40. https://doi.org/10.5114/ait.2021.105728.
8. Abdul Halain A, Tang LY, Chong MC, Ibrahim NA, Abdullah KL. Psychological distress among the family members of Intensive Care Unit (ICU) patients: a scoping review. J Clin Nurs. 2022;31(5–6):497–507. https://doi.org/10.1111/jocn.15962.
9. Gurbuz H, Demir N. Anxiety and depression symptoms of family members of intensive care unit patients: a prospective observational study and the lived experiences of the family members. Avicenna J Med. 2023;13(2):89–96. https://doi.org/10.1055/s-0043-1769933.
10. Damanik SRH, Chen HM. Family needs among patients hospitalized in critical care unit: scoping review. Enfermería Clín. 2021;31:541–8. https://doi.org/10.1016/j.enfcli.2021.09.001.
11. Kynoch K, Ramis MA, McArdle A. Experiences and needs of families with a relative admitted to an adult intensive care unit: a systematic review of qualitative studies. JBI Evid Synth. 2021;19(7):1499–554. https://doi.org/10.11124/jbisrir-2016-003193.
12. Imanipour M, Kiwanuka F, Akhavan Rad S, Masaba R, Alemayehu YH. Family members' experiences in adult intensive care units: a systematic

review. Scand J Caring Sci. 2019;33(3):569–81. https://doi.org/10.1111/scs.12675.
13. Scott P, Thomson P, Shepherd A. Families of patients in ICU: a scoping review of their needs and satisfaction with care. Nurs Open. 2019;6(3):698–712. https://doi.org/10.1002/nop2.287.
14. Dijkstra BM, Felten-Barentsz KM, van der Valk MJM, Pelgrim T, van der Hoeven HG, Schoonhoven L, et al. Family participation in essential care activities: needs, perceptions, preferences, and capacities of intensive care unit patients, relatives, and healthcare providers-an integrative review. Aust Crit Care. 2022;36(3):401–19. https://doi.org/10.1016/j.aucc.2022.02.003.
15. Millward K, McGraw C, Aitken LM. The expressed support needs of families of adults who have survived critical illness: a thematic synthesis. Int J Nurs Stud. 2021;122:104048. https://doi.org/10.1016/j.ijnurstu.2021.104048.
16. Zante B, Camenisch SA, Schefold JC. Interventions in post-intensive care syndrome-family: a systematic literature review. Crit Care Med. 2020;48(9):e835–40. https://doi.org/10.1097/ccm.0000000000004450.
17. Kiwanuka F, Sak-Dankosky N, Alemayehu YH, Nanyonga RC, Kvist T. The evidence base of nurse-led family interventions for improving family outcomes in adult critical care settings: a mixed method systematic review. Int J Nurs Stud. 2022;125:104100. https://doi.org/10.1016/j.ijnurstu.2021.104100.
18. Rhoads SL, Trikalinos TA, Levy MM, Amass T. Intensive care based interventions to reduce family member stress disorders: a systematic review of the literature. J Crit Care Med (Targu Mures). 2022;8(3):145–55. https://doi.org/10.2478/jccm-2022-0014.
19. Goldfarb M, Alviar C, Berg D, Katz J, Lee R, Liu S, et al. Family engagement in the adult cardiac intensive care unit: a survey of family engagement practices in the Cardiac Critical Care Trials Network. Circ Cardiovasc Qual Outcome. 2023;16(9):e010084. https://doi.org/10.1161/circoutcomes.123.010084.
20. Davidson JE, Aslakson RA, Long AC, Puntillo KA, Kross EK, Hart J, et al. Guidelines for family-centered care in the neonatal, pediatric, and adult ICU. Crit Care Med. 2017;45(1):103–28. https://doi.org/10.1097/ccm.0000000000002169.
21. Ludmir J, Netzer G. Family-centered care in the ontensive care unit-what does best practice tell us? Semin Respir Crit Care Med. 2019;40(5):648–54. https://doi.org/10.1055/s-0039-1697957.
22. Naef R, Filipovic M, Jeitziner MM, von Felten S, Safford J, Riguzzi M, et al. A multicomponent family support intervention in intensive care units: study protocol for a multicenter cluster-randomized trial (FICUS Trial). Trials. 2022;23(1):533. https://doi.org/10.1186/s13063-022-06454-y.
23. Curran GM, Landes SJ, McBain SA, Pyne JM, Smith JD, Fernandez ME, et al. Reflections on 10 years of effectiveness-implementation hybrid studies. Front Health Serv. 2022;2:125. https://doi.org/10.3389/frhs.2022.1053496.
24. R Core Team. R: a language and environment for statistical computing. Vienna, Austria; 2022. https://www.R-project.org/. Accessed 15 Aug 2024.
25. Jin M, Polis A, Hartzel J. Minirand: minimization randomization. 2020. R package version 0.1.3. https://CRAN.R-project.org/package=Minirand. Accessed 15 Aug 2024.
26. Kleinman K, Moyer J, Reich N. clusterPower: power calculations for cluster-randomized and cluster-randomized crossover trials. 2017. R package version 0.6.111. https://CRAN.R-project.org/package=clusterPower. Accessed 15 Aug 2024.
27. Harrison D, Ferrando-Vivas P, Wright S, McColl E, Rowan K, Investigators FS. Psychometric assessment of the family satisfaction in the intensive care unit (FS-ICU-24) questionnaire among family members of patients admitted to adult general ICUs in the United Kingdom. Intensive Care Med Exp. 2015;3:1–2. https://doi.org/10.1186/2197-425X-3-S1-A152.
28. Stricker KH, Niemann S, Bugnon S, Wurz J, Rohrer O, Rothen HU. Family satisfaction in the intensive care unit: cross-cultural adaptation of a questionnaire. J Crit Care. 2007;22(3):204–11. https://doi.org/10.1016/j.jcrc.2006.12.008.
29. Freudiger K, Verweij L, Naef R. Translation and psychometric validation of the German version of the Iceland-Family Perceived Support Questionnaire (ICE-FPSQ): a cross-sectional study. J Fam Nurs. 2024;30(2):114–26. https://doi.org/10.1177/10748407241234262.
30. Leyrat C, Morgan KE, Leurent B, Kahan BC. Cluster randomized trials with a small number of clusters: which analyses should be used? Int J Epidemiol. 2018;47(1):321–31. https://doi.org/10.1093/ije/dyx169.
31. Hwang DY, El-Kareh R, Davidson JE. Implementing intensive care unit family-centered care: resources to identify and address gaps. AACN Adv Crit Care. 2017;28(2):148–54. https://doi.org/10.4037/aacnacc2017636.
32. van Buuren S. Flexible imputation of missing data. Chapman and Hall/CRC; 2018. https://stefvanbuuren.name/fimd/. Accessed 15 Aug 2024.
33. Sullivan TR, White IR, Salter AB, Ryan P, Lee KJ. Should multiple imputation be the method of choice for handling missing data in randomized trials? Stat Methods Med Res. 2018;27(9):2610–26. https://doi.org/10.1177/0962280216683570.
34. Madley-Dowd P, Hughes R, Tilling K, Heron J. The proportion of missing data should not be used to guide decisions on multiple imputation. J Clin Epidemiol. 2019;110:63–73. https://doi.org/10.1016/j.jclinepi.2019.02.016.
35. Bates D, Mächler M, Bolker B, Walker S. Fitting linear mixed-effects models using lme4. J Stat Soft. 2015;67(1):1–48. https://doi.org/10.18637/jss.v067.i01.
36. Kuznetsova A, Brockhoff PB, Christensen RHB. lmerTest package: tests in linear mixed effects models. J Stat Soft. 2017;82(13):1–26. https://doi.org/10.18637/jss.v082.i13.
37. van Buuren S, Groothuis-Oudshoorn K. mice: multivariate imputation by chained equations in R. J Stat Soft. 2011;45(3):1–67. https://doi.org/10.18637/jss.v045.i03.
38. Robitzsch A, Grund S. miceadds: some additional multiple imputation functions, especially for 'mice'; 2024. R package version 3.17-44. https://CRAN.R-project.org/package=miceadds. Accessed 15 Aug 2024.

Publisher's Note

Springer Nature remains neutral with regard to jurisdictional claims in published maps and institutional affiliations.
