# Supplementary material for: Nurse-Led Family Support Intervention for Families of Critically Ill Patients: The FICUS Cluster Randomized Clinical Trial
Source: JAMA Intern Med. 2025 Jul 28;185(9):1138–49. doi: 10.1001/jamainternmed.2025.3406 (PMC12558129; doi:10.1001/jamainternmed.2025.3406)
Supplement: Supplement 2. — eTable 1. Characteristics of intervention contacts eTable 2. Characteristics of intervention conacts with referrals eTable 3. Coefficient estimates from the sensitivity analysis (model 2) eTable 4. Coefficient estimates from the sensitivity analysis (model 3) eTable 5. Coefficient estimates from the sensitivity analysis (model 4) eTable 6. Coefficient estimates from the sensitivity analysis (model 5) eTable 7. Coefficient estimates from the sensitivity analysis (model 6) eTable 8. Coefficient estimates from the sensitivity analysis (model 7) eTable 9. Coefficient estimates from the sensitivity analysis (model 8) eTable 10. Coefficient estimates from the sensitivity analysis (model 9) eTable 11. Coefficient estimates from the sensitivity analysis (model 10) eTable 12. Coefficient estimates from the post hoc sensitivity analysis on excluding participants QoC questionnaire return later than 14 days after T1 eTable 13. Effect size estimate for the effect of the Intervention vs. Control on the primary outcome eTable 14. Coefficient estimates for the subgroup analysis on the primary outcome eTable 15. Coefficient estimates for the subgroup analysis on the secondary outcome QQPPI with the family-centered care eTable 16. Coefficient estimates from the additional analysis of the primary outcome eTable 17. Coefficient estimates from the additional analysis of the secondary outcome quality of communication eTable 18. Coefficient estimates from the additional analysis of the secondary outcome nurse support eTable 19. Minimal Clinically Important Difference (MCID) for all outcomes eTable 20. Intraclass correlation coefficients (ICC) for the primary outcome eFigure 1. Family support intervention eFigure 2. Distribution of the time to return of the QoC survey questionnaires eFigure 3. Forest plot of treatment effect estimates on the primary outcome eFigure 4. Forest plot of treatment effect estimates on the primary outcome eFigure 5. Diagnostic plots for the residuals from model 1 [file jamainternmed-e253406-s002.pdf]

Supplemental Online Content

Naef R, Jeitziner M-M, Riguzzi M, et al; the FICUS Study Group. Nurse-led family support intervention to for families of critically ill patients: the FICUS cluster randomized clinical trial. *JAMA Intern Med*. doi:10.1001/jamainternmed.2025.3406

eTable 1. Characteristics of intervention contacts

eTable 2. Characteristics of intervention contacts with referrals

eTable 3. Coefficient estimates from the sensitivity analysis (model 2)

eTable 4. Coefficient estimates from the sensitivity analysis (model 3)

eTable 5. Coefficient estimates from the sensitivity analysis (model 4)

eTable 6. Coefficient estimates from the sensitivity analysis (model 5)

eTable 7. Coefficient estimates from the sensitivity analysis (model 6)

eTable 8. Coefficient estimates from the sensitivity analysis (model 7)

eTable 9. Coefficient estimates from the sensitivity analysis (model 8)

eTable 10. Coefficient estimates from the sensitivity analysis (model 9)

eTable 11. Coefficient estimates from the sensitivity analysis (model 10)

eTable 12. Coefficient estimates from the post hoc sensitivity analysis on excluding participants QoC questionnaire return later than 14 days after T1

eTable 13. Coefficient estimates from the post hoc sensitivity analysis adjusting for time to return QoC survey questionnaire

eTable 14. Coefficient estimates for the subgroup analysis on the primary outcome

eTable 15. Coefficient estimates for the subgroup analysis on the secondary outcome *QQPPI* with the family-centered care

eTable 16. Coefficient estimates from the additional analysis of the primary outcome

eTable 17. Coefficient estimates from the additional analysis of the secondary outcome *quality of communication*

eTable 18. Coefficient estimates from the additional analysis of the secondary outcome *nurse support*

eTable 19. Minimal Clinically Important Difference (MCID) for all outcomes

eTable 20. Intraclass correlation coefficients (ICC) for the primary outcome

eFigure 1. Family support intervention

eFigure 2. Distribution of the time to return of the QoC survey questionnaires

eFigure 3. Forest plot of treatment effect estimates on the primary outcome

eFigure 4. Forest plot of treatment effect estimates on the primary outcome

eFigure 5. Diagnostic plots for the residuals from model 1 applied to the primary outcome

eFigure 6. Diagnostic plots for the residuals from model 5 applied to the primary outcome

eFigure 7. Violin plots showing the distribution of the primary outcome by treatment

eReferences

This supplemental material has been provided by the authors to give readers additional information about their work.

© 2025 Naef R. et al; the FICUS Study Group. *JAMA Internal Medicine*.

Supplementary material

A family support intervention to improve quality of care for families of critically ill patients: The FICUS cluster randomized trial

April 24, 2025

Naef et al., on behalf of the FICUS study group

Contents

1 Intervention characteristics 3

2 Sensitivity analyses of the primary outcome family satisfaction with ICU care (FS-ICU-24R) 6

3 Distribution of time to return of quality of care (QoC) questionnaires 10

4 *Post-hoc* sensitivity analyses of the primary outcome with regard to time to return of (QoC) questionnaires 11

5 Subgroup analyses of the primary outcome 12

6 Subgroup analyses of the secondary outcome quality of communication (QQPPI) 14

7 Distribution of the primary outcome and diagnostic plots of the residuals 14

8 Analyses investigating intervention fidelity (consistency) 18

9 Minimal Clinically Important Difference (MCID) for all outcomes 20

10 Intraclass correlation coefficients 20

11 References 20

Acronyms 21

© 2025 Naef R. et al; the FICUS Study Group. JAMA Internal Medicine.

List of eTables

1 Characteristics of intervention contacts ... 4
2 Characteristics of intervention contacts with referrals ... 5
3 Coefficient estimates from the sensitivity analysis adjusting for certification of the cluster. 6
4 Coefficient estimates from the sensitivity analysis adjusting for nurse staffing of the cluster. 6
5 Coefficient estimates from the sensitivity analysis adjusting for family-centered care in ICU score of the cluster ... 7
6 Coefficient estimates from the sensitivity analysis adjusting for several patient-level and family member-level covariates ... 7
7 Coefficient estimates from the sensitivity analysis adjusting for the certification of the cluster as well as several patient-level and family member-level covariates ... 7
8 Coefficient estimates from the sensitivity analysis adjusting for nurse staffing of the cluster as well as several patient-level and family member-level covariates ... 8
9 Coefficient estimates from the sensitivity analysis adjusting for the family-centered care in ICU scoreas well as several patient-level and family member-level covariates ... 8
10 Coefficient estimates from the sensitivity analysis using multiple imputation of missing outcomes ... 8
11 Coefficient estimates from the sensitivity analysis using multiple imputation of missing outcomes, adjusted for several patient-level and family member-level covariates ... 9
12 Coefficient estimates from the *post-hoc* sensitivity analysis excluding family members returned the QoC survey questionnaire later than 14 days after T1. ... 11
13 Coefficient estimates from the *post-hoc* sensitivity analysis adjusting for time to return of the QoC survey questionnaire ... 11
14 Coefficient estimates for the subgroup analysis on the primary outcome FS-ICU-24-R with planned vs. unplanned ICU admission as subgroup variable ... 12
15 Coefficient estimates for the subgroup analysis on the secondary outcome QQPPI with the family-centered care (FCC) in ICU score as subgroup variable ... 14
16 Coefficient estimates from the additional analysis of the primary outcome FS-ICU-24R, investigating consistency of the intervention. ... 18
17 Coefficient estimates from the additional analysis of the secondary outcome QQPPI, investigating consistency of the intervention. ... 18
18 Coefficient estimates from the additional analysis of the secondary outcome FPSQ, investigating consistency of the intervention. ... 19
19 Minimal Clinically Important Difference (MCID) for all outcomes ... 20
20 Intraclass correlation coefficients (ICC) ... 20

List of eFigures

1 Family support intervention ... 3
2 Histogram of the time to return of the QoC survey questionnaires by study participants ... 10
3 Forest plot of treatment effect estimates on the primary outcome FS-ICU-24-R within subgroups (categorical subgroup variables) ... 13
4 Forest plot of treatment effect estimates on the primary outcome FS-ICU-24-R within subgroups (continuous subgroup variables) ... 13
5 Diagnostic plots for the residuals from model 1 applied to the primary outcome, FS-ICU-24R. 15
6 Diagnostic plots for the residuals from model 5 applied to the primary outcome, FS-ICU-24R. 16
7 Violin plots showing the distribution of the primary outcome, FS-ICU-24R, by treatment. ... 17

© 2025 Naef R. et al; the FICUS Study Group. JAMA Internal Medicine.

1 Intervention characteristics

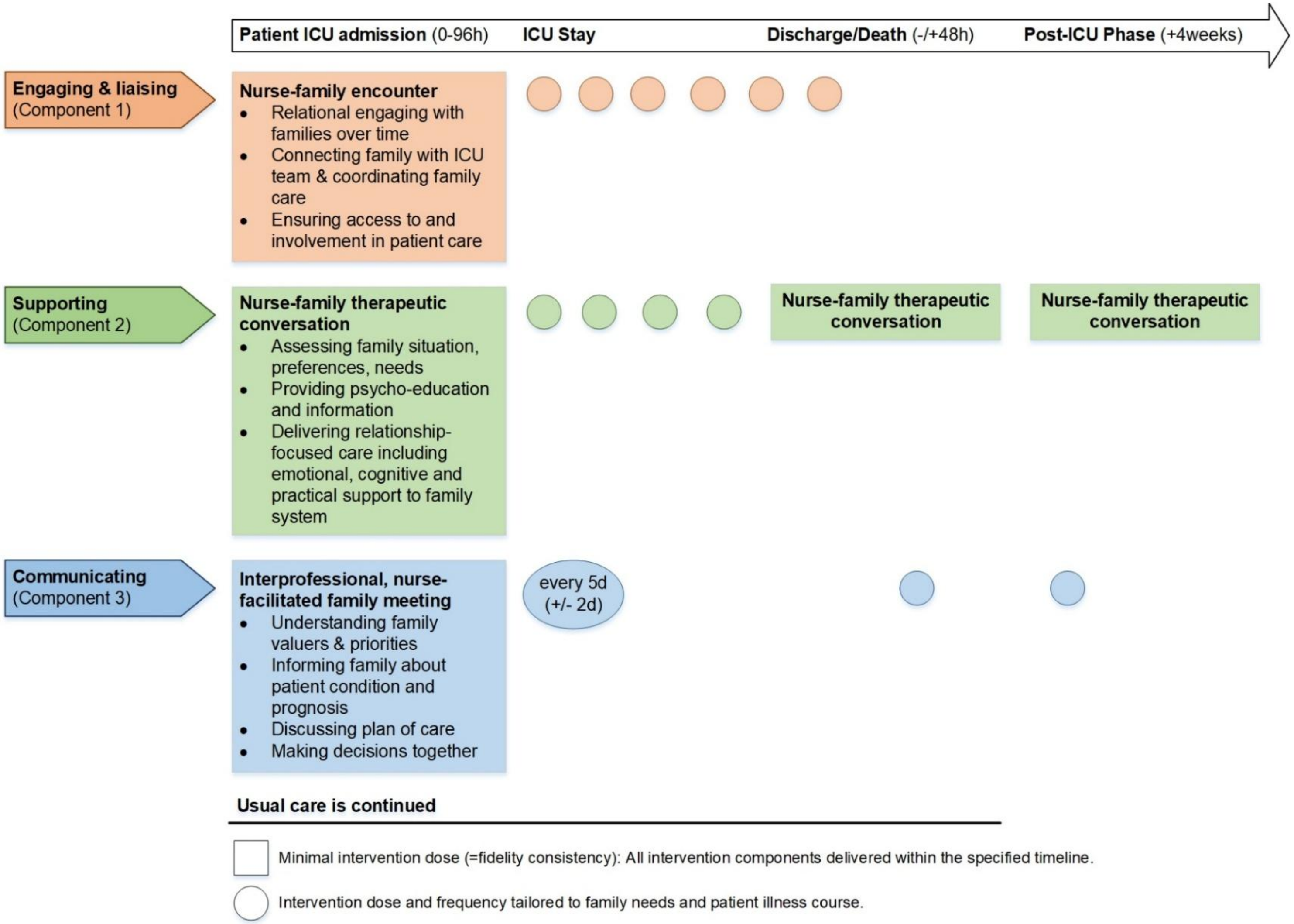

eFigure 1: Family Support Intervention

© 2025 Naef R. et al; the FICUS Study Group. JAMA Internal Medicine.

eTable 1: Characteristics of intervention contacts.

Intervention characteristics	Intervention contacts n=5’161	Missing (%)
Intervention delivery mode, n (%)		0.1
In-person at hospital	3’146 (61.0%)	
Over the phone	1’731 (33.6%)	
Online meeting tool	226 (4.4%)	
Hybrid format	51 (1.0%)	
Number of family members per intervention contact (incl. patient), median (q1, q3)	1 (1, 2)	0.7
Type of persons present during intervention		
Study participant, n (%)	4’783 (93.1%)	0.5
Study participant and patient, n (%)	1’101 (21.4%)	0.5
Study participant and additional family member, n (%)	1’456 (28.3%)	0.5
Intervention deliverers, n (%)		1.7
Family nurse only	3’947 (77.8%)	
Family nurse and ICU nurse(s)[a]	235 (4.6%)	
Family nurse and ICU physician(s)[a]	551 (10.9%)	
Family nurse and ICU nurse(s) and physician(s)[a]	278 (5.5%)	
Family nurse and other professionals (no ICU nurse or ICU physician)	61 (1.2%)	
Family nurse intervention activity (multiple answers can apply per intervention contact)		
Entering and maintaining caring relationships with family, n (%)	2’492 (48.3%)	0.0
Assessing family situation and needs, n (%)	1’365 (26.5%)	0.0
Offering relationship-focused interventions, n (%)	1’758 (34.1%)	0.0
Offering psycho-educational interventions, n (%)	2’389 (46.3%)	0.0
Liaising family with others and coordinating family care, n (%)	1’386 (26.9%)	0.0
Communicating with family as interprofessional team, n (%)	1’370 (26.6%)	0.0
Engaging in shared decision-making with family, n (%)	1’046 (20.3%)	0.0
Facilitating transitions of family, n (%)	1’255 (24.3%)	0.0
Concluding / terminating caring relationship with family, n (%)	392 (7.6%)	0.0
Other, n (%)	85 (1.7%)	0.0
Referrals of family to other services, yes, n (%)	423 (8.2%)	0.2

[a] Other professionals besides ICU nurse(s) and ICU physician(s) may have been present.

© 2025 Naef R. et al; the FICUS Study Group. JAMA Internal Medicine.

eTable 2: Characteristics of intervention contacts with referrals.

**Intervention characteristics**	**Intervention contacts with referrals n=423**	**Missing (%)**
Reason for initiating these referrals, n (%)		0.0
Initiated because of study intervention (would not have been initiated otherwise)	217 (51.3%)	
Possibly initiated because of study intervention (might have been initiated otherwise)	135 (31.9%)	
Initiated independent of study intervention (would have been initiated otherwise)	71 (16.8%)	
Type of referrals made (multiple answers can apply per intervention contact)		
Social work, n (%)	151 (35.7%)	0.0
Chaplaincy / Spiritual care, n (%)	0 (0%)	0.0
Ethics consultation, n (%)	21 (5.0%)	0.0
Psychological counselling, n (%)	69 (16.3%)	0.0
Emergency care team, n (%)	17 (4.0%)	0.0
Family physician, n (%)	140 (33.1%)	0.0
Other service within hospital, n (%)	27 (6.4%)	0.0
Other service outside hospital, n (%)	33 (7.8%)	0.0

© 2025 Naef R. et al; the FICUS Study Group. JAMA Internal Medicine.

2 Sensitivity analyses of the primary outcome family satisfaction with ICU care (FS-ICU-24R)

This section presents all planned sensitivity analyses of the primary outcome family satisfaction with ICU care (FS-ICU-24R). The models are numbered as in the statistical analysis plan[1]. The coefficient estimates from the sensitivity analyses which include one additional cluster-level covariate each are shown in eTables 3–5 (models 2–4). The estimated intervention effects are of similar size as the intervention effect estimated in the main analysis (main text, Table 4) and the corresponding p-values ($0.01 < p < 0.05$) also provide moderate evidence against the null hypothesis of no intervention effect[2]. As in the main analysis, the between-cluster variance in these models is estimated as 0 which results in a singular fit of the model and an ICC of 0.

The coefficient estimates from the sensitivity analyses which include a set of patient- and participant-level covariates, alone or in combination with one cluster-level covariate, are shown in eTables 6–9 (models 5–8). The estimated intervention effects from these models are slightly larger than those from models 1–4 but with wider 95 % confidence intervals. Thus, they only provide weak evidence ($0.05 < p < 0.1$) against the null hypothesis of no intervention effect[2]. This may be due to the reduced number of participants in the analyses, which results from missing values in certain covariates.

The coefficient estimates from the sensitivity analyses done with multiply imputed data are shown in eTables 10–11 (models 9–10). The results shown in eTable 10 confirm those shown for the main analysis (main text, Table 4), which was performed by the same statistical model but applied to complete cases only (excluding participants with missing values for the primary outcome). The results shown in eTable 11 show a slightly smaller intervention effect estimate but stronger evidence against the null hypothesis compared to those in eTable 6 resulting from the same statistical model, which may be due to the gain in power due to multiple imputation of missing values (of the primary outcome and of covariates). The only covariate which is significantly associated with the primary outcome is the SAPS2-score, where higher SAPS2-scores (higher estimated mortality) are associated with higher values of the FS-ICU-24R (higher family satisfaction with ICU care).

eTable 3: Coefficient estimates from the sensitivity analysis (model 2) on the primary outcome *Family satisfaction with ICU Care (FS-ICU-24R)*, estimated by a linear mixed-effects model with a random intercept per cluster (ICU) and the certification of the cluster covariate in addition to the randomized treatment. A total of 794 participants from 16 centres were included in the model.

Model term	Coefficient	95%-confidence interval	p-value
(Intercept)	78.95	from 77.41 to 80.48	
B vs. A/Au certification	1.74	from -0.61 to 4.08	0.15
Intervention vs. Control	2.34	from 0.26 to 4.42	0.028

eTable 4: Coefficient estimates from the sensitivity analysis (model 3) on the primary outcome *Family satisfaction with ICU Care (FS-ICU-24R)*, estimated by a linear mixed-effects model with a random intercept per cluster (ICU) and nurse staffing of the cluster as covariate in addition to the randomized treatment. A total of 794 participants from 16 centres were included in the model.

Model term	Coefficient	95%-confidence interval	p-value
(Intercept)	81.37	from 76.42 to 86.33	
Cluster nurse staffing (FTE/operated beds, %)	-0.44	from -1.48 to 0.61	0.41
Intervention vs. Control	2.47	from 0.38 to 4.56	0.021

© 2025 Naef R. et al; the FICUS Study Group. JAMA Internal Medicine.

eTable 5: Coefficient estimates from the sensitivity analysis (model 4) on the primary outcome *Family satisfaction with ICU Care (FS-ICU-24R)*, estimated by a linear mixed-effects model with a random intercept per cluster (ICU) and the family-centered care in ICU score of the cluster as covariate in addition to the randomized treatment. A total of 794 participants from 16 centres were included in the model.

Model term	Coefficient	95%-confidence interval	p-value
(Intercept)	80.98	from 71.29 to 90.68	
Family-centered care in ICU score	-0.55	from -3.82 to 2.73	0.74
Intervention vs. Control	2.40	from 0.32 to 4.48	0.024

eTable 6: Coefficient estimates from the sensitivity analysis (model 5) on the primary outcome *Family satisfaction with ICU Care (FS-ICU-24R)*, estimated by a linear mixed-effects model with a random intercept per cluster (ICU) and several patient-level and family member-level covariates in addition to the randomized treatment. A total of 767 participants from 16 centres were included in the model.

Model term	Coefficient	95%-confidence interval	p-value
(Intercept)	73.65	from 67.90 to 79.40	
Patient age (years)	0.00	from -0.07 to 0.07	0.98
Planned vs. unplanned ICU admission	0.97	from -2.11 to 4.04	0.54
SAPS2 score	0.08	from 0.02 to 0.15	0.0089
FM parent vs. spouse/partner	2.97	from -0.29 to 6.23	0.074
FM child vs. spouse/partner	1.72	from -0.92 to 4.36	0.20
FM other relationship vs. spouse/partner	1.12	from -2.49 to 4.74	0.54
Prior ICU experience as patient vs. no experience	0.53	from -4.12 to 5.17	0.82
Prior ICU experience as family member vs. no experience	-1.08	from -3.33 to 1.17	0.35
Prior ICU experience as both vs. no experience	1.45	from -3.43 to 6.34	0.56
Intervention vs. Control	2.74	from -0.13 to 5.61	0.06

eTable 7: Coefficient estimates from the sensitivity analysis (model 6) on the primary outcome *Family satisfaction with ICU Care (FS-ICU-24R)*, estimated by a linear mixed-effects model with a random intercept per cluster (ICU) and the certification of the cluster as well as several patient-level and family member-level covariates in addition to the randomized treatment. A total of 767 participants from 16 centres were included in the model.

Model term	Coefficient	95%-confidence interval	p-value
(Intercept)	73.59	from 67.86 to 79.32	
B vs. A/Au certification	2.34	from -0.82 to 5.50	0.13
Patient age (years)	-0.01	from -0.08 to 0.06	0.79
Planned vs. unplanned ICU admission	0.74	from -2.33 to 3.82	0.64
SAPS2 score	0.09	from 0.03 to 0.16	0.0043
FM parent vs. spouse/partner	2.80	from -0.47 to 6.06	0.093
FM child vs. spouse/partner	1.70	from -0.94 to 4.33	0.21
FM other relationship vs. spouse/partner	1.00	from -2.61 to 4.61	0.59
Prior ICU experience as patient vs. no experience	0.55	from -4.09 to 5.19	0.82
Prior ICU experience as family member vs. no experience	-1.25	from -3.51 to 1.01	0.28
Prior ICU experience as both vs. no experience	1.39	from -3.49 to 6.27	0.58
Intervention vs. Control	2.60	from -0.21 to 5.42	0.067

© 2025 Naef R. et al; the FICUS Study Group. JAMA Internal Medicine.

eTable 8: Coefficient estimates from the sensitivity analysis (model 7) on the primary outcome *Family satisfaction with ICU Care (FS-ICU-24R)*, estimated by a linear mixed-effects model with a random intercept per cluster (ICU) and the nurse staffing of the cluster as well as several patient-level and family member-level covariates in addition to the randomized treatment. A total of 767 participants from 16 centres were included in the model.

Model term	Coefficient	95%-confidence interval	p-value
(Intercept)	75.90	from 67.01 to 84.80	
Cluster nurse staffing (FTE/operated beds, %)	-0.47	from -1.99 to 1.04	0.50
Patient age (years)	0.00	from -0.08 to 0.07	0.97
Planned vs. unplanned ICU admission	0.90	from -2.19 to 3.99	0.57
SAPS2 score	0.09	from 0.02 to 0.15	0.0072
FM parent vs. spouse/partner	2.96	from -0.30 to 6.22	0.075
FM child vs. spouse/partner	1.72	from -0.92 to 4.37	0.20
FM other relationship vs. spouse/partner	1.02	from -2.61 to 4.64	0.58
Prior ICU experience as patient vs. no experience	0.51	from -4.14 to 5.16	0.83
Prior ICU experience as family member vs. no experience	-1.11	from -3.36 to 1.15	0.34
Prior ICU experience as both vs. no experience	1.45	from -3.44 to 6.33	0.56
Intervention vs. Control	2.79	from -0.18 to 5.75	0.063

eTable 9: Coefficient estimates from the sensitivity analysis (model 8) on the primary outcome *Family satisfaction with ICU Care (FS-ICU-24R)*, estimated by a linear mixed-effects model with a random intercept per cluster (ICU) and the family-centered care in ICU score of the cluster as well as several patient-level and family member-level covariates in addition to the randomized treatment. A total of 767 participants from 16 centres were included in the model.

Model term	Coefficient	95%-confidence interval	p-value
(Intercept)	71.42	from 57.01 to 85.83	
Family-centered care in ICU score	0.77	from -3.85 to 5.39	0.72
Patient age (years)	0.00	from -0.08 to 0.07	0.96
Planned vs. unplanned ICU admission	0.99	from -2.10 to 4.09	0.53
SAPS2 score	0.09	from 0.02 to 0.15	0.0072
FM parent vs. spouse/partner	2.95	from -0.31 to 6.22	0.076
FM child vs. spouse/partner	1.71	from -0.93 to 4.35	0.20
FM other relationship vs. spouse/partner	1.10	from -2.52 to 4.72	0.55
Prior ICU experience as patient vs. no experience	0.54	from -4.11 to 5.19	0.82
Prior ICU experience as family member vs. no experience	-1.12	from -3.38 to 1.14	0.33
Prior ICU experience as both vs. no experience	1.49	from -3.39 to 6.38	0.55
Intervention vs. Control	2.73	from -0.27 to 5.74	0.071

eTable 10: Coefficient estimates from the sensitivity analysis (model 9) on the primary outcome *Family satisfaction with ICU Care (FS-ICU-24R)*, estimated by a linear mixed-effects model with a random intercept per cluster (ICU), as model 1 (main text, Table 4), but applied to a multiply imputed dataset, including all 885 study participants. The column fmi shows the fraction of missing information.

Model term	Coefficient	95%-confidence interval	p-value	fmi
(Intercept)	79.33	from 77.84 to 80.82	< 0.0001	0.16
Intervention vs. Control	2.44	from 0.31 to 4.57	0.025	0.12

eTable 11: Coefficient estimates from the sensitivity analysis (model 10) on the primary outcome *Family satisfaction with ICU Care (FS-ICU-24R)*, estimated by a linear mixed-effects model with a random intercept per cluster (ICU) and several patient-level and family member-level covariates in addition to the randomized treatment, as model 5 (eTable 6), but applied to a multiply imputed dataset, including all 885 study participants. The column fmi shows the fraction of missing information.

Model term	Coefficient	95%-confidence interval	p-value	fmi
(Intercept)	74.28	from 68.47 to 80.10	< 0.0001	0.14
Patient age (years)	0.00	from -0.08 to 0.08	0.99	0.17
Planned vs. unplanned ICU admission	0.64	from -2.44 to 3.73	0.68	0.18
SAPS2 score	0.08	from 0.01 to 0.14	0.017	0.17
FM parent vs. spouse/partner	2.28	from -1.01 to 5.57	0.17	0.18
FM child vs. spouse/partner	1.45	from -1.25 to 4.15	0.29	0.15
FM other relationship vs. spouse/partner	1.55	from -2.10 to 5.19	0.41	0.11
Prior ICU experience as patient vs. no experience	-0.26	from -5.00 to 4.48	0.91	0.23
Prior ICU experience as family member vs. no experience	-1.17	from -3.48 to 1.13	0.32	0.14
Prior ICU experience as both vs. no experience	0.40	from -4.44 to 5.24	0.87	0.10
Intervention vs. Control	2.66	from 0.31 to 5.00	0.026	0.11

3 Distribution of time to return of quality of care (QoC) questionnaires

eFigure 2 shows the distribution of the time to return of the QoC survey questionnaires after ICU discharge or death of the patient (T1), which is relevant for the primary outcome.

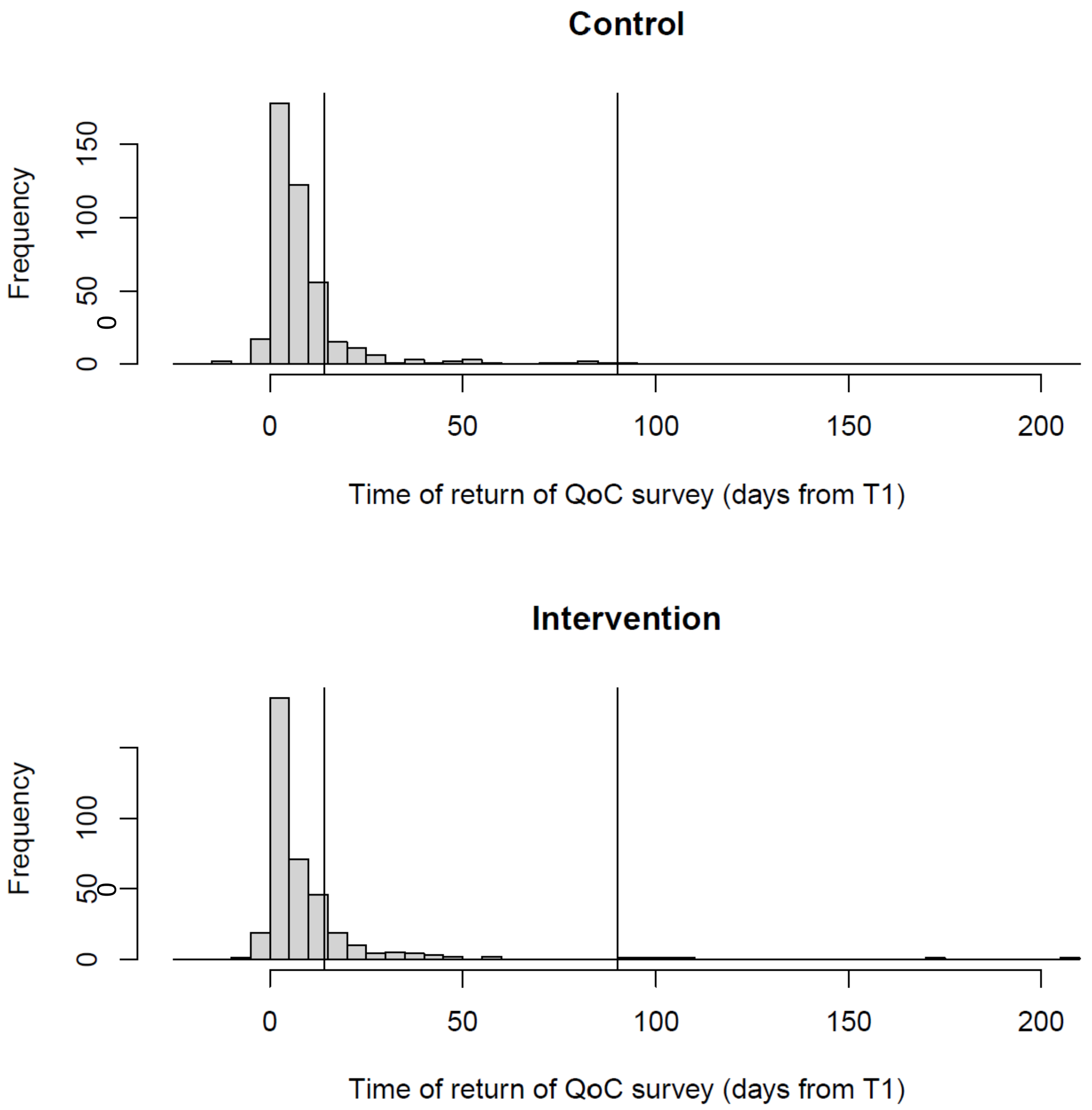

eFigure 2: Histogram of the time to return of the QoC survey questionnaires by study participants (number of days from the ICU discharge or death of the patient, i.e. T1) in both trial arms. Vertical lines show the end of the time window of 90 days was defined in the statistical analysis plan[1] whereas 14 days were originally planned in the published study protocol[3].

© 2025 Naef R. et al; the FICUS Study Group. JAMA Internal Medicine.

4 *Post-hoc* sensitivity analyses of the primary outcome with regard to time to return of (QoC) questionnaires

Due to the large time window for the primary outcome (return of QoC questionnaires within 90 days post ICU discharge or death of the patient), we performed two sensitivity analyses of the primary outcome. eTable 12 shows a sensitivity analysis of the primary outcome, performed as the main analysis (main text, Table 4) but excluding family members who returned the QoC survey questionnaire later than 14 days after T1. eTable 13 shows another sensitivity analysis of the primary outcome, performed as the main analysis but with the time between T1 and return of the QoC survey questionnaire as additional covariate. The result of both sensitivity analyses is quite similar to the main analysis (main text, Table 4), and even tends to be slightly more clear.

eTable 12: Effect size estimate for the effect of the Intervention vs. Control on the primary outcome *Family satisfaction with ICU Care (FS-ICU-24R)*, estimated by a linear mixed-effects model with a random intercept per cluster (ICU), when excluding family members who returned the QoC survey questionnaire later than 14 days after T1 (compare eFigure 1). A total of 682 participants from 16 centres were included in the model.

Model term	Coefficient	95%-confidence interval	p-value
(Intercept)	79.6	from 78.11 to 81.09	
Intervention vs. Control	2.7	from 0.50 to 4.89	0.016

eTable 13: Effect size estimate for the effect of the Intervention vs. Control on the primary outcome *Family satisfaction with ICU Care (FS-ICU-24R)*, estimated by a linear mixed-effects model with a random intercept per cluster (ICU), adjusting for the time between T1 and return of the QoC survey questionnaire (compare eFigure 1). A total of 785 participants from 16 centres were included in the model.

Model term	Coefficient	95%-confidence interval	p-value
(Intercept)	80.17	from 78.52 to 81.81	
Days between T1 and return of QoC survey	-0.09	from -0.19 to 0.01	0.068
Intervention vs. Control	2.53	from 0.46 to 4.60	0.017

5 Subgroup analyses of the primary outcome

The model coefficients of the subgroup analysis investigating whether unplanned vs. planned ICU ad- mission of the patient modifies the intervention effect on the primary outcome FS-ICU-24R are shown in eTable 14. Treatment effects within subgroups and the corresponding differences in treatment effects between subgroups are shown in eFigures 2 and 3.

It is important to note that the “Intercept” in eTable 14 represents the mean FS-ICU-24R for participants in the Control arm who were family members of a patient with planned ICU admission (reference category for study arm and ICU admission). The coefficient for "Unplanned vs. planned ICU admission" indicates that the FS-ICU-24R in the control arm was higher when the patient had an unplanned ICU admission. The coefficient for “Intervention vs. Control” indicates that when the patient had a planned ICU admission the FS-ICU-24R was higher in the intervention arm than in the control arm. This coefficient (together with its 95 % confidence interval) is also shown in eFigure 2 (left panel, red complement for Unplanned.ICU.admission.Yes). The coefficient for “Unplanned vs. planned ICU admission x Intervention vs. Control” indicates that this intervention effect was reduced (almost dissipates) when the patient had an unplanned ICU admission, and this interaction effect is the main result from this analysis. This coefficient (together with its 95 % confidence interval) is also shown in eFigure 2 (right panel, Unplanned.ICU.admission.Yes) where it can also be seen that this was the only significant treatment effect difference (interaction), laying completely below 0. It should be noted that most patients had an unplanned ICU admission (control 89.9 %, intervention 77.1 %, main text Table 2), with an intervention effect slightly smaller than overall, whereas the much larger intervention effect was observed in the relatively small subgroup of patients with planned ICU admission.

eTable 14: Coefficient estimates for the subgroup analysis on the primary outcome *FS-ICU-24-R* with planned vs. unplanned ICU admission as subgroup variable. A total of A total of 779 participants from 16 centres were included in the model.

Model term	Coefficient	95%-confidence interval	p-value
(Intercept)	74.39	from 69.93 to 78.85	
Unplanned vs. planned ICU admission	5.40	from 0.70 to 10.11	0.024
Intervention vs. Control	8.67	from 3.13 to 14.20	0.0022
Unplanned vs. planned ICU admission x Intervention vs. Control	-6.82	from -12.80 to -0.83	0.026

© 2025 Naef R. et al; the FICUS Study Group. JAMA Internal Medicine.

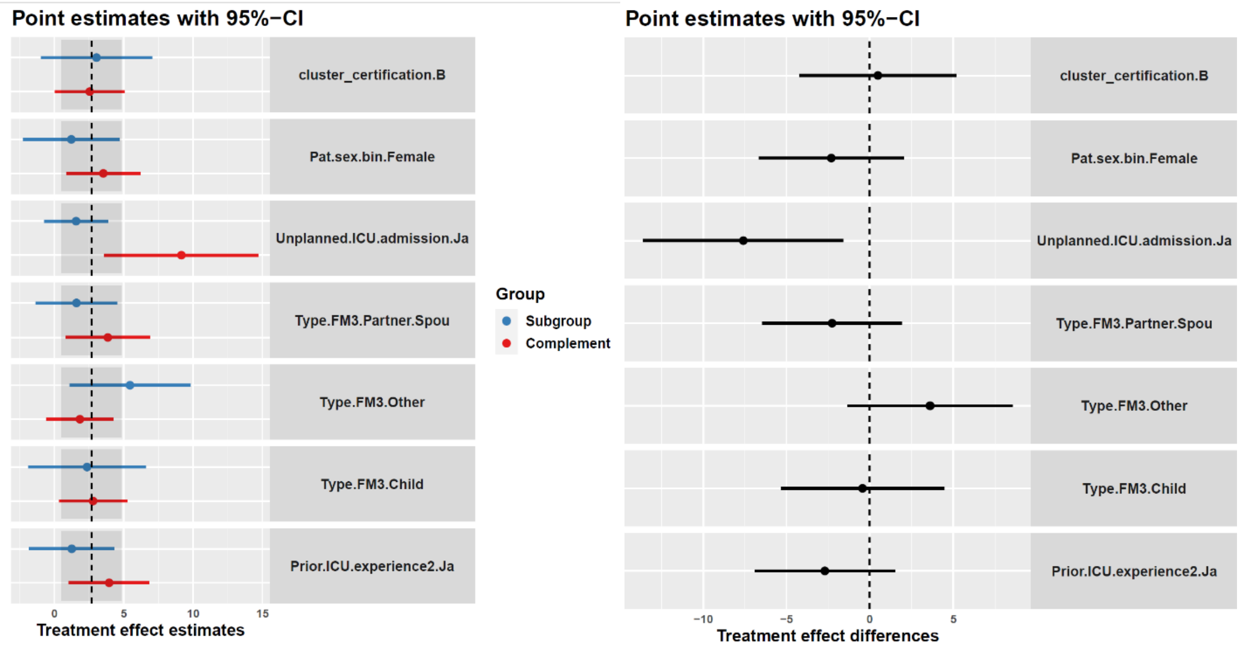

eFigure 3: Forest plot of treatment effect estimates on the primary outcome *FS-ICU-24-R* within subgroups (blue) and the corresponding complementary group for categorical subgroup variables (left panel) and corresponding differences in treatment effects between subgroups, i.e., the interaction between treatment and subgroup variable (right panel). In the left panel the dotted vertical line represents the treatment effect estimate overall, in the right panel it represents the null hypothesis of no difference in treatment effects. Please note that no treatment effect could be estimated for the subgroup of patients with sex reported as non-binary/trans, as there was only one patient in this group.

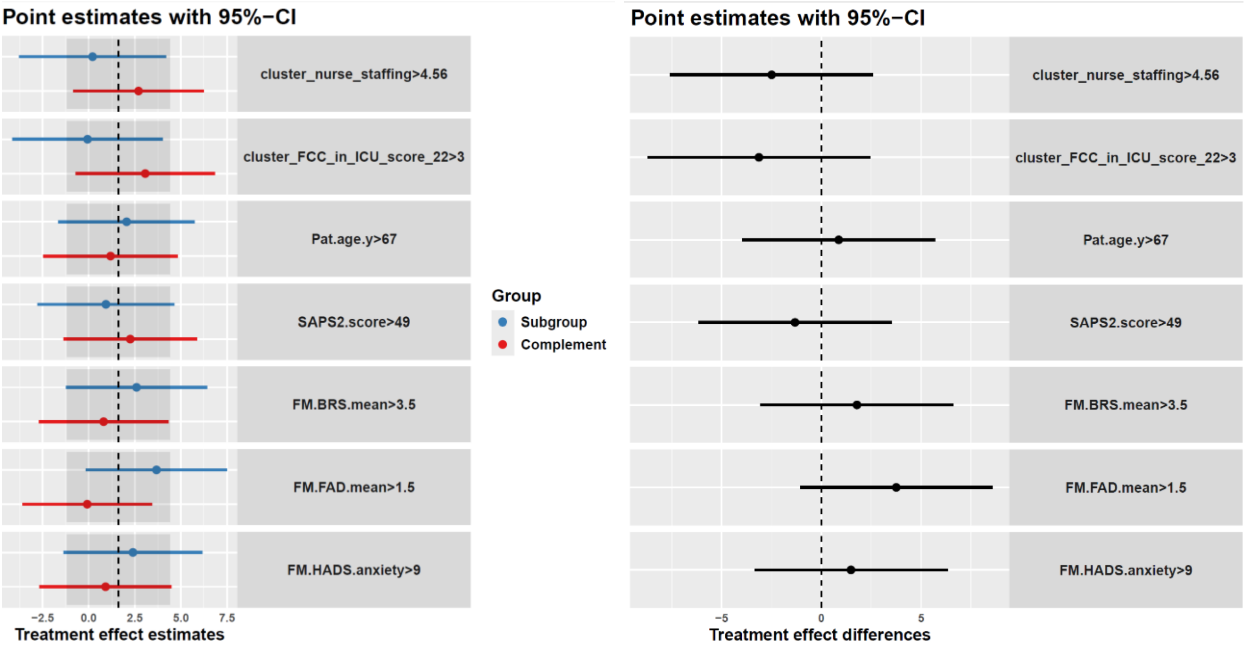

eFigure 4: Forest plot of treatment effect estimates on the primary outcome *FS-ICU-24-R* within subgroups (blue) and the corresponding complementary group for continuous subgroup variables that were dichotomized at the median value, i.e, < median vs. ≥ median (left panel) and corresponding differences in treatment effects between subgroups, i.e., the interaction between treatment and subgroup variable (right panel). In the left panel the dotted vertical line represents the treatment effect estimate overall, in the right panel it represents the null hypothesis of no difference in treatment effects.

© 2025 Naef R. et al; the FICUS Study Group. JAMA Internal Medicine.

6 Subgroup analyses of the secondary outcome quality of communication (QQPPI)

The model coefficients of the subgroup analysis investigating whether the family-centered care (FCC) in ICU score modifies the intervention effect on the secondary outcome quality of communication (QQPPI) are shown in eTable 15.

It is important to note that the FCC in ICU score was centered (subtracting the mean from each value) to improve the interpretability of coefficients in eTable 15 and that lower values of FCC mean stronger family-centeredness (and vice versa). The "Intercept" represents the mean QQPPI for participants in clusters with mean FCC in ICU score (of 2.93) in the Control arm (reference category for study arm). The coefficient for "Family-centered care (FCC) in ICU score" indicates the decrease in QQPPI per increase (worsening) in the FCC in ICU score by one unit in the control arm (slope). The coefficient for "Intervention vs. Control" indicates that for patients from clusters with mean FCC in ICU score the QQPPI was higher in the intervention arm than in the control arm. The coefficient for "FCC in ICU score x Intervention vs. Control" indicates the change in the slope for the FCC in ICU score on QQPPI in patients with the intervention (an even steeper decrease) compared to control, which can be interpreted as a stronger intervention effect when the family-centeredness at baseline was stronger. This interaction effect is the main result from this analysis.

eTable 15: Coefficient estimates for the subgroup analysis on the secondary outcome *QQPPI* with the family-centered care (FCC) in ICU score as subgroup variable. A total of A total of 778 participants from 16 centres were included in the model.

Model term	Coefficient	95%-confidence interval	p-value
(Intercept)	3.45	from 3.33 to 3.56	
Family-centered care (FCC) in ICU score	-0.14	from -0.40 to 0.13	0.29
Intervention vs. Control	0.40	from 0.23 to 0.56	0.0002
FCC in ICU score x Intervention vs. Control	-1.22	from -2.17 to -0.26	0.016

7 Distribution of the primary outcome and diagnostic plots of the residuals

The normality assumption was violated for the main analysis model of the primary outcome as well as the sensitivity analysis (model 5) which are reported in Table 4 of the main text (eFigures 5 and 6). The quantile–quantile plot (Q-Q-plot) of the participant-level residuals in the top right panel of each figure shows this violation of normality as the residuals show a curvature rather than being nicely scattered along the diagonal line. However, log-transformation would even worsen this violation of normality, which is likely due to a ceiling effect in the primary outcome (many values close to the maximum of 100 and few values <50), as shown in eFigure 7. Moreover, this ceiling effect more strongly affects the intervention than the control arm and should thus reduce rather than increase the estimated intervention effect (and the statistical power) when normality assumed. Further, the Q-Q-plot of the cluster-level residuals in the bottom right panel shows that these residuals (and thus the between cluster variance) are zero for the main analysis (eFigure 5), corresponding with the ICC of zero (see Section 10), whereas the cluster-level residuals from the sensitivity analysis are normally distributed (eFigure 6).

© 2025 Naef R. et al; the FICUS Study Group. JAMA Internal Medicine.

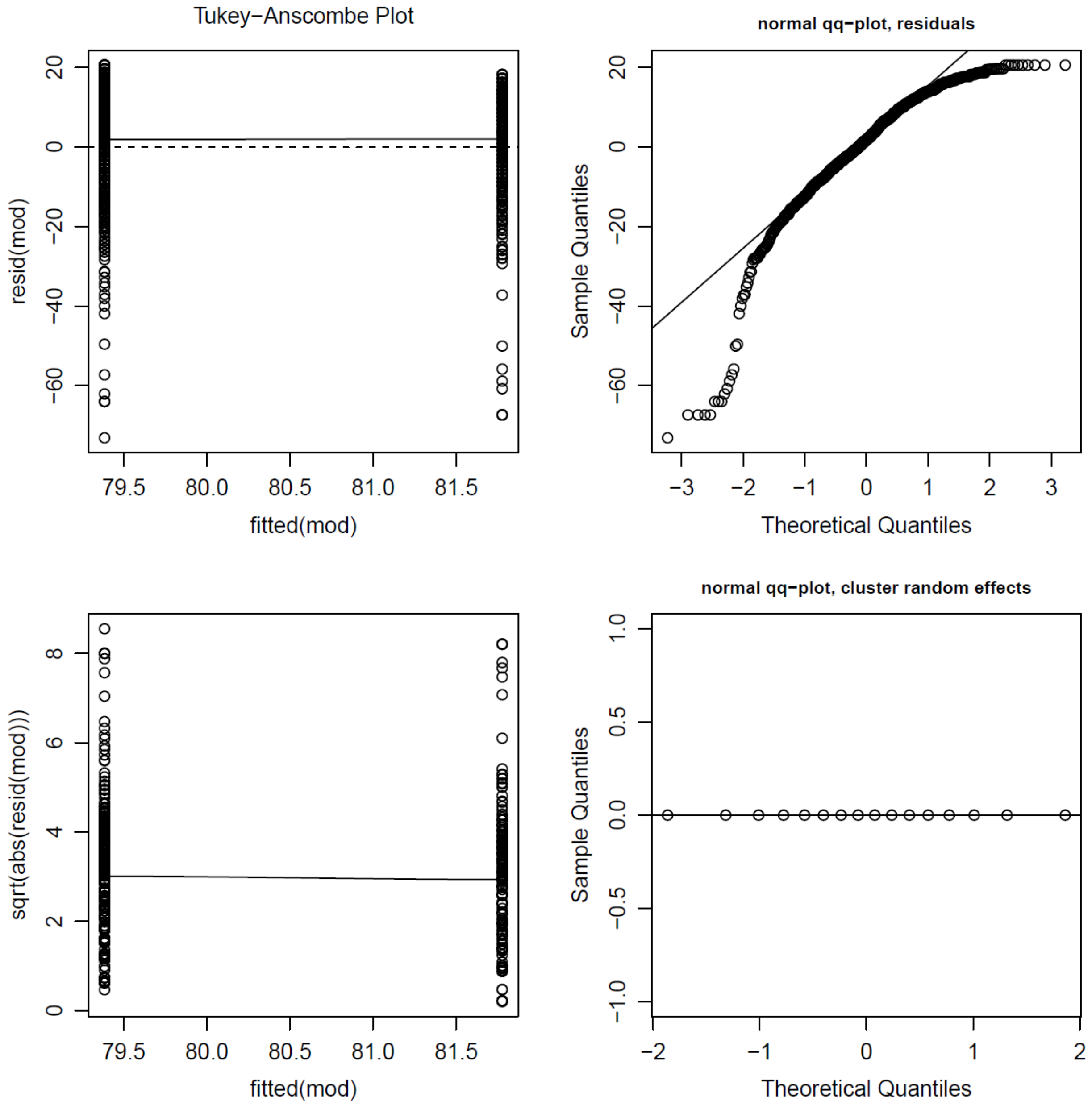

eFigure 5: Diagnostic plots for the residuals from model 1 applied to the primary outcome, FS-ICU-24R (main text, Table 4).

© 2025 Naef R. et al; the FICUS Study Group. JAMA Internal Medicine.

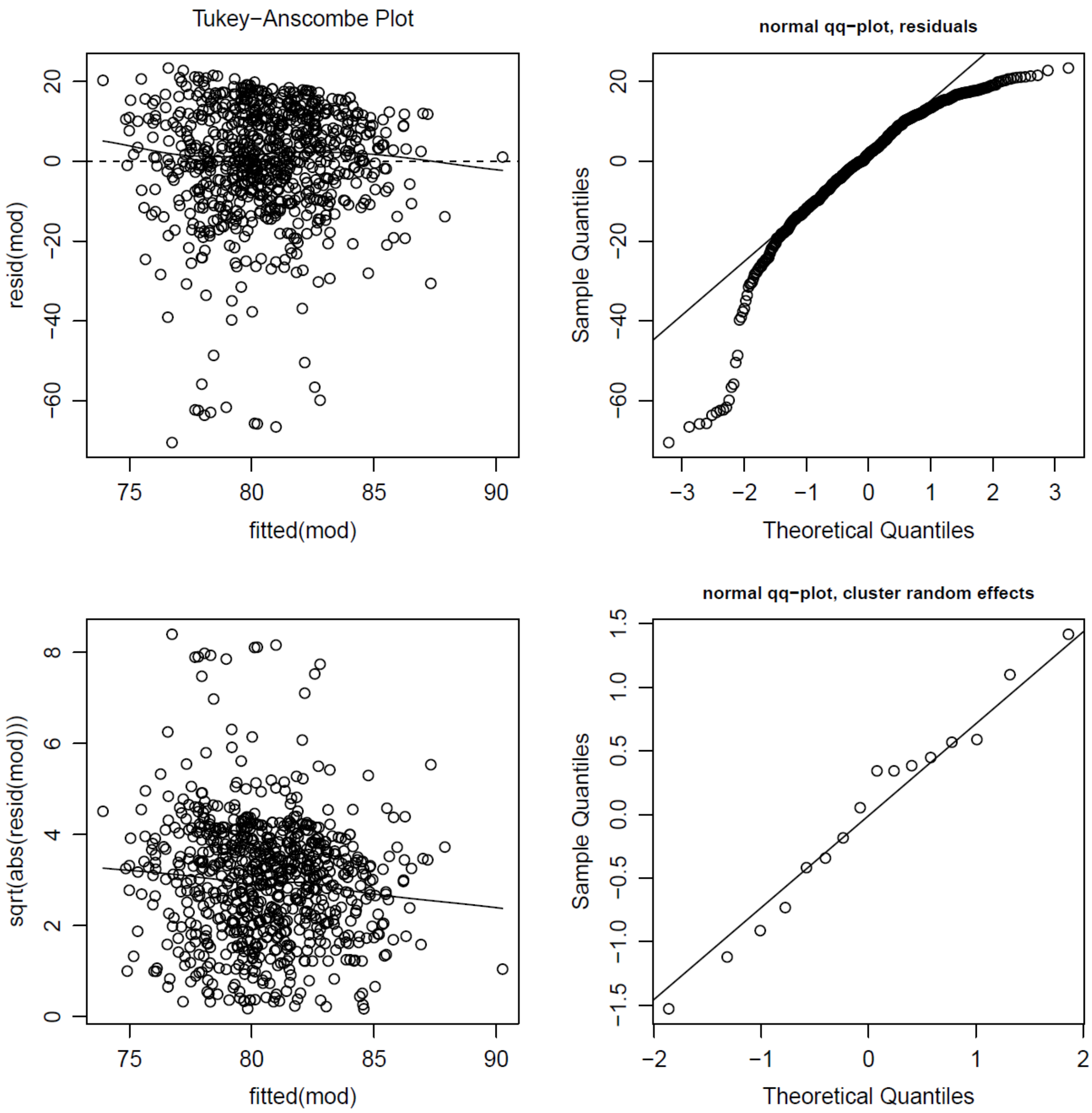

eFigure 6: Diagnostic plots for the residuals from model 5 applied to the primary outcome, FS-ICU-24R (eTable 6).

© 2025 Naef R. et al; the FICUS Study Group. JAMA Internal Medicine.

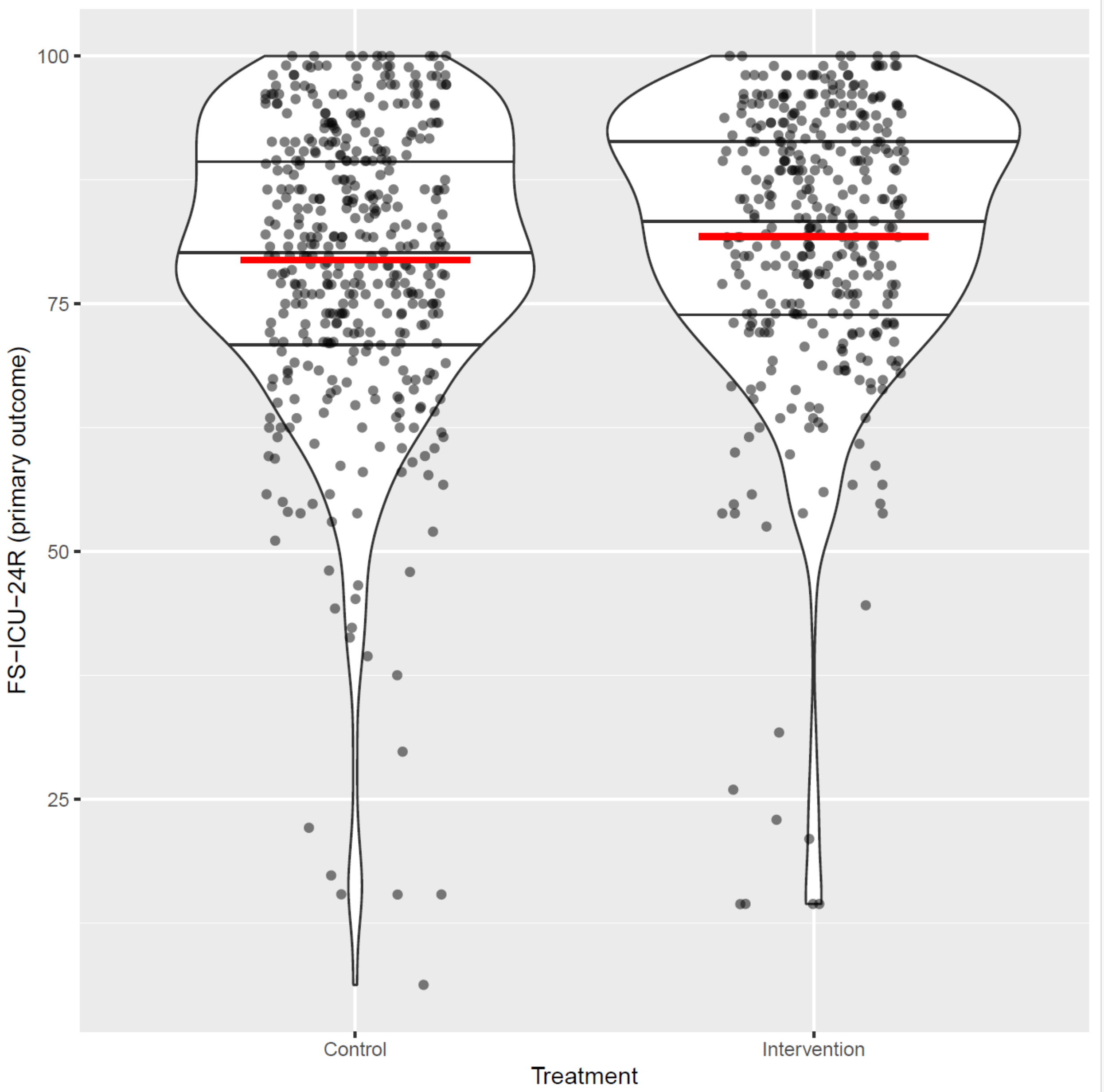

eFigure 7: Violin plots showing the distribution of the primary outcome, FS-ICU-24R, by treatment. The violin plots include the first quartile, the median and the third quartile as black horizontal lines. In addition, the mean is shown as read horizontal line. Individual data points are also shown in a jittered and semi-transparent way in order to show all of them.

© 2025 Naef R. et al; the FICUS Study Group. JAMA Internal Medicine.

8 Analyses investigating intervention fidelity (consistency)

The coefficient estimates from the additional analyses to investigate the effect of intervention fidelity, operationalized as the consistency of the intervention, are shown for the primary outcome FS-ICU-24R and the secondary outcomes QQPPI and nurse support FPSQ in eTables 16–18. eTable 16 shows that consistent intervention delivery had a more pronounced effect on the primary outcome than inconsistent delivery (both compared to usual care). The effect of consistent intervention delivery is statistically significant whereas the effect of inconsistent intervention delivery is not. eTables 17 and 18 show that both consistent and inconsistent intervention delivery had similar (and statistically significant) effects on these secondary outcomes (both compared to usual care), but in both cases the effect of inconsistent delivery was slightly more pronounced, which is unexpected.

eTable 16: Coefficient estimates from the additional analysis of the primary outcome *Family satisfaction with ICU Care (FS-ICU-24R)*, estimated by a linear mixed-effects model with a random intercept per cluster (ICU) and several patient-level and family member-level covariates, as model 5 (eTable 6), as well as the consistency of intervention delivery (instead of the randomized treatment) as explanatory variables. The model was applied to a multiply imputed dataset, including all 885 study participants. The column fmi shows the fraction of missing information.

Model term	Coefficient	95%-confidence interval	p-value	fmi
(Intercept)	74.30	from 68.49 to 80.11	< 0.0001	0.14
Patient age (years)	0.00	from -0.08 to 0.08	0.99	0.17
Planned vs. unplanned ICU admission	0.69	from -2.40 to 3.78	0.66	0.18
SAPS2 score	0.08	from 0.01 to 0.14	0.02	0.17
FM parent vs. spouse/partner	2.37	from -0.93 to 5.67	0.16	0.18
FM child vs. spouse/partner	1.48	from -1.22 to 4.18	0.28	0.15
FM other relationship vs. spouse/partner	1.51	from -2.14 to 5.16	0.42	0.12
Prior ICU experience as patient vs. no experience	-0.29	from -5.03 to 4.46	0.91	0.23
Prior ICU experience as family member vs. no experience	-1.13	from -3.44 to 1.18	0.34	0.14
Prior ICU experience as both vs. no experience	0.31	from -4.53 to 5.16	0.90	0.10
Consistent intervention delivery vs. usual care	3.87	from 0.29 to 7.45	0.034	0.04
Inconsistent intervention delivery vs. usual care	2.30	from -0.21 to 4.82	0.072	0.12

eTable 17: Coefficient estimates from the additional analysis of the secondary outcome *quality of communication (QQPPI)*, estimated by a linear mixed-effects model with a random intercept per cluster (ICU) and several patient-level and family member-level covariates, as well as the consistency of intervention delivery (instead of the randomized treatment) as explanatory variables (as in eTable 16 for the primary outcome). The model was applied to a multiply imputed dataset, including all 885 study participants. The column fmi shows the fraction of missing information.

Model term	Coefficient	95%-confidence interval	p-value	fmi
(Intercept)	3.49	from 3.15 to 3.82	< 0.0001	0.07
Patient age (years)	-0.01	from -0.01 to -0.00	0.0074	0.12
Planned vs. unplanned ICU admission	0.02	from -0.16 to 0.20	0.82	0.13
SAPS2 score	0.01	from 0.00 to 0.01	0.0013	0.15
FM parent vs. spouse/partner	0.03	from -0.15 to 0.21	0.75	0.16
FM child vs. spouse/partner	0.01	from -0.14 to 0.17	0.85	0.12
FM other relationship vs. spouse/partner	0.03	from -0.18 to 0.24	0.77	0.15
Prior ICU experience as patient vs. no experience	0.08	from -0.17 to 0.34	0.52	0.15
Prior ICU experience as family member vs. no experience	-0.08	from -0.21 to 0.05	0.21	0.08
Prior ICU experience as both vs. no experience	-0.03	from -0.29 to 0.24	0.85	0.07
Consistent intervention delivery vs. usual care	0.36	from 0.11 to 0.61	0.0051	0.03
Inconsistent intervention delivery vs. usual care	0.42	from 0.21 to 0.62	< 0.0001	0.04

© 2025 Naef R. et al; the FICUS Study Group. JAMA Internal Medicine.

eTable 18: Coefficient estimates from the additional analysis of the secondary outcome *nurse support (FPSQ)*, estimated by a linear mixed-effects model with a random intercept per cluster (ICU) and several patient-level and family member-level covariates, as well as the consistency of intervention delivery (instead of the randomized treatment) as explanatory variables (as in eTable 16 for the primary outcome). The model was applied to a multiply imputed dataset, including all 885 study participants. The column fmi shows the fraction of missing information.

Model term	Coefficient	95%-confidence interval	p-value	fmi
(Intercept)	42.68	from 36.80 to 48.55	< 0.0001	0.08
Patient age (years)	-0.12	from -0.19 to -0.05	0.0015	0.13
Planned vs. unplanned ICU admission	-0.41	from -3.50 to 2.69	0.80	0.12
SAPS2 score	0.10	from 0.04 to 0.17	0.0025	0.19
FM parent vs. spouse/partner	0.29	from -2.85 to 3.43	0.86	0.14
FM child vs. spouse/partner	-0.68	from -3.27 to 1.91	0.61	0.12
FM other relationship vs. spouse/partner	-0.95	from -4.57 to 2.66	0.60	0.14
Prior ICU experience as patient vs. no experience	1.90	from -2.42 to 6.23	0.39	0.12
Prior ICU experience as family member vs. no experience	-1.05	from -3.30 to 1.20	0.36	0.13
Prior ICU experience as both vs. no experience	-1.04	from -5.72 to 3.63	0.66	0.09
Consistent intervention delivery vs. usual care	8.93	from 4.39 to 13.48	0.00012	0.03
Inconsistent intervention delivery vs. usual care	9.11	from 5.43 to 12.80	< 0.0001	0.04

© 2025 Naef R. et al; the FICUS Study Group. JAMA Internal Medicine.

9 Minimal Clinically Important Difference (MCID) for all outcomes

eTable 19: Minimal Clinically Important Difference (MCID) for all outcomes calculated as $0.5 * SD$ (SD=standard deviation) and as $SEM = SD * \sqrt{1 - r}$ (SEM = Standard Error of Measurement) as described by scribed by[4]. The SD used is the residual standard deviation from the corresponding statistical models and r is Cronbach's alpha.

Outcome	sd	r	mcid_sd	mcid_sem
FSICU-24-R	14.90	0.93	7.45	3.94
FSICU-24-R Care	16.49	0.89	8.24	5.47
FSICU-24-R Decision-Making	15.27	0.88	7.63	5.29
QQPPI-14	0.85	0.95	0.42	0.19
ICE-FPSQ-14	14.62	0.95	7.31	3.27

10 Intraclass correlation coefficients

In the main analysis (main text, Table 4) the between-cluster variance was estimated as 0 which resulted in a singular fit of the model. As a consequence, the intraclass correlation coefficient (ICC) was zero. eTable 20 further shows the intraclass correlation coefficients (ICC) for the primary outcome FS-ICU-24R estimated from the sensitivity analysis (model 5), which is also shown in the main text (Table 4), and the ICC for the two secondary outcomes QQPPI and FPSQ.

eTable 20: Intraclass correlation coefficients (ICC) for the primary outcome FS-ICU-24R estimated from an alternative to the main model fitted with the R-package nlme (model1.nlme) and for the sensitivity analysis models 5 (both shown in the main text, Table 4) as well as for the secondary outcomes QQPPI and FPSQ.

	ICC_unadjusted	ICC_adjusted	ICC_conditional
model5	0.0099373940	0.0102114479	0.0099373940
model6	0.0087221255	0.0089952945	0.0087221255
model1.qqppi	0.0274432635	0.0286968398	0.0274432635
model1.fpsq	0.0383661862	0.0416180674	0.0383661862

11 References

[1] Felten Stefanie, Filipovic Miodrag, Jeitziner Marie-Madlen, Verweij Lotte, Riguzzi Marco, Naef Rahel. Multicomponent family support intervention in intensive care units: Statistical analysis plan for the cluster-randomized controlled FICUS trial *Trials.* 2024;25.

[2] Bland Martin. *An introduction to medical statistics*. Oxford University Pressfourth ed. 2015.

[3] Naef Rahel, Filipovic Miodrag, Jeitziner Marie-Madlen, et al. A multicomponent family support intervention in intensive care units: study protocol for a multicenter cluster-randomized trial (FICUS Trial) *Trials.* 2022;23:533.

[4] Mouelhi Yosra, Jouve Elisabeth, Castelli Christel, Gentile Stéphanie. How is the minimal clinically important difference established in health-related quality of life instruments? Review of anchors and methods *Health and quality of life outcomes.* 2020;18:1–17.

© 2025 Naef R. et al; the FICUS Study Group. JAMA Internal Medicine.

Acronyms

**A/Au**	Certification as A/Au hospital, i.e., major teaching hospital
**B**	Certification as B hospital, i.e., other teaching hospitals
**BRS**	Family resilience, as assessed by the Brief Resilience Scale (BRS-6)
**FAD**	Family functioning, assessed by the Family Assessment Device - General Functioning Scale (FAD- GF-12)
**FCC**	Family-centered care in ICU score
**FM**	Family member, abbreviation used in variable names
**fmi**	Fraction of missing information
**FPSQ**	Nurse support (secondary outcome), measured by the Family Perceived Support Questionnaire
**FS-ICU-24R**	Family satisfaction with ICU care (primary outcome)
**FTE**	Full-time equivalent
**HADS**	Hospital Anxiety and Depression Scale (HADS), with an anxiety and a depression score
**ICU**	Intensive care unit
**qq-plot**	Quantile–quantile plot
**q1**	First quartile of a distribution
**q3**	Third quartile of a distribution
**QoC**	Quality of care
**QQPPI**	Quality of communication (secondary outcome), measured by the Questionnaire on Quality of Physician–Patient Interaction
**SAPS2**	Simplified Acute Physiology Score (SAPS) II

R version and packages used to generate this report:
R version: R version 4.5.0 (2025-04-11)
Base packages: stats, graphics, grDevices, utils, datasets, methods, base
Other packages: effectsize 1.0.0, lmerTest 3.1-3, lme4 1.1-37, mice 3.17.0, dplyr 1.1.4, foreign 0.8-90, haven 2.5.4, ltm 1.2-0, polycor 0.8-1, msm 1.8.2, ggplot2 3.4.4, subtee 1.0.1, biostatUZH 2.2.7, MASS 7.3-65, survival 3.8-3, performance 0.13.0, broom.mixed 0.2.9.6, Matrix 1.7-3, xtable 1.8-4, stringr 1.5.1, tableone 0.13.2, knitr 1.50
This document was generated on April 24, 2025 at 10:51.

© 2025 Naef R. et al; the FICUS Study Group. JAMA Internal Medicine.
