# Supplementary material for: Nurse-Led Family Support Intervention for Families of Critically Ill Patients: The FICUS Cluster Randomized Clinical Trial
Source: JAMA Intern Med. 2025 Jul 28;185(9):1138–49. doi: 10.1001/jamainternmed.2025.3406 (PMC12558129; doi:10.1001/jamainternmed.2025.3406)
Supplement: Supplement 3. — Ficus Study Group [file jamainternmed-e253406-s003.pdf]

Supplemental Online Content: Nonauthor Collaborators
*First name, last name, and suffix (if applicable) are required and will appear in PubMed.

***Group Name(s): FICUS Study Group**

*First Name and Middle Initial(s)	*Last Name	*Suffix (eg, Jr, III)	Academic Degrees	Institution	Location (city, state/province, country)	Role or Contribution, eg, chair, principal investigator	Group (if more than 1 Group listed in the byline) and/or Subgroup (eg, Steering Committee)
Tessa	Allgaier			University Hospital Zurich	Zurich, ZH, Switzerland	Local research staff	
Nicole	Balsiger			Cantonal Hospital Baden	Baden, AG, Switzerland	Study coordinator	
Eun Joo	Beers			Cantonal Hospital Graubünden	Chur, GR, Switzerland	Study coordinator	
Sabine	Berger			Lindenhofspital	Bern, BE, Switzerland	Study coordinator	
Evelin	Bläs			University Hospital Zurich	Zurich, ZH, Switzerland	Local research staff	
Andreas	Bosshard			Lindenhofspital	Bern, BE, Switzerland	Local Co-Investigator	
Stephanie	Bossi			Cantonal Hospital Graubünden	Chur, GR, Switzerland	Study coordinator	
Tanja	Brülhart			N.A.		Family member expert	
Regine	Büdel			Kantonsspital St.Gallen	St. Gallen, SG, Switzerland	Local research staff	
Stefan	Christ			Cantonal Hospital Winterthur	Winterthur, ZH, Switzerlan	Local Co-Investigator	
Cristina	de Basio Marinello			N.A.		Family member expert	
Esther	Doron			Kantonsspital St.Gallen	St. Gallen, SG, Switzerland	Study coordinator	
Susann	Endermann			Kantonsspital St.Gallen	St. Gallen, SG, Switzerland	Local research staff	
Sabine	Fazlic			Hirslanden Clinic Zurich	Zurich, ZH, Switzerland	Intervention nurse	
Kim-Jana	Fehlbier			University Hospital Zurich	Zurich, ZH, Switzerland	Study coordinator	
Debora	Figi			University Hospital Zurich	Zurich, ZH, Switzerland	Local research staff	
Cindy	Groen			Lindenhofspital	Bern, BE, Switzerland	Study coordinator	
Sophie Valentine	Gruber			University Hospital Zurich	Zurich, ZH, Switzerland	Study coordinator	
Gaby	Gürber			Lucerne Cantonal Hospital	Lucerne, LU, Switzerland	Local research staff	
Monika	Hahn			University Hospital Zurich	Zurich, ZH, Switzerland	Local research staff	
Franziska	Hellmann			Cantonal Hospital Graubünden	Chur, GR, Switzerland	Study coordinator	
Stefanie	Henkensmeier			Hirslanden Clinic Zurich	Zurich, ZH, Switzerland	Intervention nurse	
Sandy	Hesselberth			University Hospital Zurich	Zurich, ZH, Switzerland	Local research staff	
Senta	Hug			Cantonal Hospital Baden	Baden, AG, Switzerland	Intervention nurse	
Maria Katharina	Iberl			University Hospital Zurich	Zurich, ZH, Switzerland	Intervention nurse	
Béatrice	Jenny Moser			Inselspital Bern	Bern, BE, Switzerland	Local research staff	
Mandy	Jentsch			Kantonsspital St.Gallen	St. Gallen, SG, Switzerland	Intervention nurse	
Marion	Jourdan			N.A.		Family member expert	
Monica	Julmy			Spital Thurgau Frauenfeld	Frauenfeld, TG, Switzerlan	Local research staff	
Ramona	Kehl			Cantonal Hospital Baden	Baden, AG, Switzerland	Intervention nurse	
Martina	Keller			Cantonal Hospital Winterthur	Winterthur, ZH, Switzerlan	Study coordinator	

Supplemental Online Content: Nonauthor Collaborators
*First name, last name, and suffix (if applicable) are required and will appear in PubMed.

*First Name and Middle Initial(s)	*Last Name	*Suffix (eg, Jr, III)	Academic Degrees	Institution	Location (city, state/province, country)	Role or Contribution, eg, chair, principal investigator	Group (if more than 1 Group listed in the byline) and/or Subgroup (eg, Steering Committee)
Lea	Kinteh-Vischherr			University Hospital Zurich	Zurich, ZH, Switzerland	Intervention nurse	
Nadine	Kipfer			Hospital of Thun	Thun, BE, Switzerland	Intervention nurse	
Carsten	Klein			Kantonsspital St.Gallen	St. Gallen, SG, Switzerland	Study coordinator	
Eva-Maria	Kleinert			University Hospital Zurich	Zurich, ZH, Switzerland	Study coordinator	
Patrick	Leute			Cantonal Hospital Baden	Baden, AG, Switzerland	Local reserach staff	
Cornelia	Lips			Cantonal Hospital Winterthur	Winterthur, ZH, Switzerlan	Study coordinator	
Lisa Elena	Loparco			University Hospital Zurich	Zurich, ZH, Switzerland	Local research staff	
Roger	Lussmann			Hirslanden Clinic Zurich	Zurich, ZH, Switzerland	Local Principal Investigator	
Gabriela	Manetsch			Cantonal Hospital Graubünden	Chur, GR, Switzerland	Study coordinator	
Matthias	Moos			Spital Thurgau Frauenfeld	Frauenfeld, TG, Switzerlan	Local research staff	
Andrea	Müller Paul			Lindenhofspital	Bern, BE, Switzerland	Intervention nurse	
Ernst	Näf			Solothurn Hospitals AG	Olten, SO, Switzerland	Study coordinator	
Friederike	Nellessen			University Hospital Zurich	Zurich, ZH, Switzerland	Local research staff	
Dunja	Nery Barreto			Inselspital Bern	Bern, BE, Switzerland	Local research staff	
Priska	Odelli			Cantonal Hospital Baden	Baden, AG, Switzerland	Local reserach staff	
Saskia	Oesch			University of Zurich	Zurich, ZH, Switzerland	Doctoral student	
Eva	Pietzke			Cantonal Hospital Baden	Baden, AG, Switzerland	Local reserach staff	
Claudio	Rebelo			University Hospital Zurich	Zurich, ZH, Switzerland	Local research staff	
Jasmin	Rüesch			Cantonal Hospital Graubünden	Chur, GR, Switzerland	Intervention nurse	
Jacqueline	Rütsche			Kantonsspital St.Gallen	St. Gallen, SG, Switzerland	Intervention nurse	
Franziska	Rutz			Cantonal Hospital Baden	Baden, AG, Switzerland	Study coordinator	
Sonia	Santini			Hospital of Thun	Thun, BE, Switzerland	Intervention nurse	
Christian	Schandl			Cantonal Hospital Winterthur	Winterthur, ZH, Switzerlan	Local Co-Investigator	
Sarah	Schmidt			Cantonal Hospital Baden	Baden, AG, Switzerland	Intervention nurse	
Irina	Schwenk			University Hospital Zurich	Zurich, ZH, Switzerland	Local research staff	
Nadine	Schwindt			University Hospital Zurich	Zurich, ZH, Switzerland	Local research staff	
Yvonne	Seiffert			Lindenhofspital	Bern, BE, Switzerland	Intervention nurse	
Erika	Sigrist			Lindenhofspital	Bern, BE, Switzerland	Implementation Support	
Ursina	Spörri			Cantonal Hospital Winterthur	Winterthur, ZH, Switzerlan	Study coordinator	
Marion	Springer			Spital Thurgau Frauenfeld	Frauenfeld, TG, Switzerlan	Local Co-Investigator	
Christa	Stalder			Hospital of Thun	Thun, BE, Switzerland	Study coordinator	
Birgit	Steiger			Hirslanden Clinic Zurich	Zurich, ZH, Switzerland	Local Principal Investigator	

Supplemental Online Content: Nonauthor Collaborators
*First name, last name, and suffix (if applicable) are required and will appear in PubMed.

***First Name and Middle Initial(s)**	***Last Name**	***Suffix (eg, Jr, III)**	Academic Degrees	Institution	Location (city, state/province, country)	Role or Contribution, eg, chair, principal investigator	Group (if more than 1 Group listed in the byline) and/or Subgroup (eg, Steering Committee)
Michael	Studhalter			Cantonal Hospital Olten	Olten, SO, Switzerland	Local research staff	
Noëlle	Tenner			Cantonal Hospital Graubünden	Chur, GR, Switzerland	Intervention nurse	
Andrea	Thesenvitz			University of Zurich	Zurich, ZH, Switzerland	Trial manager	
Doreen	Trautwein			Cantonal Hospital Baden	Baden, AG, Switzerland	Local reserach staff	
Micaela	Vaerini			Cantonal Hospital Graubünden	Chur, GR, Switzerland	Local research staff	
Merel	van de Westelaken			Cantonal Hospital Winterthur	Winterthur, ZH, Switzerlan	Local Co-Investigator	
Sabine	Vögele			University Hospital Zurich	Zurich, ZH, Switzerland	Local research staff	
Iris	Weber			University Hospital Zurich	Zurich, ZH, Switzerland	Intervention nurse	
Marlene	Wegmann Oswald			Cantonal Hospital Winterthur	Winterthur, ZH, Switzerlan	Study coordinator	
Tobias	Weitbrecht			Cantonal Hospital Graubünden	Chur, GR, Switzerland	Implementation Support	
JoEllen	Welter			Spital Thurgau Frauenfeld	Frauenfeld, TG, Switzerlan	Study coordinator	
Jeannina	Wiedmer			Hospital of Thun	Thun, BE, Switzerland	Intervention nurse	
Deborah	Wolf			Cantonal Hospital Baden	Baden, AG, Switzerland	Intervention nurse	
Dirk	Würzberg			Univesity Hospital Zurich	Zurich, ZH, Switzerland	Intervention nurse	
Andrea	Zimmermann			Kantonsspital St.Gallen	St. Gallen, SG, Switzerland	Implementation Support	
Thomas	Zurbrügg			N.A.		Patient expert	
