# Supplementary material for: Nurse-Led Family Support Intervention for Families of Critically Ill Patients: The FICUS Cluster Randomized Clinical Trial
Source: JAMA Intern Med. 2025 Jul 28;185(9):1138–49. doi: 10.1001/jamainternmed.2025.3406 (PMC12558129; doi:10.1001/jamainternmed.2025.3406)
Supplement: Supplement 4. — Data Sharing Statement [file jamainternmed-e253406-s004.pdf]

Data Sharing Statement

Naef. Nurse-Led Family Support Intervention for Families of Critically Ill Patients. *JAMA Intern Med*. Published July 28, 2025. doi:10.1001/jamainternmed.2025.3406

Data
Additional Information: Cinicaltrials.gov, Identifier: NCT 05280691
Data available: Yes
Data types: Deidentified participant data
How to access data: Data will be made available on a public data repository, such as Zenodo
When available: beginning date: 06-30-2026

Supporting Documents
Document types: Study protocol and statistical analysis plan
How to access documents: https://trialsjournal.biomedcentral.com/articles/10.1186/s13063-022-06454-y and https://trialsjournal.biomedcentral.com/articles/10.1186/s13063-024-08351-y
When available: With publication

Additional Information
Who can access the data: Data will be shared with investigators or researchers who provide a methodologically sound proposal and whose proposed use of the data has been approved by an independent review identified for this purpose
Types of analyses: For a specified purpose
Mechanisms of data availability: After approval of a proposal
Any additional restrictions: Only data of participants who have provided consent for reuse of data will be given
